# Aboveground vs. Belowground Carbon Stocks in African Tropical Lowland Rainforest: Drivers and Implications

**DOI:** 10.1371/journal.pone.0143209

**Published:** 2015-11-24

**Authors:** Sebastian Doetterl, Elizabeth Kearsley, Marijn Bauters, Koen Hufkens, Janvier Lisingo, Geert Baert, Hans Verbeeck, Pascal Boeckx

**Affiliations:** 1 Ghent University, Isotope Bioscience Laboratory - ISOFYS, Ghent, Belgium; 2 Harvard University, Department of Organismic and Evolutionary Biology, Cambridge, United States of America; 3 University of Kisangani, Faculty of Sciences, Kisangani, DR Congo; 4 Ghent University, Department of Applied Biosciences, Ghent, Belgium; 5 Ghent University, Computational & Applied Vegetation Ecology - CAVElab, Ghent, Belgium; 6 Augsburg University, Department of Geography, Augsburg, Germany; Chinese Academy of Forestry, CHINA

## Abstract

**Background:**

African tropical rainforests are one of the most important hotspots to look for changes in the upcoming decades when it comes to C storage and release. The focus of studying C dynamics in these systems lies traditionally on living aboveground biomass. Belowground soil organic carbon stocks have received little attention and estimates of the size, controls and distribution of soil organic carbon stocks are highly uncertain. In our study on lowland rainforest in the central Congo basin, we combine both an assessment of the aboveground C stock with an assessment of the belowground C stock and analyze the latter in terms of functional pools and controlling factors.

**Principal Findings:**

Our study shows that despite similar vegetation, soil and climatic conditions, soil organic carbon stocks in an area with greater tree height (= larger aboveground carbon stock) were only half compared to an area with lower tree height (= smaller aboveground carbon stock). This suggests that substantial variability in the aboveground vs. belowground C allocation strategy and/or C turnover in two similar tropical forest systems can lead to significant differences in total soil organic C content and C fractions with important consequences for the assessment of the total C stock of the system.

**Conclusions/Significance:**

We suggest nutrient limitation, especially potassium, as the driver for aboveground versus belowground C allocation. However, other drivers such as C turnover, tree functional traits or demographic considerations cannot be excluded. We argue that large and unaccounted variability in C stocks is to be expected in African tropical rain-forests. Currently, these differences in aboveground and belowground C stocks are not adequately verified and implemented mechanistically into Earth System Models. This will, hence, introduce additional uncertainty to models and predictions of the response of C storage of the Congo basin forest to climate change and its contribution to the terrestrial C budget.

## Introduction

The Tropics are currently facing unseen changes due to population growth, continuous development of economic infrastructure and, ultimately, land use change through deforestation from natural tropical rainforest system to systems used for agriculture and forest plantations. At the same time, the Tropics are a hotspot of Global Warming, putting these vulnerable ecosystems under additional stress [1,2].

Forests are considered the most productive terrestrial ecosystems on earth, containing no less than 45% of the terrestrial carbon stock [3], and have been increasingly recognized as a key player in global climate change mitigation [4–6]. This holds especially for tropical forests, accounting for approximately 55% of this global stock in forests, with the Amazon basin and the Congo basin being the largest two contiguous blocks [7]. Research efforts from the last decade have addressed the need for large-scale forest monitoring networks in the tropics [8,9] to gain insight in the spatial variability of the carbon stocks, and hence reduce the uncertainty in regional and global estimates and modeling efforts [10, 11]. However, most reports strongly focus on the above-ground carbon stocks, making rudimentary assumptions for the belowground stocks. There is still a lack of knowledge on soil organic carbon (SOC) stocks in tropical forest systems, their controls and the relationship of biomass allocation and SOC stocks [8, 12–13]. Although uncertainties are large and the soil compartment only compromises around 32% of the carbon stock in the total ecosystem in tropical forests [7], tropical evergreen forests are probably the biomes with the biggest total SOC storage worldwide (474 Pg C [14]). This represents an equivalent of about 63% of the total atmospheric C pool (760 Pg C [15]).

The processes that control carbon sequestration in soils and plants of the tropics are likely underlying a different dynamic than those of boreal or temperate forests, as carbon (C) cycling in the Tropics is not constrained by climatic factors such as the availability of heat and water. In addition, it often takes place in a nutrient depleted environment due to the highly weathered state of soils [16] after millions of years of soil weathering. Additionally, while it is well known that land use change and forest management can affect the SOC stocks in the Tropics [17,18], much less is known of the effect of tree composition and edaphic gradients on soil carbon stocks, although tree species effects on soil carbon storage are considerable [19,20]. A study for ten Amazonian forests plots reported that the ratio belowground to total NPP remains fairly invariant across a soil fertility gradient [21].

In our study we investigate the aboveground vs. belowground carbon stocks in similar tropical lowland rainforest on two nearby locations in the Congo basin and explore potential drivers behind differences in C allocation and retention. For this, we combine an assessment of the aboveground biomass, including species composition and growth characteristics, with a depth explicit assessment of SOC stocks in these systems including soil geochemical parameters related to soil fertility and an assessment of C stabilization in different functional pools.

## Methods

### Study area

This study was carried out in biosphere reserves nearby Kisangani, Democratic Republic of the Congo (DRC). Two sites, approx. 100km apart, within this region with similar climatic and topographic conditions as well as plant community structure have been selected. A first site is located in the UNESCO Man and Biosphere reserve in Yangambi (YGB; N00°48’; E24°29’) [22], approx. 100 km west of Kisangani, just north of the Congo river. A second site in the Yoko reserve (YOKO) has been selected approx. 28 km south of Kisangani (N00°17’; E25°18’) [23]. Permission to conduct research at these sites has been given by the National Institute for Agronomical Research and Education of the Democratic Republic of Congo (INERA-DRC) and the University of Kisangani. The field studies did not involve endangered or protected species.

Vegetation in this region is characterized by moist semi-deciduous rainforest, with fragments of moist evergreen rainforest, transition forest, agricultural land, fallow and swamp forest [24]. Following the revised Köppen-Geiger classification [25], the climate of the region is described as Af-type tropical rainforest climate. As measured in the YGB meteorological station, the region receives an annual precipitation of 1839.5 ± 205.7 mm (1980–2012) with an average dry season length of 3.3 ± 1.3 months with monthly precipitation lower than 100 mm, during December–February and June-August. Temperatures are high and constant throughout the year with a minimum of 24.2 ± 0.4°C in July and a maximum of 25.5 ± 0.6°C in March. The topography of the region is gentle, with little differences in elevation of less than 10 m between both sites (approx. 471–479 m asl). Dominant soils in the area are nutrient poor and deeply weathered Ferralsols, formed from fluvio-aeolian sediments, composed mostly of quartz sand, kaolinite clay and hydrated iron oxides [26].

### Plot characterization

At both sites five 1 ha plots are inventoried in semi-deciduous mixed forest in 2012 and 2013 in Yangambi and Yoko, respectively. Plot locations at each site were selected at random within the old-growth forest, with a minimum distance of 300 m between the edges of the plots. All trees with a diameter at breast height (DBH) larger or equal to 10 cm have been measured and identified to species level. Buttressed trees, although a rarity in the region, and stilt-rooted trees are measured 50cm above the highest root, where the trunk shape is cylindrical. When a deformity is present at breast height, the diameter is measured 2 cm lower. Species identification was done with the help of local botanists of the Institut National pour l’Etude et la Recherche Agronomiques (INERA). At YGB, tree heights are measured on a subset of trees covering species which are within 95% of the basal area of each plot, with two individuals of each species randomly selected within each designated diameter class of 10–20, 20–30, 30–50 and >50 cm DBH, when possible. In the second site (YOKO), tree height was measured for all trees in smaller plots of 0.25 hectare. All heights were measured using a Nikon Laser Rangefinder Forestry Pro hypsometer (Nikon Corporation, Japan). A more detailed comparison and discussion of forest structure, species composition and light availability at the two sites is available in S1 File.

### Tree height and aboveground biomass estimation

Site-specific height-diameter regression models were developed for each forest type. All trees known to be broken, damaged or leaning more than 10% were excluded from the analysis. Site-specific height-diameter models were set up, for which the three-parameter exponential height–diameter model was selected as optimal model at both sites independently (see S1 File):

H = a − bexp(−cDBH)

for which H is the total measured tree height corresponding to the DBH of the individual; a, b and c are the curve parameters optimized for each site, which represent, respectively, the maximum asymptotic height, the difference between minimum and maximum height, and shape of the curve [27]. These models were further used to determine tree heights for aboveground carbon (AGC) stock estimation. Despite the differences in plot size between YGB and YOKO, roughly the same number of trees are measured in height with a similar distribution over the different diameter sizes. The pan-tropical relation of [28] including height and wood density was selected for AGC stock estimation, with biomass assumed to be 50% carbon. Site-specific wood density measurements were used for YGB (Kearsley et al. 2013), and completed with genus level averages if species level data was not available. For species not determined in YGB, values from the Global Wood Density Database [29, 30] were used. For the remaining individuals for which no wood density was available, a wood density value was assigned through random sampling of wood density from other individuals within the same site.

### Soil and litter sampling and analysis

On two plots per study site, ten soil cores have been taken and composed to three depth increments (0–30, 30–60, 60–90) and oven-dried (50°C). At each of the two study plots where soil samples were taken, forest floor litter was sampled in May 2014 from a randomly distributed 0.5 x 0.5 m square in triplicate to capture the variability in plant growth/litterfall in the area. The forest in YOKO and YGB shows a seasonal pattern in litter production for fine litter (4.57 Mg C ha^-1^ yr^-1^) and foliar litter (2.85 Mg C ha^-1^ yr^-1^), with the highest rates of litterfall occuring January-March and September-October (Cassart pers. Comm.). This litterfall dynamic is mainly driven by the region`s climatic seasonality [31].

After sampling, litter has been dried at 40°C and the dry weight has been taken. For both soil and litter the following parameters have been measured: bulk density (soil only), soil texture, pH (soil only), potential cation exchange capacity, base saturation, bioavailable P, NO3 and NH4, C stock and soil organic carbon fractions.

#### Bulk density and soil texture

Bulk density was determined on composites of 10 samples per plot using Kopecky cylinders. Soil texture was determined by means of the percentage of sand, silt and clay. Analyses were performed on air-dried soil fractions (<2 mm). The sand fraction (>63 μm) was separated by wet sieving; the silt and clay fractions were determined by the Köhn pipette method after dispersion with sodium hexametaphosphate [32].

#### pH, CEC and BS

Soil pH was determined potentiometrically in 25 ml 0.01 M CaCl_2_ (1:2.5 soil:solution ratio) with a glass electrode using a portable multi-parameter Meter HI9828 (Hanna Instruments US Inc., USA). Potential cation exchange capacity (CEC_pot_) was determined by quantifying NH_4_^+^ exchanged with 2 M KCl after saturating cation exchange sites with ammonium acetate buffered at pH 7.0 and measured with ICP-MS. Exchangeable Al was extracted by 1 M KCl solution and determined colorimetrically. The total percent base saturation (BS), defined as the relative availability of each cation for CEC_pot_, was calculated in percent of CEC_pot_.

#### Bioavailable N and P

Resin-extractable P was determined in the bulk soil using resin-impregnated membrane strips whereas P in litter was determined by dry ashing. NH_4_^+^ and NO_3_^−^ were determined in a 1 M KCl extract (ratio 2:1) and were measured from filtrates using a continuous flow analyzer (FIAstar 5000, Foss, Denmark) for both soil and litter.

#### SOC Fractionation

**Fig 1:**
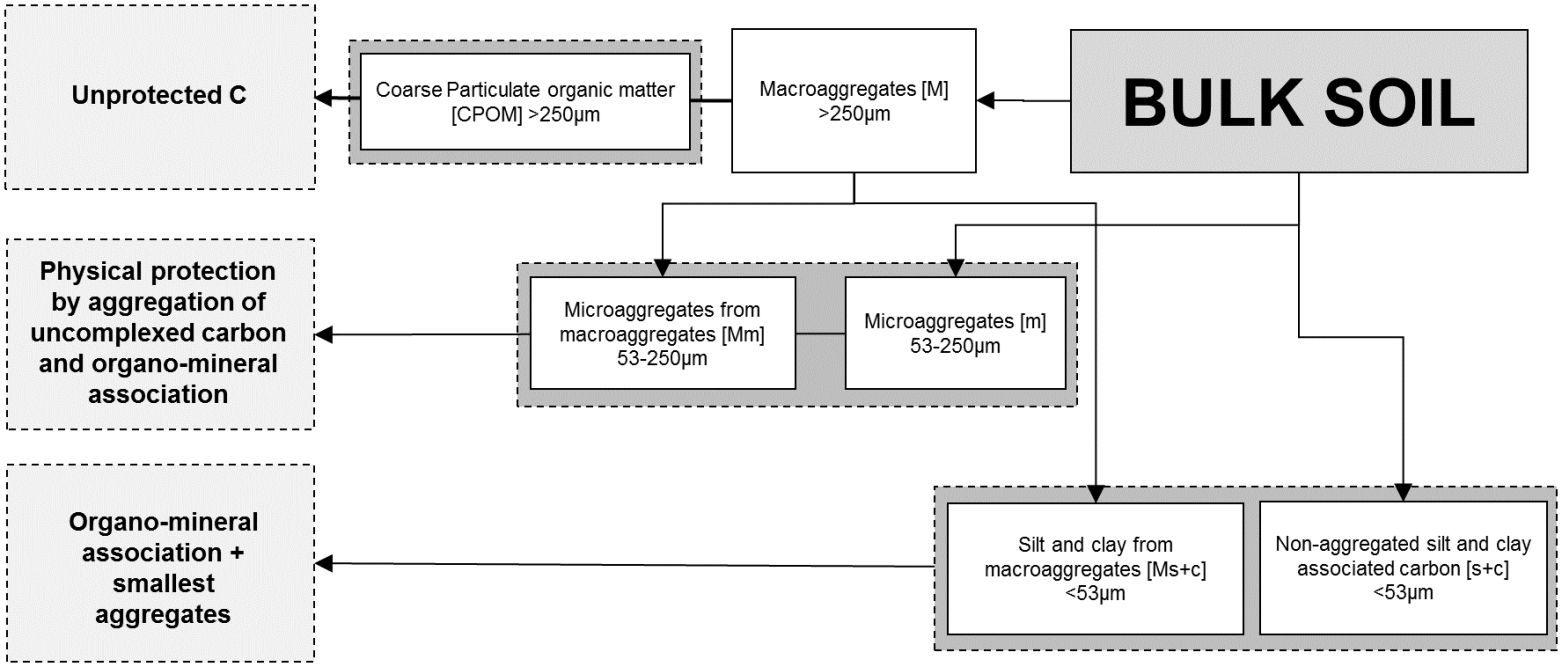
Applied fractionation scheme to derive SOC fractions and their functional interpretation in terms of present stabilization mechanisms.

To quantify the distribution of SOC fractions at both study sites, the soil samples were gently broken into smaller pieces by hand, sieved through an 8 mm sieve to get a homogenous substrate with the inherent aggregate structure remaining largely undisturbed [33]. A subsample of 100 g of this homogenized soil was then used in the SOC fractionation analysis. We used a method based on the conceptual SOC fraction model proposed by Six et al. [33,34] (Fig 1). The scheme consists of a series of physical fractionation techniques applied to isolate functional SOC fractions, differentiated by stabilization mechanisms (chemical, biochemical, and physical), which can also be associated with different turnover times and SOC stability [34–37]. In a first step, SOC is fractionated into macroaggregate (>250 μm) (M), microaggregate (250–53 μm) (m), and free silt & clay (<53 μm) (s+c) fractions by slaking to define water-stable aggregates and no dispersion agent has been used. Then, the macroaggregate fraction gets further processed to derive more subcompartments (see Fig 1 [34]), namely the coarse particulate organic matter (CPOM), microaggregates within macroaggregates (Mm) and silt and clay within macroaggregates (Ms+c). Soil mass and C concentrations for all aggregate fractions have been sand-corrected and we modified the original scheme by not separating the sample into a light and a heavy C fraction using density flotation, as the light fraction is typically very small at sites with low carbon content [38,39].

#### SOC and litter C concentration and mass

SOC concentration was measured in 1 g ground subsamples using a dry combustion analyzer (Variomax CN, Elementar GmbH, Hanau, Germany) with a measuring range of 0.2–400 mg C g^-1^ soil (absolute C in sample) and a reproducibility of <0.5% (relative deviation). Recovery rates exceeding 97% and 91% were obtained for the soil mass and C mass, respectively, across all fractions. The isolated fractions were analyzed for total SOC using an elemental analyzer (ANCA-GSL PDZ Europa, Crewe, UK) coupled to an Isotope Ratios Mass Spectrometer (2020, SerCon, Crewe, UK). For each depth increment, carbon stocks (Mg C ha^−1^) were determined as a product of bulk density (g cm^−3^), carbon concentration (mg g^-1^) and thickness of the increment layer (cm). For the litter, carbon stocks were determined as the product of the litter mass per area and the litter C concentration.

### Statistics

Statistical tests for differences between the means of assessed soil variables between the two sites have been performed for the whole dataset and for top and subsoil samples separately using Bonferroni corrections and Tamhane’s T2 with SAS Enterprise 4.2 (SAS Institute Inc., Cary, NC, USA). This resulted in a total of 12 observations for each physical or chemical soil variable for the bulk soil. For each SOC fraction 18 observations were available as we randomly duplicated half of the fractionation experiment to check for the repeatability of the method and assess the method related error.

## Results

### Forest structure and aboveground carbon storage

Both forests are highly similar in species composition, species diversity and aboveground forest structure concerning diameter distributions and light availability (see S1 File), and no significant differences are found between stem density and basal area (Table 1). However, a significant difference is found in AGC storage, 17% higher in YOKO (Table 1). Difference in AGC storage can be attributed to a difference in tree height where the maximal asymptotic height of the H-D model indicates higher values in YOKO (≈42m) compared to YGB (≈36m).

**Table 1:** Stand characteristics, aboveground living biomass carbon (AGC) and location of the two sites^a^.

	YGB	YOKO
**Stand characteristics**		
Stem density (ha^-1^)	419 ± 89 (a)	469 ± 35 (a)
Basal area (m^2^ ha^-1^)	32 ± 3 (a)	34 ± 3 (a)
** AGC (Mg C ha** ^**-1**^ **)**	**163 ± 19 (a)**	**191 ± 28 (b)**
H-D model (H = a–b.e^-cD^)	a 36.358; b 31.659; c 0.022	a 42.502; b 39.147; c 0.020
**Site Coordinates**		
Latitude (d.dddd)	0.7995	0.2918
Longitude (d.dddd)	24.5077	25.3113
Altitude (m asl)	479 ± 13	471 ± 5
Plotsize (ha)	1	0.25–1

^a^Regression model: For each parameter, significance from t-test is provided between brackets comparing the two sites. Values within a row not sharing a common letter differ significantly (p > 0.01).

### Physical and chemical soil parameters

Soil texture at both study sites is similar and characterized by a predominantly sandy matrix (˃80% sand content) (Table 2). Bulk density at YOKO and YGB were measured with an average of 1.2 g cm^-3^ and 1.5 g cm^-3^, respectively. Typical for these type of tropical soils, pH values were low and highly acidic (4.0–4.6) accompanied with very low CEC_pot_ values (2.9–5.3 meq 100g^-1^). Exchangeable Al ranges between 15–52 ppm, decreasing with soil depth. Base saturation of CEC_pot_ did not exceed 30% and was generally lower in YGB than in YOKO and dominated by Ca (13–21%) (details not shown). While the concentration of the base cations Ca and Mg were similar between the study sites (7.4–137.5 ppm), K concentrations in the soil solution were more than doublein YOKO (31.3–68.9 ppm) compared to YGB (14.9–34.2 ppm), and Na concentrations were about 50–70% higher in YOKO (12.0–13.3 ppm) compared to YGB (7.6–7.8 ppm). Nitrate-N (9.68–1.98 g kg^-1^), Ammonia-N (9.54–1.95 g kg^-1^) and bioavailable P (3.3–8.9 g kg^-1^), decreased with soil depth but showed no significant difference (p>0.05) in concentrations between both sites.

**Table 2:** Physical and chemical soil parameters for both sites and different depths^a^.

	Soil	CEC	Base cations in CEC				CEC	exchang, Al	pH_KCL_	Texture (mass %)			Nitr-N	Amm-N	Bio-P	Bulk density
	Depth	Base saturation	Ca	K	Mg	Na				Sand	Silt	Clay				
		%	ppm				meq 100g^-1^	ppm	-	2000–63μm	63–2 μm	˂2μm	g kg^-1^			g cm^-3^
YOKO	0–30	29±2.4	137.5±0.7	68.9±13.0	12.9±1.3	12.0±1.4	3.5±0.1	52.3±25.7	4.0±0.2	83.2±4.1	3.7±0.6	13.1±3.5	9.68±0.51	8.56±0.48	7.62±2.94	1.19±0.08
	30–60	29±4.0	125.0±5.2	47.8±12.0	9.2±0.8	12.8±2.0	3.1±0.1	17.8±15.0	4.5±0.3	82.1±2.2	3.7±0.7	14.3±1.5	2.71±0.14	2.57±0.14	3.34±0.33	1.2±0.06
	60–90	30±1.4	120.5±6.4	31.3±	8.1±0.2	13.3±1.4	2.9±0.4	14.5±6.5	4.6±0.2	80.1±2.2	5.7±1.4	14.3±0.8	3.52±0.18	1.95±0.10	3.33±0.54	1.23±0.03
YGB	0–30	18±0.7	131.5±4.9	34.2±1.3	15.9±0.8	7.7±0.3	5.3±2.1	57.7±59.9	4.0±0.4	85.1±1.7	1.9±0.2	13.1±1.7	8.19±0.41	9.54±0.48	8.89±1.69	1.39±0.21
	30–60	20±1.7	126.0±5.7	18.1±6.4	9.4±1.2	7.5±0.5	4.0±1.3	45.1±28.8	4.2±0.3	83.5±2.8	2.6±0.6	13.9±3.5	4.56±0.22	4.91±0.25	5.91±2.70	1.53±0.15
	60–90	19±1.5	125.0±1.4	14.8±9.3	7.4±0.4	7.7±0.7	4.1±0.4	40.0±32.3	4.3±0.2	80.5±3.6	1.7±0.0	17.8±3.7	1.89±0.10	3.37±0.17	3.70±0.58	1.52±0.19

^a^Abbreviations: CEC = soil potential Cation Exchange Capacity; Nitr-N = Nitrate N; Amm-N = Ammonia N; Bio-P = Bioavailable Phosphorus; Mass = Litter mass; C_Stock_ = Litter C stock.

### Litter parameters

While differences in litter mass (4.8–4.9 Mg ha^-1^) and litter C stock (1.8–2.0 Mg C ha^-1^) between both sites were insignificant, distinct differences in litter quality between both study sites have been found (Table 3). In general, concentrations of Ca, Mg and K cations in the CEC extract were between 18 and 58% lower in litter from YOKO compared to YGB and ranged between 1044–5143 ppm. In contrast, Na concentrations were two magnitudes lower (34–46 ppm) and differed only marginally between the two sites. While bioavailable P (0.80–0.84 g kg^-1^) and Ammonia-N concentrations (0.68–0.63 g kg^-1^) were similar for the litter of the different sites, Nitrate-N concentrations were about 86% higher at YOKO (1.98g kg^-1^) compared to YGB (1.06g kg^-1^). However, this difference remains insignificant (p > 0.05) due to large variability between replicates at both sites. Average C concentration in the litter from YOKO was less (369 g kg^-1^) than in the litter from YGB (410 g kg^-1^) while CN ratios were similar (20.9–19.6).

**Table 3:** Quantitative and qualitative litter parameters at both study sites^a^.

	CEC	Base cations in CEC				CEC	Nitr-N	Amm-N	Bio-P	Mass	C_STOCK_
	Base saturation	Ca	K	Mg	Na						
	%	ppm				meq 100g^-1^	g kg^-1^			Mg ha^-1^	Mg C ha^-1^
YOKO	75.0±24.1	4217.8±1181	1720.9±645	1044.2±423	46.3±13	46.8±5.0	1.98±0.97	0.68±0.22	0.80±0.11	4.9±1.1	1.8±0.4
YGB	81.9±23.6	5143.3±1630	2271.7±212	2448.3±1007	34.15±5	60.45±5.3	1.06±0.40	0.63±0.14	0.84±0.11	4.8±1.7	2.0±0.7

^a^CEC = soil potential Cation Exchange Capacity; Nitr-N = Nitrate N; Amm-N = Ammonia N; Bio-P = Bioavailable Phosphorus; Mass = Litter mass; CStock = Litter C stock.

### SOC and Fractions

**Fig 2:**
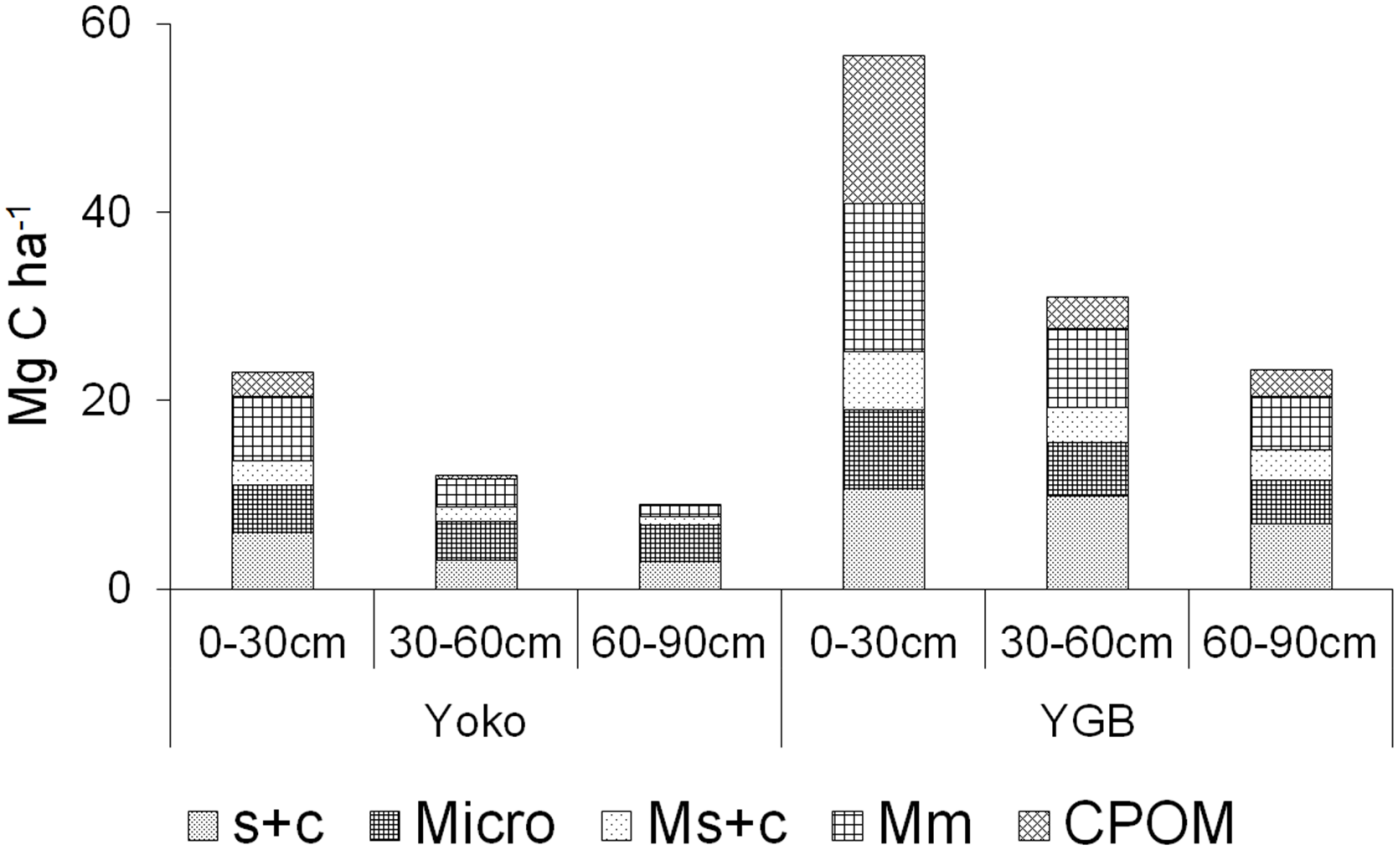
Relative contribution of isolated fractions to SOC_Stock_ per ha for the specific depth layer.

The highest C concentrations were found in the CPOM fraction, on average 2–3 times higher than in comparable mineral associated fractions for all measured depth increments. Despite the described similarity in forest composition, litter mass and soil properties, SOC stocks at YOKO (Table 4) (44.2±4.0 Mg C ha^-1^) were less than half of the stock of YGB (109.5±21.4 Mg C ha^-1^) and significantly different between depth increments and between the two study sites. These discrepancies are related to differences in C concentrations of the aggregate and CPOM fractions (Table 1) and the abundance of these fractions in the different soils and soil layers (Fig 2).

**Table 4:** Carbon concentrations for litter, bulk soil and SOC fractions and the litter and soil C_STOCK_ ^a^.

		C							C_STOCK_
		mg g^-1^							Mg C ha^-1^
		Bulk	Macro	Micro	s+c	CPOM	Mm	Ms+c	Bulk
YOKO	Litter	369.0±81.5^A^	-	-	-	-	-	-	1.8±0.39^A^
Depth [cm]	0–30	6.5±0.09^a^	14.9±2.69^a^	17.5±0.59^a^	24.5±2.64^a^	83.6±5.25^a^	28.8±1.16^a^	31.3±2.64^a^	23.1±1.9^a^
	30–60	3.4±0.31^b^	10.9±2.54^b^	12.5±0.08^b^	18.0±0.84^b^	51.6±2.95^b^	13.2±1.19^b^	17.2±0.84^b^	12.2±1.8^b^
	60–90	2.4±0.02^c^	4.1±0.42^c^	9.9±0.02^c^	14.9±0.07^c^	36.3±8.32^c^	10.3±1.35^c^	14.9±0.07^c^	8.90±0.3^c^
YGB	Litter	409.8±145.0^A^	-	-	-	-	-	-	1.7±0.70^A^
Depth [cm]	0–30	13.4±3.13^d^	74.3±15.54^d^	51.2±12.9^d^	34.0±7.77^d^	153.0±55.2^d^	47.4±2.97^d^	34.0±7.77^d^	55.7±13.1^d^
	30–60	6.7±0.38^a^	32.7±0.67^e^	21.5±2.89^e^	25.7±0.71^a^	77.4±29.76^abe^	31.4±3.36^e^	25.7±0.71^e^	30.8±4.8^e^
	60–90	5.0±0.13^e^	27.5±1.53^f^	14.9±0.37^f^	18.0±1.53^b^	64.7±3.43^e^	21.4±0.90^f^	18.0±1.53^b^	23.0±3.5^a^

^a^Superscripted letters indicate ANOVA test results for significant differences (p < 0.05) of C concentrations and C_STOCK_ between sites and depths. Fractions and bulk soil have been tested separately for all parameters. Same letters indicate no significant difference between depths and/or sites. Abbreviations: Bulk = Bulk soil C; Macro = Macroaggregate associated C; Micro = Microaggregate associated C; s+c = free silt and clay associated C; CPOM = Coarse particular organic C; Mm = Microaggregate within Macroaggregates associated C; Ms+c = silt and clay within Macroaggregates associated C.

Soil fractionation (Fig 2) indicates that the non-aggregated silt and clay fraction (s+c) at both sites contributes to the respective total SOC mass in approximately the same amount (19–33% of total SOC mass) with an increasing contribution of non-aggregated silt and clay associated C in subsoils. Differences between sites are more pronounced for the aggregated fractions. The contribution of the free microaggregate associated C (m) to total SOC mass is higher, especially in subsoils, at YOKO (22–42% of total SOC mass) than at YGB (15–20% of total SOC mass).

Between 25–51% of the total SOC mass were stored in macroaggregates (CPOM+Mm+Ms+c) at YOKO, with a relative decrease of about 82% with soil depth. At YGB, between 50–67% of the total SOC mass were stored in macroaggregates with a relative decrease of 69% with soil depth. The gross of these differences is related to significantly higher CPOM related C mass at YGB (12–28% of total SOC mass) compared to YOKO (2–11% of total SOC mass) and higher Mm values at YGB (25–28% of total SOC mass) compared to YOKO (13–30% of total SOC mass) while the contribution of Ms+C at YGB (11–14% of total SOC mass) compared to YOKO (10–13% of total SOC mass) was similar.

## Discussion

### Importance of including SOC stocks in tropical systems

The results demonstrate significant differences in the trends of AGC (17% higher at YOKO compared to YGB) and SOC stocks (50% lower at YOKO compared to YGB) at these two sites with similar species composition and forest structure. While AGC represents 81% of the total C mass at YOKO, this value decreases to ca. 60% at YGB. An assessment of AGC and the aboveground living biomass alone would indicate YOKO as the higher C storing system (Table 5). However, including SOC stocks turns the calculation around showing that the average total C stocks (AGC, litter C and SOC stock combined) at YOKO (237±33 Mg ha^-1^) are lower than those at YGB (275±41 Mg ha^-1^). Even though soil bulk density sampling is prone towards overestimating the soil C pool (5–10% overestimation; expert opinion), this has important implications when assessing the importance of the C storage capacity in tropical rainforest systems. Current monitoring projects such as the UN REDD and REDD+ initiatives [40] focus for central African forest on the carbon stock dynamics in the aboveground biomass. At this level, little attention is paid to SOC stocks in these systems. In the shown example, the combined assessment of SOC and AGC leads to a different conclusion regarding the C storage capacity compared to SOC and AGC assessments separately.

**Table 5:** Summary on average C stocks at both sites in different pools^a^.

	C mass Mg ha ^-1^			
	Litter	SOC	AGC	Total C
YOKO	1.8±0.4	44.2±4.0	191.4±28.3	**237.4±32.8**
YGB	2.0±0.7	109.5±21.4	163.5±18.8	**275.0±40.9**

^a^Abbreviations: Litter = Litter C; SOC = Soil organic carbon; AGC = Aboveground living biomass carbon; Total (Litter+SOC+AGC combined).

### Reasons for SOC stock differences

In a study of ten Amazonian forest sites, Aragão et al. [21] showed that NPP ranges from 9.3±1.3 Mg C ha^−1^ yr^−1^, at a white sand plot, and 17.0±1.4 Mg C ha^−1^ yr^−1^ at a very fertile Terra Preta site, with no apparent relationship between allocation of NPP to below-ground biomass and soil fertility. On average, the forests allocated 64±3% and 36±3% of total NPP to above- and below-ground components respectively, and the ratio of above-ground to below-ground NPP was almost invariant with total NPP. Litterfall and fine root production both increased with total NPP, while stem production showed no overall trend.

Despite these findings, our study suggests that adaptation of plant communities to nutrient limitation by altering the aboveground and belowground C allocation offers the most likely explanation for the remarkable differences observed in AGC to SOC ratio in two similar forest ecosystems. First, the very low nutrient, and especially K levels, at YGB might force plants to allocate a higher amount of biomass into (fine) roots for mining nutrients from this depleted soil [41–44]. This is supported by observations of Wright et al. [45] and Santiago et al. [46] on tropical tree seedling growth responses to nutrient addition, which showed that K might be an overlooked element when it comes to assessing growth patterns of tropical forests. They found significantly reduced allocation of biomass to roots as well as increased height growth for lowland tropical forests after K addition. Our observations from two similar rainforest systems in the Congo basin support this hypothesis, as SOC stocks were found to be higher in the K depleted system (YGB; Tables 2 and 5). Additionally, the similarity of soil texture and the similarity in the amount of silt and clay associated C between both sites indicates that soil mineralogy, in terms of supporting the stabilization of C with minerals [34,37], does not play a significant role in explaining the observed differences in C stocks between YGB and YOKO (Tables 2, 4 and Fig 2). On the contrary, the higher C input in soils at YGB, as indicated by the higher CPOM content, compared to YOKO promotes the formation of macroaggregates even at greater depths, while at YOKO macroaggregates decrease in importance for SOC stocks with depth (Fig 2). The higher (macro)aggregate associated C content at YGB is largely driven by coarse particular organic matter (CPOM) and microaggregate associated C (Mm). The formation of these two fractions is generally regarded to heavily rely on plant litter and root residues [47–49]. Assuming the same mechanism takes place here, the high CPOM and Mm contribution to SOC stocks is an additional support for the previously stated hypothesis of increased allocation of biomass to roots in the nutrient (K) depleted system (YGB), consequently leading to higher SOC stocks. Note that both sites are characterized by very poor soil fertility conditions, indicated by very low CEC content, base saturation of the CEC and soil texture (Table 2). Hence, in nutrient depleted soil systems, small changes in limiting factors for plant growth can have a large effect, which is not necessarily to be expected in more fertile soils.

However, this is only one of several possible explanations for the observed difference. Nutrient limitation could also influence microbial activity and C decomposition [50–55].

While the poor soil conditions (sandy texture, low pH, low CEC) at both sites are arguably factors which would constrain microbial activity, it is unclear in how far this can be related to differences in SOC stocks here. Aragão et al. [21] suggest that root turnover rates are a consequence of soil fertility, which may be higher in high fertility soils and lower in low fertility soils [56,57]. Another possibility is that higher bulk density at YGB compared to YOKO (Table 2) might constrain aeration and increase water saturation, leading to anaerobic conditions for parts of the soil matrix and, hence, lower decomposition rates which favor SOC accumulation at YGB over YOKO. However, no data on microbial activity is available for our study sites, so a potential connection between SOC stocks and constrained microbial activity remains unverified. Other causal explanations for the difference in SOC/AGC ratios between the sites could be demographic processes (= average tree lifespan or root lifespan between both sites) and intra/inter-specific competition for water and light, mediated by above ground tree functional trait characteristics (e.g. [58]). Root lifespan, for example, is drastically affected by nutrient availability, with nutrient depleted soils showing thinner roots with lower lifespans, and hence, potentially higher C inputs to soils [59]. Finally, while we assume our sites to be pristine and low disturbance, we cannot completely exclude unknown disturbance as a driver for the observed patterns. For Amazonian forest systems, disturbance has been shown to change C allocation patterns [8] towards higher levels of aboveground NPP. Data and results from this paper cannot settle this argument and most likely a combination of cross-correlated factors have to be considered.

In summary, in nutrient limited systems like the investigated tropical rainforest of this study, small changes in nutrient availability, especially K, seem to have large consequences for SOC stocks by governing the allocation of biomass by plants for mining nutrients from soil and litter [45,60]. As a result, carbon is pumped into different parts of the ecosystem with potentially fast (litter) or slower (SOC) turnover. A potential constraining effect of nutrient limitation on microbial activity and, in consequence, the decomposition of organic matter, remains likely but unverified.

## Conclusion

In conclusion, our data shows differences of more than 100% in SOC stocks between two tropical forest systems with different average tree heights and aboveground biomass (low AGC stocks and smaller trees where SOC stocks are high), but very similar species composition, soil geochemistry and climate. This has important consequences for the assessment of total C stored in those systems leading to different conclusions regarding the C storage capacity of the whole system, i.e. above- and belowground C mass combined. Our observation derived from natural systems supports experimental studies on the effect of nutrient limitation, especially K and N, on SOC stocks and biomass allocation. However, the complex interplay of environmental factors governing the sequestration and release of carbon in plants and soils remains poorly understood. In consequence, as long as these large differences in SOC stocks cannot be adequately represented mechanistically in ecosystem models, all modeling and estimates of the future response of the C storage of tropical forest ecosystems are subject to large uncertainties.

## Supporting Information

S1 Fig Ordinations of species composition on the first two axis of a detrended correspondence analysis. Based on plot scores of each 1 hectare plot (dots), the two sites, Yoko (blue dots) and Yangambi (green dots), show a similar species composition (ANOVA on DCA axis 1 and 2, p > 0.05 and p > 0.1 respectively). The spread of the species scores themselves (red crosses) indicate some variability within the plots.
(PDF)

S2 Fig Distribution of individuals trees in different diameter size classes. Bars represent mean of five hectares in both sites, with standard deviation indicated by line segments.
(PDF)

S1 File Additional information to forest structure, floristic composition, leaf area index, light availability and height-diameter model selection. Includes figures, tables and references.
(DOCX)

S1 Table Stand characteristics (Aboveground carbon (AGC); Leaf area index (LAI)), species diversity and location of the two sites. (PDF)

S2 Table Average number of stems with standard deviation in brackets of tree species per hectare in Yoko within different diameter classes. (PDF)

S3 Table Average number of stems with standard deviation in brackets of tree species per hectare in Yangambi within different diameter classes. (PDF)

S4 Table Tested height-diameter function forms where H is height, D is diameter and a, b and c are constant coefficients to be estimated. (PDF)

S5 Table Parameterization for the different models functions in S4 Table for Yangambi. (PDF)

S6 Table Parameterization for the different models functions in S4 Table for Yoko. (PDF)
