# Correction: Aboveground vs. Belowground Carbon Stocks in African Tropical Lowland Rainforest: Drivers and Implications

**DOI:** 10.1371/journal.pone.0150681

**Published:** 2016-02-26

**Authors:** Sebastian Doetterl, Elizabeth Kearsley, Marijn Bauters, Koen Hufkens, Janvier Lisingo, Geert Baert, Hans Verbeeck, Pascal Boeckx

In multiple instances, the unit g kg^-1^ appears incorrectly. The correct unit in these instances should be mg kg^-1^. Please see the corrected text and Tables 2 and 3 below.

**Table 2:** Physical and chemical soil parameters for both sites and different depths^a^.

	Soil	CEC	Base cations in CEC				CEC	exchang, Al	pH_KCL_	Texture (mass %)			Nitr-N	Amm-N	Bio-P	Bulk density
	Depth	Base saturation %	Ca	K	Mg	Na	meq 100g^-1^	ppm	-	Sand 2000–63μm	Silt 63–2 μm	Clay <2μm				g cm^-3^
			ppm										mg kg^-1^			
YOKO	0–30	29±2.4	137.5±0.7	68.9±13.0	12.9±1.3	12.0±1.4	3.5±0.1	52.3±25.7	4.0±0.2	83.2±4.1	3.7±0.6	13.1±3.5	9.68±0.51	8.56±0.48	7.62±2.94	1.19±0.08
	30–60	29±4.0	125.0±5.2	47.8±12.0	9.2±0.8	12.8±2.0	3.1±0.1	17.8±15.0	4.5±0.3	82.1±2.2	3.7±0.7	14.3±1.5	2.71±0.14	2.57±0.14	3.34±0.33	1.2±0.06
	60–90	30±1.4	120.5±6.4	31.3±	8.1±0.2	13.3±1.4	2.9±0.4	14.5±6.5	4.6±0.2	80.1±2.2	5.7±1.4	14.3±0.8	3.52±0.18	1.95±0.10	3.33±0.54	1.23±0..03
YGB	0–30	18±0.7	131.5±4.9	34.2±1.3	15.9±0.8	7.7±0.3	5.3±2.1	57.7±59.9	4.0±0.4	85.1±1.7	1.9±0.2	13.1±1.7	8.19±0.41	9.54±0.48	8.89±1.69	1.39±0.21
	30–60	20±1.7	126.0±5.7	18.1±6.4	9.4±1.2	7.5±0.5	4.0±1.3	45.1±28.8	4.2±0.3	83.5±2.8	2.6±0.6	13.9±3.5	4.56±0.22	4.91±0.25	5.91±2.70	1.53±0.15
	60–90	19±1.5	125.0±1.4	14.8±9.3	7.4±0.4	7.7±0.7	4.1±0.4	40.0±32.3	4.3±0.2	80.5±3.6	1.7±0.0	17.8±3.7	1.89±0.10	3.37±0.17	3.70±0.58	1.52±0.19

^a^Abbreviations: CEC = soil potential Cation Exchange Capacity; Nitr-N = Nitrate N; Amm-N = Ammonia N; Bio-P = Bioavailable Phosphorus; Mass = Litter mass; C_Stock_ = Litter C stock.

**Table 3:** Quantitative and qualitative litter parameters at both study sites^a^.

	CEC	Base cations in CEC				CEC	Nitr-N	Amm-N	Bio-P	Mass	C_STOCK_
	Base saturation %	Ca	K	Mg	Na	meq 100g^-1^				Mg ha^-1^	Mg C ha^-1^
		ppm					mg kg^-1^				
YOKO	75.0±24.1	4217.8±1181	1720.9±645	1044.2±423	46.3±13	46.8±5.0	1.98±0.97	0.68±0.22	0.80±0.11	4.9±1.1	1.8±0.4
YGB	81.9±23.6	5143.3±1630	2271.7±212	2448.3±1007	34.15±5	60.45±5.3	1.06±0.40	0.63±0.14	0.84±0.11	4.8±1.7	2.0±0. 7

^a^CEC = soil potential Cation Exchange Capacity; Nitr-N = Nitrate N; Amm-N = Ammonia N; Bio-P = Bioavailable Phosphorus; Mass = Litter mass; CStock = Litter C stock.

The sixth sentence of the second paragraph of the Results should read: While the concentration of the base cations Ca and Mg were similar between the study sites (7.4–137.5 ppm), K concentrations in the soil solution were more than doublein YOKO (31.3–68.9 ppm) compared to YGB (14.9–34.2 ppm), and Na concentrations were about 50–70% higher in YOKO (12.0–13.3 ppm) compared to YGB (7.6–7.8 ppm). Nitrate-N (9.68–1.98 mg kg^-1^), Ammonia-N (9.54–1.95 mg kg^-1^) and bioavailable P (3.3–8.9 mg kg^-1^), decreased with soil depth but showed no significant difference (p>0.05) in concentrations between both sites.

The fourth sentence of the third paragraph of the Results should read: While bioavailable P (0.80–0.84 mg kg^-1^) and Ammonia-N concentrations (0.68–0.63 mg kg^-1^) were similar for the litter of the different sites, Nitrate-N concentrations were about 86% higher at YOKO (1.98 mg kg^-1^) compared to YGB (1.06 mg kg^-1^).

## References

[1] Doetterl S, Kearsley E, Bauters M, Hufkens K, Lisingo J, Baert G, et al (2015) Aboveground vs. Belowground Carbon Stocks in African Tropical Lowland Rainforest: Drivers and Implications. PLoS ONE 10(11): e0143209 doi: 10.1371/journal.pone.0143209 26599231 10.1371/journal.pone.0143209 PMC4657968

