# Supplementary figures and images for: Aboveground vs. Belowground Carbon Stocks in African Tropical Lowland Rainforest: Drivers and Implications
Source: PLoS One. 2015 Nov 24;10(11):e0143209. doi: 10.1371/journal.pone.0143209 (PMC4657968; doi:10.1371/journal.pone.0143209)

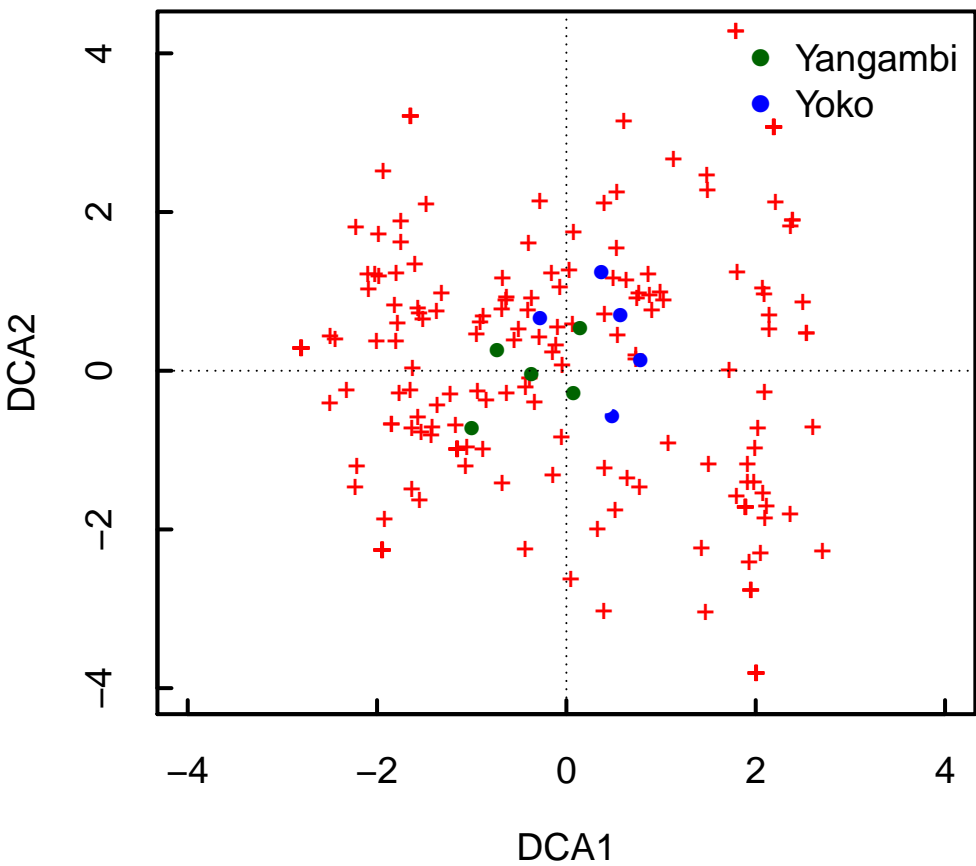

Supplement: S1 Fig — Based on plot scores of each 1 hectare plot (dots), the two sites, Yoko (blue dots) and Yangambi (green dots), show a similar species composition (ANOVA on DCA axis 1 and 2, p > 0.05 and p > 0.1 respectively). The spread of the species scores themselves (red crosses) indicate some variability within the plots. (PDF) [file pone.0143209.s001.pdf]

**Yangambi**

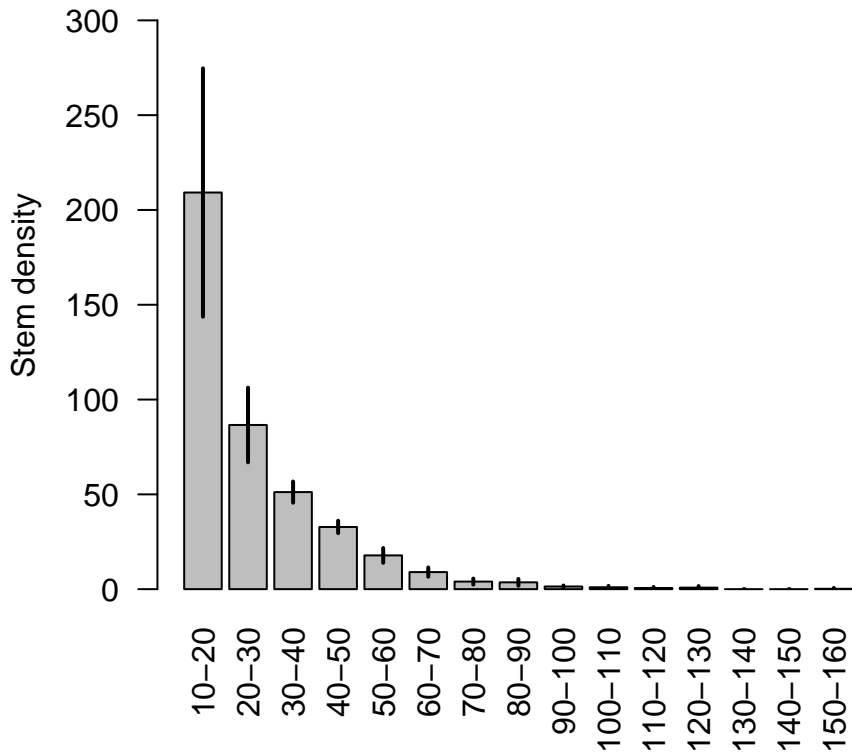

**Yoko**

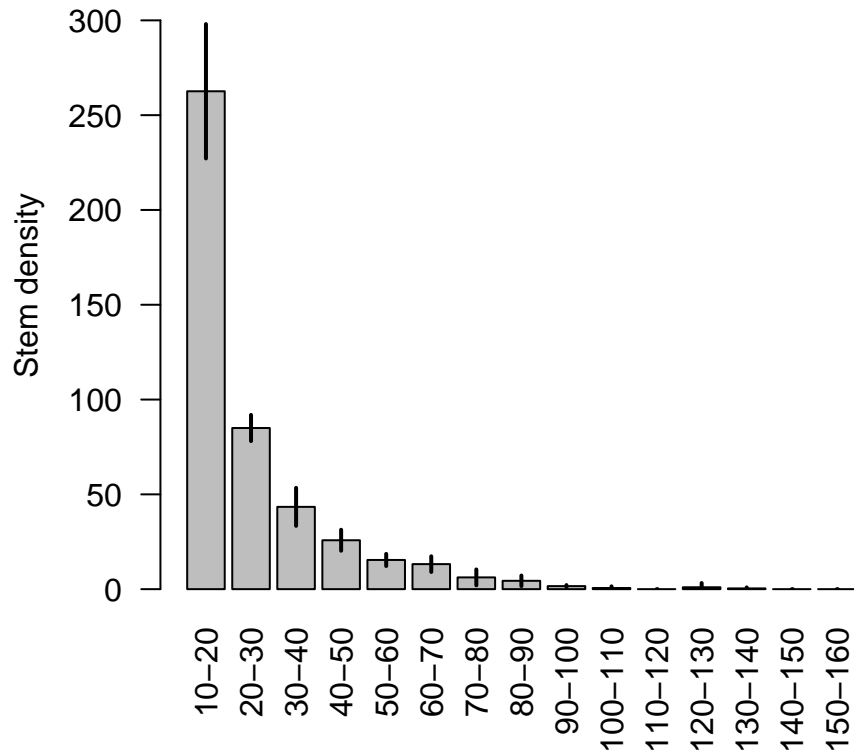

Supplement: S2 Fig — Bars represent mean of five hectares in both sites, with standard deviation indicated by line segments. (PDF) [file pone.0143209.s002.pdf]
