# Supplementary material for: Aboveground vs. Belowground Carbon Stocks in African Tropical Lowland Rainforest: Drivers and Implications
Source: PLoS One. 2015 Nov 24;10(11):e0143209. doi: 10.1371/journal.pone.0143209 (PMC4657968; doi:10.1371/journal.pone.0143209)
Supplement: S1 File — Includes figures, tables and references. (DOCX) [file pone.0143209.s003.docx]

**Aboveground vs. belowground carbon stocks in African tropical lowland rainforest: Drivers and implications**

Sebastian Doetterl*^1,6^, Elizabeth Kearsley*^1,5^, Marijn Bauters^1^, Koen Hufkens^2^, Janvier Lisingo^3^, Geert Baert^4^, Hans Verbeeck^5^, Pascal Boeckx^1^

1. Ghent University, Isotope Bioscience Laboratory - ISOFYS, Ghent, Belgium.
2. Harvard University, Department of Organismic and Evolutionary Biology, Cambridge, USA.
3. University of Kisangani, Faculty of Sciences, Kisangani, DR Congo.
4. Ghent University, Department of Applied Biosciences, Ghent, Belgium.
5. Ghent University, Computational & Applied Vegetation Ecology - CAVElab, Ghent, Belgium.
6. Augsburg University, Department of Geography, Augsburg, Germany.

* The contribution of these authors to the manuscript is considered equal.

**Corresponding author information**

**Email:** [**Sebastian.Doetterl@Ugent.be**](mailto:Sebastian.Doetterl@Ugent.be)

**Tel.** +32 9264 6006

**Fax.** +32 9264 6001

**Supplementary information**

**Forest structure and floristic composition**

At each site within 5 plots of 1 hectare, the forest stands were characterized by computing plot specific basal area, stem density, wood density (basal area weighted) and biodiversity indices. The number of species found within one hectare represents the species richness. Species diversity indices were described using the Shannon-Weaver [61] and Simpson ([62] indices, and Pielou’s evenness [63]. Evenness measures the similarity of the abundances of different species. The Shannon-Weaver index takes into account the evenness of the abundance of species, while Simpson’s index is less sensitive to the species richness but more sensitive to the most abundant species. All indices were calculated using the formulas provided in the package VEGAN 2.0 [64] in R 2.13.1 [65]. The floristic composition of the plots was compared with a detrended correspondence analysis (DCA) [66], providing geometric representations of stands and species wherein stands of similar composition, or species of similar distribution are found near each other. The analysis was performed using the VEGAN package and was based on the abundance of species in each plot. Student-t tests were done on the plot scores of the first two axes, to evaluate whether species composition differed between Yoko and Yangambi.

Both sites show similar species richness, Shannon-Weaver and Pielou’s Evenness indices, although Simpson’s index is slightly higher in Yoko (Table S1). Accordingly, the floristic comparison between plots at both sites as analysed using DCA did not show significant differences (Fig S1). Both for first and second axis of the DCA, no significant difference (p > 0.05 and p > 0.1) is found between the plot scores of Yoko and Yangambi indicating a similar species composition.


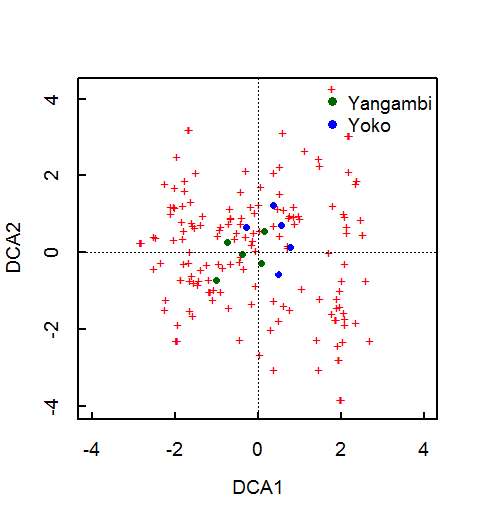


**S1 Fig.** Ordinations of species composition on the first two axis of a detrended correspondence analysis. Based on plot scores of each 1 hectare plot (dots), the two sites, Yoko (blue dots) and Yangambi (green dots), show a similar species composition (ANOVA on DCA axis 1 and 2, p > 0.05 and p > 0.1 respectively). The spread of the species scores themselves (red crosses) indicate some variability within the plots.

**S1 Table.** Stand characteristics (Aboveground carbon (AGC); Leaf area index (LAI)), species diversity and location of the two sites^a^.

|  |  | **Yangambi** | **YOKO** |
| --- | --- | --- | --- |
| **Stand characteristics** |  |  |  |
|  | Wood density (g cm^-3^) | 0.63 ± 0.02 (a) | 0.64 ± 0.02 (a) |
|  | Stem density (ha^-1^) | 419 ± 89 (a) | 469 ± 35 (a) |
|  | Basal area (m^2^ ha^-1^) | 32 ± 3 (a) | 34 ± 3 (a) |
|  | AGC (Mg C ha^-1^) | 163 ± 19 (a) | 191 ± 28 (b) |
|  | LAI | 3.9 ± 0.7 (a) | 4.1 ± 0.8 (a) |
|  | H-D model (H = a – b.e^-cD^) | a 36.358; b 31.659; c 0.022 | a 42.502; b 39.147; c 0.020 |
| **Species diversity** |  |  |  |
|  | Species richness | 76 ± 6 (a) | 78 ± 7 (a) |
|  | Pielou’s evenness | 0.83 ± 0.03 (a) | 0.85 ± 0.01 (a) |
|  | Shannon index | 3.6 ± 0.2 (a) | 3.7 ± 0.1 (a) |
|  | Simpson diversity | 0.955 ± 0.007 (a) | 0.962 ± 0.003 (b) |
| **Site location** |  |  |  |
|  | Latitude (d.dddd) | 0.7995 | 0.2918 |
|  | Longitude (d.dddd) | 24.5077 | 25.3113 |
|  | Altitude (m asl) | 479 ± 13 | 471 ± 5 |
|  | Plot size (ha) | 1 | 0.25 - 1 |

^a^Regression model: (H = a – b.e^-cD^). a, b and c are the optimized parameters for the individual equations per site, which represent, respectively, the maximum asymptotic height, the difference between minimum and maximum height, and shape of the curve [67]. For each parameter, significance from t-test is provided between brackets comparing the two sites. Values within a row not sharing a common letter differ significantly (p > 0.01).

Stem density of the two sites is similar. Additionally, the number of individual plants in the different DBH size classes shows that both localities have the typical inverted J curve where abundance decreases with increasing diameter (Fig S2). Additionally, a correspondence analysis of the abundance of plot x stem in multiple diameter classes (steps of 10 cm) did not reveal differences between the size structure of the trees at the two sites.


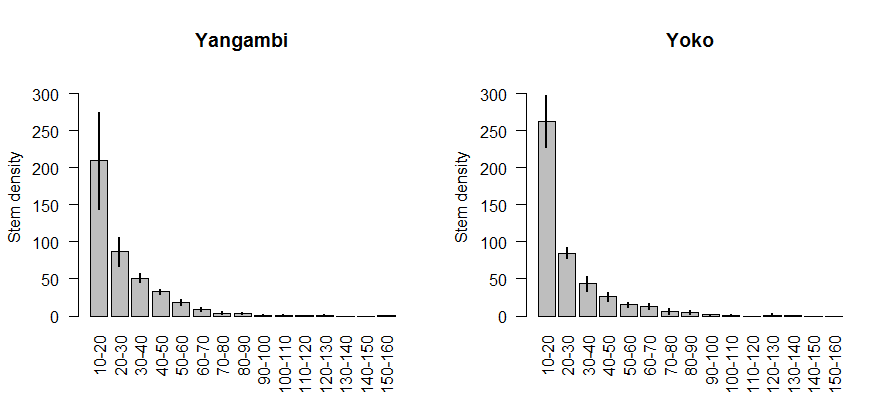


**S2 Fig.** Distribution of individuals trees in different diameter size classes. Bars represent mean of five hectares in both sites, with standard deviation indicated by line segments.

**S2 Table.** Average number of stems with standard deviation in brackets of tree species per hectare in Yoko within different diameter classes.

| **Species/diameter class** | **10-20** | **20-30** | **30-40** | **40-50** | **50-60** | **60-70** | **70-80** | **80-90** | **90-100** | **100-110** | **110-120** | **120-130** | **130-140** |
| --- | --- | --- | --- | --- | --- | --- | --- | --- | --- | --- | --- | --- | --- |
| Afrostyrax lepidophyllus | 0.7 ( 1.2 ) | 0.2 ( 0.4 ) | 0 ( 0 ) | 0.2 ( 0.4 ) | 0 ( 0 ) | 0 ( 0 ) | 0 ( 0 ) | 0 ( 0 ) | 0 ( 0 ) | 0 ( 0 ) | 0 ( 0 ) | 0 ( 0 ) | 0 ( 0 ) |
| Aidia micrantha | 18.5 ( 6.2 ) | 0.5 ( 0.8 ) | 0 ( 0 ) | 0 ( 0 ) | 0 ( 0 ) | 0 ( 0 ) | 0 ( 0 ) | 0 ( 0 ) | 0 ( 0 ) | 0 ( 0 ) | 0 ( 0 ) | 0 ( 0 ) | 0 ( 0 ) |
| Albizia adianthifolia | 1 ( 1.3 ) | 0.8 ( 0.8 ) | 0 ( 0 ) | 0.2 ( 0.4 ) | 0 ( 0 ) | 0 ( 0 ) | 0 ( 0 ) | 0 ( 0 ) | 0 ( 0 ) | 0 ( 0 ) | 0 ( 0 ) | 0 ( 0 ) | 0 ( 0 ) |
| Albizia ferruginea | 0 ( 0 ) | 0.2 ( 0.4 ) | 0 ( 0 ) | 0 ( 0 ) | 0 ( 0 ) | 0.2 ( 0.4 ) | 0 ( 0 ) | 0 ( 0 ) | 0 ( 0 ) | 0 ( 0 ) | 0 ( 0 ) | 0 ( 0 ) | 0 ( 0 ) |
| Allanblackia floribunda | 1.7 ( 1.6 ) | 0.5 ( 1.2 ) | 0.2 ( 0.4 ) | 0 ( 0 ) | 0 ( 0 ) | 0 ( 0 ) | 0 ( 0 ) | 0 ( 0 ) | 0 ( 0 ) | 0 ( 0 ) | 0 ( 0 ) | 0 ( 0 ) | 0 ( 0 ) |
| Alstonia boonei | 0 ( 0 ) | 0 ( 0 ) | 0 ( 0 ) | 0 ( 0 ) | 0 ( 0 ) | 0.2 ( 0.4 ) | 0 ( 0 ) | 0 ( 0 ) | 0 ( 0 ) | 0 ( 0 ) | 0 ( 0 ) | 0 ( 0 ) | 0 ( 0 ) |
| Anonidium mannii | 1.5 ( 1 ) | 2.7 ( 2.1 ) | 3.3 ( 0.5 ) | 2.7 ( 3 ) | 0.2 ( 0.4 ) | 0.3 ( 0.5 ) | 0 ( 0 ) | 0 ( 0 ) | 0 ( 0 ) | 0 ( 0 ) | 0 ( 0 ) | 0 ( 0 ) | 0 ( 0 ) |
| Anthonotha fragrans | 2.2 ( 2.4 ) | 0 ( 0 ) | 0 ( 0 ) | 0.2 ( 0.4 ) | 0.2 ( 0.4 ) | 0.3 ( 0.5 ) | 0 ( 0 ) | 0 ( 0 ) | 0.2 ( 0.4 ) | 0 ( 0 ) | 0 ( 0 ) | 0 ( 0 ) | 0 ( 0 ) |
| Anthonotha macrophylla | 0.5 ( 0.8 ) | 0 ( 0 ) | 0 ( 0 ) | 0 ( 0 ) | 0 ( 0 ) | 0 ( 0 ) | 0 ( 0 ) | 0 ( 0 ) | 0 ( 0 ) | 0 ( 0 ) | 0 ( 0 ) | 0 ( 0 ) | 0 ( 0 ) |
| Antrocaryon nannanii | 0 ( 0 ) | 0.2 ( 0.4 ) | 0 ( 0 ) | 0 ( 0 ) | 0 ( 0 ) | 0 ( 0 ) | 0 ( 0 ) | 0 ( 0 ) | 0 ( 0 ) | 0.2 ( 0.4 ) | 0 ( 0 ) | 0 ( 0 ) | 0 ( 0 ) |
| Balanites wilsoniana | 0.2 ( 0.4 ) | 0 ( 0 ) | 0 ( 0 ) | 0 ( 0 ) | 0 ( 0 ) | 0 ( 0 ) | 0 ( 0 ) | 0 ( 0 ) | 0 ( 0 ) | 0 ( 0 ) | 0 ( 0 ) | 0 ( 0 ) | 0 ( 0 ) |
| Baphia dewevrei | 0.2 ( 0.4 ) | 0.2 ( 0.4 ) | 0 ( 0 ) | 0 ( 0 ) | 0 ( 0 ) | 0 ( 0 ) | 0 ( 0 ) | 0 ( 0 ) | 0 ( 0 ) | 0 ( 0 ) | 0 ( 0 ) | 0 ( 0 ) | 0 ( 0 ) |
| Barteria fistulosa | 0.5 ( 0.8 ) | 0 ( 0 ) | 0 ( 0 ) | 0 ( 0 ) | 0 ( 0 ) | 0 ( 0 ) | 0 ( 0 ) | 0 ( 0 ) | 0 ( 0 ) | 0 ( 0 ) | 0 ( 0 ) | 0 ( 0 ) | 0 ( 0 ) |
| Berlinia congolensis | 0 ( 0 ) | 0 ( 0 ) | 0.2 ( 0.4 ) | 0 ( 0 ) | 0 ( 0 ) | 0 ( 0 ) | 0 ( 0 ) | 0 ( 0 ) | 0 ( 0 ) | 0 ( 0 ) | 0 ( 0 ) | 0 ( 0 ) | 0 ( 0 ) |
| Blighia unijugata | 0.8 ( 0.8 ) | 0 ( 0 ) | 0 ( 0 ) | 0 ( 0 ) | 0 ( 0 ) | 0 ( 0 ) | 0 ( 0 ) | 0 ( 0 ) | 0 ( 0 ) | 0 ( 0 ) | 0 ( 0 ) | 0 ( 0 ) | 0 ( 0 ) |
| Blighia welwitschii | 0.2 ( 0.4 ) | 0 ( 0 ) | 0.2 ( 0.4 ) | 0.2 ( 0.4 ) | 0 ( 0 ) | 0 ( 0 ) | 0 ( 0 ) | 0 ( 0 ) | 0 ( 0 ) | 0.2 ( 0.4 ) | 0 ( 0 ) | 0 ( 0 ) | 0 ( 0 ) |
| Bosqueia angolensis | 1 ( 1.3 ) | 0.7 ( 1.2 ) | 0.5 ( 0.8 ) | 0 ( 0 ) | 0 ( 0 ) | 0 ( 0 ) | 0 ( 0 ) | 0 ( 0 ) | 0 ( 0 ) | 0 ( 0 ) | 0 ( 0 ) | 0 ( 0 ) | 0 ( 0 ) |
| Breviea sericea | 0.3 ( 0.5 ) | 0 ( 0 ) | 0 ( 0 ) | 0 ( 0 ) | 0 ( 0 ) | 0 ( 0 ) | 0 ( 0 ) | 0 ( 0 ) | 0 ( 0 ) | 0 ( 0 ) | 0 ( 0 ) | 0 ( 0 ) | 0 ( 0 ) |
| Breviea sp. | 0 ( 0 ) | 0.2 ( 0.4 ) | 0 ( 0 ) | 0 ( 0 ) | 0 ( 0 ) | 0 ( 0 ) | 0 ( 0 ) | 0 ( 0 ) | 0 ( 0 ) | 0 ( 0 ) | 0 ( 0 ) | 0 ( 0 ) | 0 ( 0 ) |
| Canarium schweinfurthii | 0 ( 0 ) | 0 ( 0 ) | 0.2 ( 0.4 ) | 0 ( 0 ) | 0 ( 0 ) | 0 ( 0 ) | 0 ( 0 ) | 0 ( 0 ) | 0.2 ( 0.4 ) | 0 ( 0 ) | 0 ( 0 ) | 0 ( 0 ) | 0 ( 0 ) |
| Carapa procera | 6.7 ( 4.9 ) | 0.5 ( 0.8 ) | 0.2 ( 0.4 ) | 0 ( 0 ) | 0 ( 0 ) | 0 ( 0 ) | 0 ( 0 ) | 0 ( 0 ) | 0 ( 0 ) | 0 ( 0 ) | 0 ( 0 ) | 0 ( 0 ) | 0 ( 0 ) |
| Celtis mildbraedii | 2.3 ( 2.1 ) | 0.7 ( 1.2 ) | 1 ( 0.9 ) | 0.5 ( 0.5 ) | 1 ( 0.9 ) | 0.7 ( 0.8 ) | 0.3 ( 0.5 ) | 0.2 ( 0.4 ) | 0 ( 0 ) | 0 ( 0 ) | 0.2 ( 0.4 ) | 0 ( 0 ) | 0 ( 0 ) |
| Celtis tessmannii | 0.2 ( 0.4 ) | 0 ( 0 ) | 0 ( 0 ) | 0.2 ( 0.4 ) | 0 ( 0 ) | 0.2 ( 0.4 ) | 0 ( 0 ) | 0 ( 0 ) | 0 ( 0 ) | 0 ( 0 ) | 0 ( 0 ) | 0 ( 0 ) | 0 ( 0 ) |
| Chlamydocola chlamydantha | 0.2 ( 0.4 ) | 0 ( 0 ) | 0 ( 0 ) | 0 ( 0 ) | 0 ( 0 ) | 0 ( 0 ) | 0 ( 0 ) | 0 ( 0 ) | 0 ( 0 ) | 0 ( 0 ) | 0 ( 0 ) | 0 ( 0 ) | 0 ( 0 ) |
| Chrysophyllum africanum | 1.5 ( 1.6 ) | 0 ( 0 ) | 0.2 ( 0.4 ) | 0.2 ( 0.4 ) | 0 ( 0 ) | 0 ( 0 ) | 0 ( 0 ) | 0 ( 0 ) | 0 ( 0 ) | 0 ( 0 ) | 0 ( 0 ) | 0 ( 0 ) | 0 ( 0 ) |
| Chrysophyllum lacourtianum | 2 ( 2.6 ) | 0.2 ( 0.4 ) | 0.2 ( 0.4 ) | 0 ( 0 ) | 0 ( 0 ) | 0 ( 0 ) | 0 ( 0 ) | 0 ( 0 ) | 0 ( 0 ) | 0 ( 0 ) | 0 ( 0 ) | 0 ( 0 ) | 0 ( 0 ) |
| Chrysophyllum pruniforme | 0.5 ( 0.5 ) | 0.2 ( 0.4 ) | 0.2 ( 0.4 ) | 0 ( 0 ) | 0 ( 0 ) | 0.2 ( 0.4 ) | 0 ( 0 ) | 0.2 ( 0.4 ) | 0 ( 0 ) | 0 ( 0 ) | 0 ( 0 ) | 0 ( 0 ) | 0 ( 0 ) |
| Chytranthus carneus | 1.7 ( 1 ) | 0.2 ( 0.4 ) | 0 ( 0 ) | 0 ( 0 ) | 0 ( 0 ) | 0 ( 0 ) | 0 ( 0 ) | 0 ( 0 ) | 0 ( 0 ) | 0 ( 0 ) | 0 ( 0 ) | 0 ( 0 ) | 0 ( 0 ) |
| Cleistanthus mildbraedii | 3.3 ( 1.6 ) | 0.7 ( 0.8 ) | 0 ( 0 ) | 0.5 ( 0.8 ) | 0.2 ( 0.4 ) | 0.2 ( 0.4 ) | 0 ( 0 ) | 0 ( 0 ) | 0 ( 0 ) | 0 ( 0 ) | 0 ( 0 ) | 0 ( 0 ) | 0 ( 0 ) |
| Cleistanthus ripicola | 0.5 ( 1.2 ) | 0.5 ( 1.2 ) | 0.2 ( 0.4 ) | 0 ( 0 ) | 0 ( 0 ) | 0.2 ( 0.4 ) | 0 ( 0 ) | 0 ( 0 ) | 0 ( 0 ) | 0 ( 0 ) | 0 ( 0 ) | 0 ( 0 ) | 0 ( 0 ) |
| Coelocaryon botryoides | 2.3 ( 2 ) | 1 ( 0.9 ) | 0.3 ( 0.5 ) | 0 ( 0 ) | 0 ( 0 ) | 0 ( 0 ) | 0 ( 0 ) | 0 ( 0 ) | 0 ( 0 ) | 0 ( 0 ) | 0 ( 0 ) | 0 ( 0 ) | 0 ( 0 ) |
| Cola acuminata | 0.3 ( 0.8 ) | 0 ( 0 ) | 0 ( 0 ) | 0 ( 0 ) | 0 ( 0 ) | 0 ( 0 ) | 0 ( 0 ) | 0 ( 0 ) | 0 ( 0 ) | 0 ( 0 ) | 0 ( 0 ) | 0 ( 0 ) | 0 ( 0 ) |
| Cola gigantea | 0.8 ( 1 ) | 0 ( 0 ) | 0 ( 0 ) | 0 ( 0 ) | 0 ( 0 ) | 0 ( 0 ) | 0 ( 0 ) | 0 ( 0 ) | 0 ( 0 ) | 0 ( 0 ) | 0 ( 0 ) | 0 ( 0 ) | 0 ( 0 ) |
| Cola griseiflora | 23.8 ( 10.6 ) | 1 ( 0.9 ) | 0.3 ( 0.5 ) | 0 ( 0 ) | 0 ( 0 ) | 0 ( 0 ) | 0 ( 0 ) | 0 ( 0 ) | 0 ( 0 ) | 0 ( 0 ) | 0 ( 0 ) | 0 ( 0 ) | 0 ( 0 ) |
| Combretum lokele | 0 ( 0 ) | 0 ( 0 ) | 0 ( 0 ) | 0 ( 0 ) | 0 ( 0 ) | 0 ( 0 ) | 0 ( 0 ) | 0 ( 0 ) | 0.2 ( 0.4 ) | 0 ( 0 ) | 0 ( 0 ) | 0 ( 0 ) | 0 ( 0 ) |
| Copaifera mildbraedii | 0.5 ( 0.5 ) | 0 ( 0 ) | 0 ( 0 ) | 0 ( 0 ) | 0 ( 0 ) | 0 ( 0 ) | 0 ( 0 ) | 0 ( 0 ) | 0 ( 0 ) | 0 ( 0 ) | 0 ( 0 ) | 0 ( 0 ) | 0 ( 0 ) |
| Cynometra hankei | 1.8 ( 1.2 ) | 1.7 ( 1.6 ) | 1.3 ( 0.8 ) | 1.3 ( 1.5 ) | 0.7 ( 1.2 ) | 1.2 ( 1.2 ) | 1 ( 1.7 ) | 0.8 ( 1 ) | 0.2 ( 0.4 ) | 0 ( 0 ) | 0 ( 0 ) | 0.2 ( 0.4 ) | 0 ( 0 ) |
| Cynometra sessiliflora | 0 ( 0 ) | 0.2 ( 0.4 ) | 0 ( 0 ) | 0 ( 0 ) | 0 ( 0 ) | 0 ( 0 ) | 0 ( 0 ) | 0 ( 0 ) | 0 ( 0 ) | 0 ( 0 ) | 0 ( 0 ) | 0 ( 0 ) | 0 ( 0 ) |
| Dacryodes edulis | 0.5 ( 0.5 ) | 0 ( 0 ) | 0.2 ( 0.4 ) | 0 ( 0 ) | 0 ( 0 ) | 0 ( 0 ) | 0 ( 0 ) | 0 ( 0 ) | 0 ( 0 ) | 0 ( 0 ) | 0 ( 0 ) | 0 ( 0 ) | 0 ( 0 ) |
| Dacryodes osika | 0 ( 0 ) | 0.3 ( 0.8 ) | 0 ( 0 ) | 0 ( 0 ) | 0 ( 0 ) | 0 ( 0 ) | 0 ( 0 ) | 0 ( 0 ) | 0 ( 0 ) | 0 ( 0 ) | 0 ( 0 ) | 0 ( 0 ) | 0 ( 0 ) |
| Desplatsia dewevrei | 0.2 ( 0.4 ) | 0 ( 0 ) | 0 ( 0 ) | 0 ( 0 ) | 0 ( 0 ) | 0 ( 0 ) | 0 ( 0 ) | 0 ( 0 ) | 0 ( 0 ) | 0 ( 0 ) | 0 ( 0 ) | 0 ( 0 ) | 0 ( 0 ) |
| Dialium corbisieri | 0.5 ( 0.5 ) | 0.3 ( 0.5 ) | 0 ( 0 ) | 0 ( 0 ) | 0.3 ( 0.5 ) | 0.2 ( 0.4 ) | 0 ( 0 ) | 0 ( 0 ) | 0 ( 0 ) | 0 ( 0 ) | 0 ( 0 ) | 0 ( 0 ) | 0 ( 0 ) |
| Dialium excelsum | 0 ( 0 ) | 0 ( 0 ) | 0 ( 0 ) | 0 ( 0 ) | 0 ( 0 ) | 0 ( 0 ) | 0.2 ( 0.4 ) | 0 ( 0 ) | 0 ( 0 ) | 0 ( 0 ) | 0 ( 0 ) | 0 ( 0 ) | 0 ( 0 ) |
| Dialium pachyphyllum | 2.5 ( 2.7 ) | 0.2 ( 0.4 ) | 0 ( 0 ) | 0 ( 0 ) | 0 ( 0 ) | 0 ( 0 ) | 0 ( 0 ) | 0.2 ( 0.4 ) | 0 ( 0 ) | 0 ( 0 ) | 0 ( 0 ) | 0 ( 0 ) | 0 ( 0 ) |
| Dialium soyauxii | 0 ( 0 ) | 0 ( 0 ) | 0 ( 0 ) | 0.2 ( 0.4 ) | 0 ( 0 ) | 0 ( 0 ) | 0 ( 0 ) | 0 ( 0 ) | 0 ( 0 ) | 0 ( 0 ) | 0 ( 0 ) | 0 ( 0 ) | 0 ( 0 ) |
| Diogoa zenkeri | 4.3 ( 3.3 ) | 2.5 ( 2.4 ) | 1 ( 0.9 ) | 0.2 ( 0.4 ) | 0 ( 0 ) | 0 ( 0 ) | 0 ( 0 ) | 0 ( 0 ) | 0 ( 0 ) | 0 ( 0 ) | 0 ( 0 ) | 0 ( 0 ) | 0 ( 0 ) |
| Diospyros boala | 0.3 ( 0.8 ) | 0 ( 0 ) | 0 ( 0 ) | 0 ( 0 ) | 0 ( 0 ) | 0 ( 0 ) | 0 ( 0 ) | 0 ( 0 ) | 0 ( 0 ) | 0 ( 0 ) | 0 ( 0 ) | 0 ( 0 ) | 0 ( 0 ) |
| Diospyros crassiflora | 0.2 ( 0.4 ) | 0 ( 0 ) | 0 ( 0 ) | 0 ( 0 ) | 0 ( 0 ) | 0 ( 0 ) | 0 ( 0 ) | 0 ( 0 ) | 0 ( 0 ) | 0 ( 0 ) | 0 ( 0 ) | 0 ( 0 ) | 0 ( 0 ) |
| Diospyros sp. | 1.5 ( 1.9 ) | 0.5 ( 0.5 ) | 0 ( 0 ) | 0 ( 0 ) | 0 ( 0 ) | 0 ( 0 ) | 0 ( 0 ) | 0 ( 0 ) | 0 ( 0 ) | 0 ( 0 ) | 0 ( 0 ) | 0 ( 0 ) | 0 ( 0 ) |
| Drypetes gossweileri | 1.3 ( 2 ) | 0.7 ( 0.8 ) | 0.2 ( 0.4 ) | 0 ( 0 ) | 0 ( 0 ) | 0 ( 0 ) | 0 ( 0 ) | 0 ( 0 ) | 0 ( 0 ) | 0 ( 0 ) | 0 ( 0 ) | 0 ( 0 ) | 0 ( 0 ) |
| Drypetes likwa | 2 ( 4.9 ) | 0.8 ( 2 ) | 0.7 ( 1.6 ) | 0.2 ( 0.4 ) | 0.2 ( 0.4 ) | 0 ( 0 ) | 0 ( 0 ) | 0 ( 0 ) | 0 ( 0 ) | 0 ( 0 ) | 0 ( 0 ) | 0 ( 0 ) | 0 ( 0 ) |
| Drypetes sp. | 8.3 ( 6.4 ) | 3.3 ( 3 ) | 0.8 ( 1.3 ) | 0.2 ( 0.4 ) | 0 ( 0 ) | 0 ( 0 ) | 0 ( 0 ) | 0 ( 0 ) | 0 ( 0 ) | 0 ( 0 ) | 0 ( 0 ) | 0 ( 0 ) | 0 ( 0 ) |
| Drypetes spinosodentata | 0.5 ( 1.2 ) | 0.2 ( 0.4 ) | 0 ( 0 ) | 0 ( 0 ) | 0 ( 0 ) | 0 ( 0 ) | 0 ( 0 ) | 0 ( 0 ) | 0 ( 0 ) | 0 ( 0 ) | 0 ( 0 ) | 0 ( 0 ) | 0 ( 0 ) |
| Entandrophragma angolense | 0 ( 0 ) | 0.2 ( 0.4 ) | 0 ( 0 ) | 0 ( 0 ) | 0 ( 0 ) | 0 ( 0 ) | 0 ( 0 ) | 0 ( 0 ) | 0 ( 0 ) | 0 ( 0 ) | 0 ( 0 ) | 0 ( 0 ) | 0 ( 0 ) |
| Entandrophragma candollei | 0.3 ( 0.5 ) | 0.2 ( 0.4 ) | 0 ( 0 ) | 0 ( 0 ) | 0.2 ( 0.4 ) | 0 ( 0 ) | 0 ( 0 ) | 0 ( 0 ) | 0 ( 0 ) | 0 ( 0 ) | 0 ( 0 ) | 0 ( 0 ) | 0.2 ( 0.4 ) |
| Entandrophragma cylindricum | 0.5 ( 0.8 ) | 0 ( 0 ) | 0 ( 0 ) | 0 ( 0 ) | 0 ( 0 ) | 0 ( 0 ) | 0 ( 0 ) | 0 ( 0 ) | 0 ( 0 ) | 0 ( 0 ) | 0 ( 0 ) | 0 ( 0 ) | 0 ( 0 ) |
| Entandrophragma utile | 0.2 ( 0.4 ) | 0 ( 0 ) | 0 ( 0 ) | 0 ( 0 ) | 0.2 ( 0.4 ) | 0 ( 0 ) | 0 ( 0 ) | 0 ( 0 ) | 0 ( 0 ) | 0 ( 0 ) | 0 ( 0 ) | 0 ( 0 ) | 0 ( 0 ) |
| Eriocoelum microspermum | 0.2 ( 0.4 ) | 0 ( 0 ) | 0 ( 0 ) | 0 ( 0 ) | 0 ( 0 ) | 0 ( 0 ) | 0 ( 0 ) | 0 ( 0 ) | 0 ( 0 ) | 0 ( 0 ) | 0 ( 0 ) | 0 ( 0 ) | 0 ( 0 ) |
| Ficus elastica | 0 ( 0 ) | 0 ( 0 ) | 0 ( 0 ) | 0 ( 0 ) | 0 ( 0 ) | 0.2 ( 0.4 ) | 0 ( 0 ) | 0 ( 0 ) | 0 ( 0 ) | 0 ( 0 ) | 0 ( 0 ) | 0 ( 0 ) | 0 ( 0 ) |
| Funtumia africana | 1.8 ( 1.9 ) | 0.5 ( 0.8 ) | 0 ( 0 ) | 0.2 ( 0.4 ) | 0 ( 0 ) | 0 ( 0 ) | 0 ( 0 ) | 0 ( 0 ) | 0 ( 0 ) | 0 ( 0 ) | 0 ( 0 ) | 0 ( 0 ) | 0 ( 0 ) |
| Garcinia epunctata | 0.5 ( 0.8 ) | 0 ( 0 ) | 0 ( 0 ) | 0 ( 0 ) | 0 ( 0 ) | 0 ( 0 ) | 0 ( 0 ) | 0 ( 0 ) | 0 ( 0 ) | 0 ( 0 ) | 0 ( 0 ) | 0 ( 0 ) | 0 ( 0 ) |
| Garcinia punctata | 0.3 ( 0.5 ) | 0.2 ( 0.4 ) | 0 ( 0 ) | 0 ( 0 ) | 0 ( 0 ) | 0 ( 0 ) | 0 ( 0 ) | 0 ( 0 ) | 0 ( 0 ) | 0 ( 0 ) | 0 ( 0 ) | 0 ( 0 ) | 0 ( 0 ) |
| Garcinia smeathmannii | 0.5 ( 0.8 ) | 0 ( 0 ) | 0 ( 0 ) | 0 ( 0 ) | 0 ( 0 ) | 0 ( 0 ) | 0 ( 0 ) | 0 ( 0 ) | 0 ( 0 ) | 0 ( 0 ) | 0 ( 0 ) | 0 ( 0 ) | 0 ( 0 ) |
| Garcinia sp. | 0.2 ( 0.4 ) | 0 ( 0 ) | 0 ( 0 ) | 0 ( 0 ) | 0 ( 0 ) | 0 ( 0 ) | 0 ( 0 ) | 0 ( 0 ) | 0 ( 0 ) | 0 ( 0 ) | 0 ( 0 ) | 0 ( 0 ) | 0 ( 0 ) |
| Grewia oligoneura | 0.3 ( 0.5 ) | 0.2 ( 0.4 ) | 0.3 ( 0.5 ) | 0 ( 0 ) | 0 ( 0 ) | 0 ( 0 ) | 0 ( 0 ) | 0 ( 0 ) | 0 ( 0 ) | 0 ( 0 ) | 0 ( 0 ) | 0 ( 0 ) | 0 ( 0 ) |
| Grewia trinervia | 1.5 ( 0.8 ) | 0.5 ( 0.5 ) | 0.3 ( 0.5 ) | 0.3 ( 0.5 ) | 0 ( 0 ) | 0 ( 0 ) | 0 ( 0 ) | 0 ( 0 ) | 0 ( 0 ) | 0 ( 0 ) | 0 ( 0 ) | 0 ( 0 ) | 0 ( 0 ) |
| Grossera multinervis | 2 ( 4.9 ) | 0.8 ( 2 ) | 0.5 ( 1.2 ) | 0 ( 0 ) | 0 ( 0 ) | 0 ( 0 ) | 0 ( 0 ) | 0 ( 0 ) | 0 ( 0 ) | 0 ( 0 ) | 0 ( 0 ) | 0 ( 0 ) | 0 ( 0 ) |
| Guarea cedrata | 0.7 ( 0.8 ) | 0.2 ( 0.4 ) | 0 ( 0 ) | 0 ( 0 ) | 0 ( 0 ) | 0.2 ( 0.4 ) | 0.2 ( 0.4 ) | 0 ( 0 ) | 0.2 ( 0.4 ) | 0 ( 0 ) | 0 ( 0 ) | 0 ( 0 ) | 0 ( 0 ) |
| Guarea laurentii | 1.7 ( 2 ) | 1.5 ( 2.3 ) | 0.2 ( 0.4 ) | 0.2 ( 0.4 ) | 0 ( 0 ) | 0 ( 0 ) | 0 ( 0 ) | 0 ( 0 ) | 0 ( 0 ) | 0 ( 0 ) | 0 ( 0 ) | 0 ( 0 ) | 0 ( 0 ) |
| Guarea thompsonii | 10.2 ( 5.2 ) | 2.8 ( 1.7 ) | 0.3 ( 0.5 ) | 0.2 ( 0.4 ) | 0.2 ( 0.4 ) | 0.3 ( 0.5 ) | 0 ( 0 ) | 0 ( 0 ) | 0 ( 0 ) | 0.2 ( 0.4 ) | 0 ( 0 ) | 0 ( 0 ) | 0 ( 0 ) |
| Hannoa klaineana | 0.5 ( 0.5 ) | 0.2 ( 0.4 ) | 0 ( 0 ) | 0 ( 0 ) | 0 ( 0 ) | 0 ( 0 ) | 0 ( 0 ) | 0 ( 0 ) | 0 ( 0 ) | 0 ( 0 ) | 0 ( 0 ) | 0 ( 0 ) | 0 ( 0 ) |
| Heisteria parvifolia | 1.3 ( 0.8 ) | 1.3 ( 1 ) | 0.2 ( 0.4 ) | 0 ( 0 ) | 0 ( 0 ) | 0 ( 0 ) | 0 ( 0 ) | 0 ( 0 ) | 0 ( 0 ) | 0 ( 0 ) | 0 ( 0 ) | 0 ( 0 ) | 0 ( 0 ) |
| Hexalobus crispiflorus | 0.2 ( 0.4 ) | 0 ( 0 ) | 0 ( 0 ) | 0 ( 0 ) | 0.2 ( 0.4 ) | 0 ( 0 ) | 0 ( 0 ) | 0 ( 0 ) | 0 ( 0 ) | 0 ( 0 ) | 0 ( 0 ) | 0 ( 0 ) | 0 ( 0 ) |
| Homalium longistylum | 0.2 ( 0.4 ) | 0 ( 0 ) | 0 ( 0 ) | 0 ( 0 ) | 0 ( 0 ) | 0 ( 0 ) | 0 ( 0 ) | 0 ( 0 ) | 0 ( 0 ) | 0 ( 0 ) | 0 ( 0 ) | 0 ( 0 ) | 0 ( 0 ) |
| Homalium sp. | 0.2 ( 0.4 ) | 0.2 ( 0.4 ) | 0 ( 0 ) | 0 ( 0 ) | 0 ( 0 ) | 0 ( 0 ) | 0 ( 0 ) | 0 ( 0 ) | 0 ( 0 ) | 0 ( 0 ) | 0 ( 0 ) | 0 ( 0 ) | 0 ( 0 ) |
| Hymenostegia pellegrinii | 0 ( 0 ) | 0 ( 0 ) | 0 ( 0 ) | 0 ( 0 ) | 0.2 ( 0.4 ) | 0 ( 0 ) | 0 ( 0 ) | 0 ( 0 ) | 0 ( 0 ) | 0 ( 0 ) | 0 ( 0 ) | 0 ( 0 ) | 0 ( 0 ) |
| Irvingia gabonensis | 0.8 ( 1.2 ) | 0 ( 0 ) | 0 ( 0 ) | 0 ( 0 ) | 0 ( 0 ) | 0 ( 0 ) | 0 ( 0 ) | 0 ( 0 ) | 0 ( 0 ) | 0 ( 0 ) | 0 ( 0 ) | 0 ( 0 ) | 0 ( 0 ) |
| Irvingia grandifolia | 0.2 ( 0.4 ) | 0 ( 0 ) | 0 ( 0 ) | 0 ( 0 ) | 0 ( 0 ) | 0 ( 0 ) | 0 ( 0 ) | 0 ( 0 ) | 0 ( 0 ) | 0 ( 0 ) | 0 ( 0 ) | 0 ( 0 ) | 0 ( 0 ) |
| Julbernardia seretii | 3.5 ( 2.3 ) | 3 ( 1.3 ) | 3.2 ( 1.6 ) | 2.2 ( 1.8 ) | 1 ( 1.5 ) | 0.3 ( 0.5 ) | 0.2 ( 0.4 ) | 0 ( 0 ) | 0 ( 0 ) | 0 ( 0 ) | 0.2 ( 0.4 ) | 0 ( 0 ) | 0 ( 0 ) |
| Macaranga monandra | 0.8 ( 1.3 ) | 0.7 ( 1.2 ) | 0.3 ( 0.5 ) | 0.3 ( 0.8 ) | 0.2 ( 0.4 ) | 0 ( 0 ) | 0 ( 0 ) | 0 ( 0 ) | 0 ( 0 ) | 0 ( 0 ) | 0 ( 0 ) | 0 ( 0 ) | 0 ( 0 ) |
| Mammea africana | 0.2 ( 0.4 ) | 0 ( 0 ) | 0 ( 0 ) | 0 ( 0 ) | 0 ( 0 ) | 0 ( 0 ) | 0 ( 0 ) | 0 ( 0 ) | 0 ( 0 ) | 0 ( 0 ) | 0 ( 0 ) | 0 ( 0 ) | 0 ( 0 ) |
| Manilkara malcolens | 0 ( 0 ) | 0.2 ( 0.4 ) | 0 ( 0 ) | 0 ( 0 ) | 0 ( 0 ) | 0 ( 0 ) | 0 ( 0 ) | 0 ( 0 ) | 0 ( 0 ) | 0 ( 0 ) | 0 ( 0 ) | 0 ( 0 ) | 0 ( 0 ) |
| Manilkara sp. | 0.5 ( 0.8 ) | 0.2 ( 0.4 ) | 0 ( 0 ) | 0 ( 0 ) | 0 ( 0 ) | 0 ( 0 ) | 0 ( 0 ) | 0 ( 0 ) | 0 ( 0 ) | 0 ( 0 ) | 0 ( 0 ) | 0 ( 0 ) | 0 ( 0 ) |
| Margaritaria discoidea | 0.2 ( 0.4 ) | 0 ( 0 ) | 0 ( 0 ) | 0 ( 0 ) | 0 ( 0 ) | 0 ( 0 ) | 0 ( 0 ) | 0 ( 0 ) | 0 ( 0 ) | 0 ( 0 ) | 0 ( 0 ) | 0 ( 0 ) | 0 ( 0 ) |
| Massularia acuminata | 0.7 ( 0.8 ) | 0 ( 0 ) | 0 ( 0 ) | 0 ( 0 ) | 0 ( 0 ) | 0 ( 0 ) | 0 ( 0 ) | 0 ( 0 ) | 0 ( 0 ) | 0 ( 0 ) | 0 ( 0 ) | 0 ( 0 ) | 0 ( 0 ) |
| Massularia africana | 0.2 ( 0.4 ) | 0 ( 0 ) | 0 ( 0 ) | 0 ( 0 ) | 0 ( 0 ) | 0 ( 0 ) | 0 ( 0 ) | 0 ( 0 ) | 0 ( 0 ) | 0 ( 0 ) | 0 ( 0 ) | 0 ( 0 ) | 0 ( 0 ) |
| Microdesmis yafungana | 19.5 ( 9.4 ) | 5.7 ( 3.7 ) | 1.2 ( 1.6 ) | 0.5 ( 0.5 ) | 0 ( 0 ) | 0 ( 0 ) | 0 ( 0 ) | 0 ( 0 ) | 0 ( 0 ) | 0 ( 0 ) | 0 ( 0 ) | 0 ( 0 ) | 0 ( 0 ) |
| Millettia drastica | 0.3 ( 0.5 ) | 0 ( 0 ) | 0 ( 0 ) | 0 ( 0 ) | 0 ( 0 ) | 0 ( 0 ) | 0 ( 0 ) | 0 ( 0 ) | 0 ( 0 ) | 0 ( 0 ) | 0 ( 0 ) | 0 ( 0 ) | 0 ( 0 ) |
| Monodora angolensis | 0.8 ( 0.8 ) | 0 ( 0 ) | 0 ( 0 ) | 0 ( 0 ) | 0 ( 0 ) | 0 ( 0 ) | 0 ( 0 ) | 0 ( 0 ) | 0 ( 0 ) | 0 ( 0 ) | 0 ( 0 ) | 0 ( 0 ) | 0 ( 0 ) |
| Morinda lucida | 0.2 ( 0.4 ) | 0 ( 0 ) | 0 ( 0 ) | 0 ( 0 ) | 0 ( 0 ) | 0 ( 0 ) | 0 ( 0 ) | 0 ( 0 ) | 0 ( 0 ) | 0 ( 0 ) | 0 ( 0 ) | 0 ( 0 ) | 0 ( 0 ) |
| Musanga cecropioides | 0.5 ( 0.5 ) | 0.5 ( 0.5 ) | 0.3 ( 0.5 ) | 0.3 ( 0.5 ) | 0.2 ( 0.4 ) | 0 ( 0 ) | 0 ( 0 ) | 0 ( 0 ) | 0 ( 0 ) | 0 ( 0 ) | 0 ( 0 ) | 0 ( 0 ) | 0 ( 0 ) |
| Nesogordonia leplaei | 0.5 ( 1.2 ) | 0 ( 0 ) | 0 ( 0 ) | 0 ( 0 ) | 0 ( 0 ) | 0 ( 0 ) | 0 ( 0 ) | 0 ( 0 ) | 0 ( 0 ) | 0 ( 0 ) | 0 ( 0 ) | 0 ( 0 ) | 0 ( 0 ) |
| Nesogordonia sp. | 0.5 ( 0.8 ) | 0.5 ( 0.5 ) | 0.2 ( 0.4 ) | 0.3 ( 0.5 ) | 0.2 ( 0.4 ) | 0 ( 0 ) | 0 ( 0 ) | 0 ( 0 ) | 0 ( 0 ) | 0 ( 0 ) | 0 ( 0 ) | 0 ( 0 ) | 0 ( 0 ) |
| Omphalocarpum sp. | 0.3 ( 0.8 ) | 0.2 ( 0.4 ) | 0 ( 0 ) | 0 ( 0 ) | 0 ( 0 ) | 0 ( 0 ) | 0 ( 0 ) | 0 ( 0 ) | 0 ( 0 ) | 0 ( 0 ) | 0 ( 0 ) | 0 ( 0 ) | 0 ( 0 ) |
| Oncoba welwitschii | 0.5 ( 0.8 ) | 0.2 ( 0.4 ) | 0 ( 0 ) | 0 ( 0 ) | 0 ( 0 ) | 0 ( 0 ) | 0 ( 0 ) | 0 ( 0 ) | 0 ( 0 ) | 0 ( 0 ) | 0 ( 0 ) | 0 ( 0 ) | 0 ( 0 ) |
| Ongokea gore | 0 ( 0 ) | 0 ( 0 ) | 0 ( 0 ) | 0 ( 0 ) | 0 ( 0 ) | 0.2 ( 0.4 ) | 0 ( 0 ) | 0 ( 0 ) | 0 ( 0 ) | 0 ( 0 ) | 0 ( 0 ) | 0 ( 0 ) | 0 ( 0 ) |
| Pancovia harmsiana | 8.5 ( 3.5 ) | 0.5 ( 0.8 ) | 0 ( 0 ) | 0 ( 0 ) | 0 ( 0 ) | 0 ( 0 ) | 0 ( 0 ) | 0 ( 0 ) | 0 ( 0 ) | 0 ( 0 ) | 0 ( 0 ) | 0 ( 0 ) | 0 ( 0 ) |
| Pancovia laurentii | 0.8 ( 1.2 ) | 0.8 ( 0.8 ) | 0.5 ( 0.8 ) | 0 ( 0 ) | 0 ( 0 ) | 0 ( 0 ) | 0 ( 0 ) | 0 ( 0 ) | 0 ( 0 ) | 0 ( 0 ) | 0 ( 0 ) | 0 ( 0 ) | 0 ( 0 ) |
| Panda oleosa | 6.5 ( 3.1 ) | 4.2 ( 2.1 ) | 2 ( 0.9 ) | 1 ( 1.3 ) | 0.7 ( 0.5 ) | 0 ( 0 ) | 0 ( 0 ) | 0 ( 0 ) | 0 ( 0 ) | 0 ( 0 ) | 0 ( 0 ) | 0 ( 0 ) | 0 ( 0 ) |
| Paramacrolobium coeruleum | 0.3 ( 0.8 ) | 0.2 ( 0.4 ) | 0.2 ( 0.4 ) | 0 ( 0 ) | 0 ( 0 ) | 0 ( 0 ) | 0 ( 0 ) | 0 ( 0 ) | 0 ( 0 ) | 0 ( 0 ) | 0 ( 0 ) | 0 ( 0 ) | 0 ( 0 ) |
| Parkia bicolor | 0 ( 0 ) | 0.2 ( 0.4 ) | 0 ( 0 ) | 0 ( 0 ) | 0 ( 0 ) | 0 ( 0 ) | 0 ( 0 ) | 0 ( 0 ) | 0 ( 0 ) | 0 ( 0 ) | 0 ( 0 ) | 0 ( 0 ) | 0 ( 0 ) |
| Pentaclethra macrophylla | 0.8 ( 1 ) | 0.2 ( 0.4 ) | 0.2 ( 0.4 ) | 0 ( 0 ) | 0 ( 0 ) | 0 ( 0 ) | 0 ( 0 ) | 0 ( 0 ) | 0 ( 0 ) | 0 ( 0 ) | 0 ( 0 ) | 0 ( 0 ) | 0 ( 0 ) |
| Pericopsis elata | 0.3 ( 0.5 ) | 0 ( 0 ) | 0 ( 0 ) | 0 ( 0 ) | 0 ( 0 ) | 0.2 ( 0.4 ) | 0 ( 0 ) | 0 ( 0 ) | 0 ( 0 ) | 0 ( 0 ) | 0 ( 0 ) | 0 ( 0 ) | 0 ( 0 ) |
| Petersianthus macrocarpus | 7.5 ( 6 ) | 3 ( 2.6 ) | 1.7 ( 2.2 ) | 0.7 ( 0.8 ) | 0.3 ( 0.5 ) | 0.2 ( 0.4 ) | 0 ( 0 ) | 0.2 ( 0.4 ) | 0.2 ( 0.4 ) | 0 ( 0 ) | 0 ( 0 ) | 0 ( 0 ) | 0 ( 0 ) |
| Phyllocosmus africanus | 0 ( 0 ) | 0 ( 0 ) | 0.2 ( 0.4 ) | 0 ( 0 ) | 0 ( 0 ) | 0.2 ( 0.4 ) | 0 ( 0 ) | 0 ( 0 ) | 0 ( 0 ) | 0 ( 0 ) | 0 ( 0 ) | 0.2 ( 0.4 ) | 0 ( 0 ) |
| Piptadeniastrum africanum | 0.2 ( 0.4 ) | 0.2 ( 0.4 ) | 0 ( 0 ) | 0 ( 0 ) | 0 ( 0 ) | 0 ( 0 ) | 0 ( 0 ) | 0 ( 0 ) | 0 ( 0 ) | 0 ( 0 ) | 0 ( 0 ) | 0.2 ( 0.4 ) | 0 ( 0 ) |
| Polyalthia suaveolens | 7 ( 4.7 ) | 9.3 ( 3.8 ) | 6.7 ( 2.3 ) | 2.3 ( 1.8 ) | 1.2 ( 1.3 ) | 0 ( 0 ) | 0 ( 0 ) | 0 ( 0 ) | 0 ( 0 ) | 0 ( 0 ) | 0 ( 0 ) | 0 ( 0 ) | 0 ( 0 ) |
| Prioria balsamifera | 1.7 ( 1.8 ) | 0.3 ( 0.8 ) | 0.7 ( 0.8 ) | 0.2 ( 0.4 ) | 0 ( 0 ) | 0.3 ( 0.5 ) | 0.3 ( 0.5 ) | 0 ( 0 ) | 0.2 ( 0.4 ) | 0 ( 0 ) | 0 ( 0 ) | 0 ( 0 ) | 0 ( 0 ) |
| Prioria joveri | 0.2 ( 0.4 ) | 0.2 ( 0.4 ) | 0 ( 0 ) | 0 ( 0 ) | 0 ( 0 ) | 0 ( 0 ) | 0 ( 0 ) | 0 ( 0 ) | 0 ( 0 ) | 0 ( 0 ) | 0 ( 0 ) | 0 ( 0 ) | 0 ( 0 ) |
| Prioria oxyphylla | 2.7 ( 0.5 ) | 0.7 ( 0.5 ) | 0.5 ( 0.5 ) | 0.7 ( 0.5 ) | 1 ( 0.6 ) | 0.3 ( 0.5 ) | 0 ( 0 ) | 0.5 ( 0.8 ) | 0.3 ( 0.5 ) | 0.2 ( 0.4 ) | 0 ( 0 ) | 0.2 ( 0.4 ) | 0 ( 0 ) |
| Pterocarpus soyauxii | 0.7 ( 0.8 ) | 0.2 ( 0.4 ) | 0.2 ( 0.4 ) | 0 ( 0 ) | 0 ( 0 ) | 0.2 ( 0.4 ) | 0.2 ( 0.4 ) | 0 ( 0 ) | 0.2 ( 0.4 ) | 0 ( 0 ) | 0 ( 0 ) | 0.2 ( 0.4 ) | 0 ( 0 ) |
| Pterygota bequaertii | 0.2 ( 0.4 ) | 0 ( 0 ) | 0 ( 0 ) | 0 ( 0 ) | 0 ( 0 ) | 0 ( 0 ) | 0 ( 0 ) | 0 ( 0 ) | 0 ( 0 ) | 0 ( 0 ) | 0 ( 0 ) | 0 ( 0 ) | 0 ( 0 ) |
| Pycnanthus angolensis | 8 ( 4.9 ) | 1.5 ( 2 ) | 0 ( 0 ) | 0 ( 0 ) | 0 ( 0 ) | 0 ( 0 ) | 0 ( 0 ) | 0 ( 0 ) | 0 ( 0 ) | 0 ( 0 ) | 0 ( 0 ) | 0 ( 0 ) | 0 ( 0 ) |
| Quassia undulata | 0.2 ( 0.4 ) | 0 ( 0 ) | 0 ( 0 ) | 0 ( 0 ) | 0 ( 0 ) | 0 ( 0 ) | 0 ( 0 ) | 0 ( 0 ) | 0 ( 0 ) | 0 ( 0 ) | 0 ( 0 ) | 0 ( 0 ) | 0 ( 0 ) |
| Ricinodendron heudelotii | 0.5 ( 0.8 ) | 0.3 ( 0.5 ) | 0 ( 0 ) | 0 ( 0 ) | 0 ( 0 ) | 0 ( 0 ) | 0 ( 0 ) | 0 ( 0 ) | 0 ( 0 ) | 0 ( 0 ) | 0 ( 0 ) | 0 ( 0 ) | 0 ( 0 ) |
| Rinorea oblongifolia | 0.5 ( 0.8 ) | 0.2 ( 0.4 ) | 0 ( 0 ) | 0 ( 0 ) | 0 ( 0 ) | 0 ( 0 ) | 0 ( 0 ) | 0 ( 0 ) | 0 ( 0 ) | 0 ( 0 ) | 0 ( 0 ) | 0 ( 0 ) | 0 ( 0 ) |
| Rinorea sp. | 3.2 ( 2 ) | 0.2 ( 0.4 ) | 0 ( 0 ) | 0 ( 0 ) | 0 ( 0 ) | 0 ( 0 ) | 0 ( 0 ) | 0 ( 0 ) | 0 ( 0 ) | 0 ( 0 ) | 0 ( 0 ) | 0 ( 0 ) | 0 ( 0 ) |
| Rothmania libisa | 0.3 ( 0.5 ) | 0 ( 0 ) | 0 ( 0 ) | 0 ( 0 ) | 0 ( 0 ) | 0 ( 0 ) | 0 ( 0 ) | 0 ( 0 ) | 0 ( 0 ) | 0 ( 0 ) | 0 ( 0 ) | 0 ( 0 ) | 0 ( 0 ) |
| Rothmannia lujae | 0.5 ( 1.2 ) | 0.2 ( 0.4 ) | 0 ( 0 ) | 0 ( 0 ) | 0 ( 0 ) | 0 ( 0 ) | 0 ( 0 ) | 0 ( 0 ) | 0 ( 0 ) | 0 ( 0 ) | 0 ( 0 ) | 0 ( 0 ) | 0 ( 0 ) |
| Rothmannia sp. | 0.7 ( 0.8 ) | 0 ( 0 ) | 0 ( 0 ) | 0 ( 0 ) | 0 ( 0 ) | 0 ( 0 ) | 0 ( 0 ) | 0 ( 0 ) | 0 ( 0 ) | 0 ( 0 ) | 0 ( 0 ) | 0 ( 0 ) | 0 ( 0 ) |
| Scorodophloeus zenkeri | 7.3 ( 2.9 ) | 6 ( 3.7 ) | 7.2 ( 2.1 ) | 4.7 ( 2.2 ) | 3.8 ( 2.5 ) | 5.7 ( 2.5 ) | 2.3 ( 1.4 ) | 2 ( 1.4 ) | 0.5 ( 0.5 ) | 0.2 ( 0.4 ) | 0 ( 0 ) | 0 ( 0 ) | 0 ( 0 ) |
| Scottellia kamerunensis | 0.2 ( 0.4 ) | 0 ( 0 ) | 0 ( 0 ) | 0 ( 0 ) | 0 ( 0 ) | 0 ( 0 ) | 0 ( 0 ) | 0 ( 0 ) | 0 ( 0 ) | 0 ( 0 ) | 0 ( 0 ) | 0 ( 0 ) | 0 ( 0 ) |
| Staudtia kamerunensis | 13 ( 4.6 ) | 0.7 ( 0.8 ) | 0.2 ( 0.4 ) | 0 ( 0 ) | 0.2 ( 0.4 ) | 0 ( 0 ) | 0 ( 0 ) | 0 ( 0 ) | 0 ( 0 ) | 0 ( 0 ) | 0 ( 0 ) | 0 ( 0 ) | 0 ( 0 ) |
| Sterculia bequaertii | 0 ( 0 ) | 0.2 ( 0.4 ) | 0 ( 0 ) | 0 ( 0 ) | 0 ( 0 ) | 0 ( 0 ) | 0 ( 0 ) | 0 ( 0 ) | 0 ( 0 ) | 0 ( 0 ) | 0 ( 0 ) | 0 ( 0 ) | 0 ( 0 ) |
| Sterculia tragacantha | 0.7 ( 1.2 ) | 0 ( 0 ) | 0 ( 0 ) | 0.3 ( 0.5 ) | 0.5 ( 0.8 ) | 0 ( 0 ) | 0 ( 0 ) | 0 ( 0 ) | 0 ( 0 ) | 0 ( 0 ) | 0 ( 0 ) | 0 ( 0 ) | 0 ( 0 ) |
| Strombosia grandifolia | 1.2 ( 1 ) | 0.3 ( 0.5 ) | 0 ( 0 ) | 0 ( 0 ) | 0 ( 0 ) | 0 ( 0 ) | 0 ( 0 ) | 0 ( 0 ) | 0 ( 0 ) | 0 ( 0 ) | 0 ( 0 ) | 0 ( 0 ) | 0 ( 0 ) |
| Strombosia nigropunctata | 5.2 ( 3.2 ) | 1.7 ( 0.8 ) | 0.2 ( 0.4 ) | 0.3 ( 0.5 ) | 0 ( 0 ) | 0 ( 0 ) | 0 ( 0 ) | 0 ( 0 ) | 0 ( 0 ) | 0 ( 0 ) | 0 ( 0 ) | 0 ( 0 ) | 0 ( 0 ) |
| Strombosia pustulata | 1.8 ( 1.7 ) | 0.7 ( 0.8 ) | 0.7 ( 0.5 ) | 0 ( 0 ) | 0 ( 0 ) | 0 ( 0 ) | 0 ( 0 ) | 0 ( 0 ) | 0 ( 0 ) | 0 ( 0 ) | 0 ( 0 ) | 0 ( 0 ) | 0 ( 0 ) |
| Strombosiopsis tetrandra | 0.3 ( 0.5 ) | 0.2 ( 0.4 ) | 0.5 ( 0.5 ) | 0 ( 0 ) | 0 ( 0 ) | 0 ( 0 ) | 0 ( 0 ) | 0 ( 0 ) | 0 ( 0 ) | 0 ( 0 ) | 0 ( 0 ) | 0 ( 0 ) | 0 ( 0 ) |
| Symphonia globulifera | 0.2 ( 0.4 ) | 0.2 ( 0.4 ) | 0 ( 0 ) | 0 ( 0 ) | 0.2 ( 0.4 ) | 0 ( 0 ) | 0 ( 0 ) | 0 ( 0 ) | 0 ( 0 ) | 0 ( 0 ) | 0 ( 0 ) | 0 ( 0 ) | 0 ( 0 ) |
| Synsepalum subcordatum | 0.5 ( 0.5 ) | 0 ( 0 ) | 0 ( 0 ) | 0.2 ( 0.4 ) | 0 ( 0 ) | 0 ( 0 ) | 0 ( 0 ) | 0 ( 0 ) | 0 ( 0 ) | 0 ( 0 ) | 0 ( 0 ) | 0 ( 0 ) | 0.2 ( 0.4 ) |
| Syzygium congolense | 0.2 ( 0.4 ) | 0 ( 0 ) | 0 ( 0 ) | 0 ( 0 ) | 0 ( 0 ) | 0 ( 0 ) | 0 ( 0 ) | 0 ( 0 ) | 0 ( 0 ) | 0 ( 0 ) | 0 ( 0 ) | 0 ( 0 ) | 0 ( 0 ) |
| Tessmannia africana | 1.2 ( 1.5 ) | 0 ( 0 ) | 0 ( 0 ) | 0 ( 0 ) | 0.2 ( 0.4 ) | 0.2 ( 0.4 ) | 0 ( 0 ) | 0 ( 0 ) | 0 ( 0 ) | 0 ( 0 ) | 0 ( 0 ) | 0 ( 0 ) | 0 ( 0 ) |
| Tessmannia anomala | 0.3 ( 0.5 ) | 0 ( 0 ) | 0 ( 0 ) | 0 ( 0 ) | 0 ( 0 ) | 0 ( 0 ) | 0 ( 0 ) | 0.2 ( 0.4 ) | 0 ( 0 ) | 0 ( 0 ) | 0 ( 0 ) | 0 ( 0 ) | 0 ( 0 ) |
| Tetrapleura tetraptera | 0 ( 0 ) | 0.2 ( 0.4 ) | 0 ( 0 ) | 0 ( 0 ) | 0 ( 0 ) | 0 ( 0 ) | 0 ( 0 ) | 0 ( 0 ) | 0 ( 0 ) | 0 ( 0 ) | 0 ( 0 ) | 0 ( 0 ) | 0 ( 0 ) |
| Tetrorchidium didymostemon | 0.3 ( 0.5 ) | 0.2 ( 0.4 ) | 0 ( 0 ) | 0 ( 0 ) | 0 ( 0 ) | 0 ( 0 ) | 0 ( 0 ) | 0 ( 0 ) | 0 ( 0 ) | 0 ( 0 ) | 0 ( 0 ) | 0 ( 0 ) | 0 ( 0 ) |
| Treculia africana | 0 ( 0 ) | 0 ( 0 ) | 0 ( 0 ) | 0 ( 0 ) | 0.2 ( 0.4 ) | 0 ( 0 ) | 0 ( 0 ) | 0 ( 0 ) | 0 ( 0 ) | 0 ( 0 ) | 0 ( 0 ) | 0 ( 0 ) | 0 ( 0 ) |
| Trichilia gilgiana | 0.5 ( 1.2 ) | 0.2 ( 0.4 ) | 0.2 ( 0.4 ) | 0 ( 0 ) | 0 ( 0 ) | 0 ( 0 ) | 0 ( 0 ) | 0 ( 0 ) | 0 ( 0 ) | 0 ( 0 ) | 0 ( 0 ) | 0 ( 0 ) | 0 ( 0 ) |
| Trichilia prieuriana | 5.7 ( 4.1 ) | 2.7 ( 2.3 ) | 0.5 ( 0.8 ) | 0.3 ( 0.8 ) | 0.2 ( 0.4 ) | 0 ( 0 ) | 0 ( 0 ) | 0 ( 0 ) | 0 ( 0 ) | 0 ( 0 ) | 0 ( 0 ) | 0 ( 0 ) | 0 ( 0 ) |
| Trichilia sp. | 1.7 ( 2 ) | 0.8 ( 1.6 ) | 0.8 ( 1 ) | 0 ( 0 ) | 0 ( 0 ) | 0 ( 0 ) | 0 ( 0 ) | 0 ( 0 ) | 0 ( 0 ) | 0 ( 0 ) | 0 ( 0 ) | 0 ( 0 ) | 0 ( 0 ) |
| Trichilia welwitschii | 2.3 ( 2.7 ) | 0 ( 0 ) | 0 ( 0 ) | 0 ( 0 ) | 0 ( 0 ) | 0 ( 0 ) | 0 ( 0 ) | 0 ( 0 ) | 0 ( 0 ) | 0 ( 0 ) | 0 ( 0 ) | 0 ( 0 ) | 0 ( 0 ) |
| Trichoscypha oddonii | 0.2 ( 0.4 ) | 0 ( 0 ) | 0 ( 0 ) | 0 ( 0 ) | 0 ( 0 ) | 0 ( 0 ) | 0 ( 0 ) | 0 ( 0 ) | 0 ( 0 ) | 0 ( 0 ) | 0 ( 0 ) | 0 ( 0 ) | 0 ( 0 ) |
| Tridesmostemon omphalocarpoides | 0.7 ( 0.8 ) | 0.7 ( 1 ) | 0.2 ( 0.4 ) | 0 ( 0 ) | 0.2 ( 0.4 ) | 0 ( 0 ) | 0 ( 0 ) | 0 ( 0 ) | 0 ( 0 ) | 0 ( 0 ) | 0 ( 0 ) | 0 ( 0 ) | 0 ( 0 ) |
| Turraeanthus africanus | 5.7 ( 8.7 ) | 0.7 ( 1.2 ) | 0.2 ( 0.4 ) | 0 ( 0 ) | 0.2 ( 0.4 ) | 0.2 ( 0.4 ) | 0 ( 0 ) | 0 ( 0 ) | 0 ( 0 ) | 0 ( 0 ) | 0 ( 0 ) | 0 ( 0 ) | 0 ( 0 ) |
| Unknown | 17.8 ( 7.7 ) | 4.5 ( 3.1 ) | 1.3 ( 2.3 ) | 2.2 ( 1.9 ) | 2.2 ( 1.7 ) | 1.7 ( 1.2 ) | 1.3 ( 1.2 ) | 0.7 ( 0.8 ) | 0 ( 0 ) | 0 ( 0 ) | 0 ( 0 ) | 0 ( 0 ) | 0 ( 0 ) |
| Vitex welwitschii | 0.5 ( 0.8 ) | 0.2 ( 0.4 ) | 0 ( 0 ) | 0.2 ( 0.4 ) | 0 ( 0 ) | 0 ( 0 ) | 0 ( 0 ) | 0 ( 0 ) | 0 ( 0 ) | 0 ( 0 ) | 0 ( 0 ) | 0 ( 0 ) | 0 ( 0 ) |
| Xylia ghesquierei | 0 ( 0 ) | 0 ( 0 ) | 0 ( 0 ) | 0.2 ( 0.4 ) | 0.2 ( 0.4 ) | 0 ( 0 ) | 0 ( 0 ) | 0 ( 0 ) | 0 ( 0 ) | 0 ( 0 ) | 0 ( 0 ) | 0 ( 0 ) | 0 ( 0 ) |
| Xylopia hypolampra | 0.2 ( 0.4 ) | 0.2 ( 0.4 ) | 0 ( 0 ) | 0.2 ( 0.4 ) | 0 ( 0 ) | 0 ( 0 ) | 0 ( 0 ) | 0 ( 0 ) | 0 ( 0 ) | 0 ( 0 ) | 0 ( 0 ) | 0 ( 0 ) | 0 ( 0 ) |
| Zanthoxylum inaequalis | 0 ( 0 ) | 0 ( 0 ) | 0 ( 0 ) | 0.2 ( 0.4 ) | 0 ( 0 ) | 0 ( 0 ) | 0 ( 0 ) | 0 ( 0 ) | 0 ( 0 ) | 0 ( 0 ) | 0 ( 0 ) | 0 ( 0 ) | 0 ( 0 ) |

**S3 Table.** Average number of stems with standard deviation in brackets of tree species per hectare in Yangambi within different diameter classes.

| **Species** | **10-20** | **20-30** | **30-40** | **40-50** | **50-60** | **60-70** | **70-80** | **80-90** | **90-100** | **100-110** | **110-120** | **120-130** | **130-140** | **140-150** | **150-160** |
| --- | --- | --- | --- | --- | --- | --- | --- | --- | --- | --- | --- | --- | --- | --- | --- |
| Afrostyrax lepidophyllus | 0.6 ( 1.3 ) | 0 ( 0 ) | 0 ( 0 ) | 0.2 ( 0.4 ) | 0 ( 0 ) | 0 ( 0 ) | 0 ( 0 ) | 0 ( 0 ) | 0 ( 0 ) | 0 ( 0 ) | 0 ( 0 ) | 0 ( 0 ) | 0 ( 0 ) | 0 ( 0 ) | 0 ( 0 ) |
| Afzelia bipindensis | 0 ( 0 ) | 0.2 ( 0.4 ) | 0 ( 0 ) | 0 ( 0 ) | 0 ( 0 ) | 0 ( 0 ) | 0 ( 0 ) | 0 ( 0 ) | 0 ( 0 ) | 0 ( 0 ) | 0 ( 0 ) | 0 ( 0 ) | 0 ( 0 ) | 0 ( 0 ) | 0 ( 0 ) |
| Aidia micrantha | 0.4 ( 0.9 ) | 0 ( 0 ) | 0 ( 0 ) | 0 ( 0 ) | 0 ( 0 ) | 0 ( 0 ) | 0 ( 0 ) | 0 ( 0 ) | 0 ( 0 ) | 0 ( 0 ) | 0 ( 0 ) | 0 ( 0 ) | 0 ( 0 ) | 0 ( 0 ) | 0 ( 0 ) |
| Albizia sp. | 0.4 ( 0.9 ) | 0 ( 0 ) | 0 ( 0 ) | 0.2 ( 0.4 ) | 0.2 ( 0.4 ) | 0 ( 0 ) | 0 ( 0 ) | 0 ( 0 ) | 0 ( 0 ) | 0 ( 0 ) | 0 ( 0 ) | 0 ( 0 ) | 0 ( 0 ) | 0 ( 0 ) | 0 ( 0 ) |
| Allanblackia floribunda | 0.4 ( 0.9 ) | 0 ( 0 ) | 0 ( 0 ) | 0 ( 0 ) | 0 ( 0 ) | 0 ( 0 ) | 0 ( 0 ) | 0 ( 0 ) | 0 ( 0 ) | 0 ( 0 ) | 0 ( 0 ) | 0 ( 0 ) | 0 ( 0 ) | 0 ( 0 ) | 0 ( 0 ) |
| Alstonia boonei | 0 ( 0 ) | 0 ( 0 ) | 0.2 ( 0.4 ) | 0 ( 0 ) | 0.2 ( 0.4 ) | 0 ( 0 ) | 0 ( 0 ) | 0 ( 0 ) | 0 ( 0 ) | 0.2 ( 0.4 ) | 0 ( 0 ) | 0 ( 0 ) | 0 ( 0 ) | 0 ( 0 ) | 0 ( 0 ) |
| Anonidium mannii | 2.6 ( 2.7 ) | 4 ( 3.9 ) | 4.8 ( 5.2 ) | 3.2 ( 2.6 ) | 0.6 ( 1.3 ) | 0 ( 0 ) | 0 ( 0 ) | 0 ( 0 ) | 0 ( 0 ) | 0.2 ( 0.4 ) | 0 ( 0 ) | 0 ( 0 ) | 0 ( 0 ) | 0 ( 0 ) | 0 ( 0 ) |
| Anthonotha macrophylla | 1 ( 1.2 ) | 0 ( 0 ) | 0 ( 0 ) | 0 ( 0 ) | 0 ( 0 ) | 0 ( 0 ) | 0 ( 0 ) | 0 ( 0 ) | 0 ( 0 ) | 0 ( 0 ) | 0 ( 0 ) | 0 ( 0 ) | 0 ( 0 ) | 0 ( 0 ) | 0 ( 0 ) |
| Antrocaryon nannanii | 0 ( 0 ) | 0 ( 0 ) | 0 ( 0 ) | 0.2 ( 0.4 ) | 0 ( 0 ) | 0 ( 0 ) | 0 ( 0 ) | 0 ( 0 ) | 0 ( 0 ) | 0 ( 0 ) | 0 ( 0 ) | 0 ( 0 ) | 0 ( 0 ) | 0 ( 0 ) | 0 ( 0 ) |
| Autranella congolensis | 0 ( 0 ) | 0 ( 0 ) | 0 ( 0 ) | 0 ( 0 ) | 0 ( 0 ) | 0 ( 0 ) | 0 ( 0 ) | 0 ( 0 ) | 0 ( 0 ) | 0 ( 0 ) | 0 ( 0 ) | 0 ( 0 ) | 0 ( 0 ) | 0 ( 0 ) | 0.2 ( 0.4 ) |
| Baphia capparidifolia | 0.2 ( 0.4 ) | 0 ( 0 ) | 0 ( 0 ) | 0 ( 0 ) | 0 ( 0 ) | 0 ( 0 ) | 0 ( 0 ) | 0 ( 0 ) | 0 ( 0 ) | 0 ( 0 ) | 0 ( 0 ) | 0 ( 0 ) | 0 ( 0 ) | 0 ( 0 ) | 0 ( 0 ) |
| Barteria fistulosa | 0.4 ( 0.9 ) | 0 ( 0 ) | 0 ( 0 ) | 0 ( 0 ) | 0 ( 0 ) | 0 ( 0 ) | 0 ( 0 ) | 0 ( 0 ) | 0 ( 0 ) | 0 ( 0 ) | 0 ( 0 ) | 0 ( 0 ) | 0 ( 0 ) | 0 ( 0 ) | 0 ( 0 ) |
| Barteria nigritiana | 0.4 ( 0.5 ) | 0 ( 0 ) | 0 ( 0 ) | 0 ( 0 ) | 0 ( 0 ) | 0 ( 0 ) | 0 ( 0 ) | 0 ( 0 ) | 0 ( 0 ) | 0 ( 0 ) | 0 ( 0 ) | 0 ( 0 ) | 0 ( 0 ) | 0 ( 0 ) | 0 ( 0 ) |
| Beilschmiedia gilbertii | 0.2 ( 0.4 ) | 0 ( 0 ) | 0 ( 0 ) | 0 ( 0 ) | 0 ( 0 ) | 0 ( 0 ) | 0 ( 0 ) | 0 ( 0 ) | 0 ( 0 ) | 0 ( 0 ) | 0 ( 0 ) | 0 ( 0 ) | 0 ( 0 ) | 0 ( 0 ) | 0 ( 0 ) |
| Blighia welwitschii | 0.6 ( 0.5 ) | 0.4 ( 0.5 ) | 0.2 ( 0.4 ) | 0 ( 0 ) | 0.2 ( 0.4 ) | 0 ( 0 ) | 0 ( 0 ) | 0 ( 0 ) | 0 ( 0 ) | 0 ( 0 ) | 0 ( 0 ) | 0 ( 0 ) | 0 ( 0 ) | 0 ( 0 ) | 0 ( 0 ) |
| Canarium schweinfurthii | 0.6 ( 1.3 ) | 0 ( 0 ) | 0 ( 0 ) | 0 ( 0 ) | 0 ( 0 ) | 0 ( 0 ) | 0 ( 0 ) | 0 ( 0 ) | 0 ( 0 ) | 0 ( 0 ) | 0 ( 0 ) | 0 ( 0 ) | 0 ( 0 ) | 0 ( 0 ) | 0 ( 0 ) |
| Carapa procera | 12 ( 1.7 ) | 0.4 ( 0.5 ) | 0 ( 0 ) | 0 ( 0 ) | 0 ( 0 ) | 0 ( 0 ) | 0 ( 0 ) | 0 ( 0 ) | 0 ( 0 ) | 0 ( 0 ) | 0 ( 0 ) | 0 ( 0 ) | 0 ( 0 ) | 0 ( 0 ) | 0 ( 0 ) |
| Celtis mildbraedii | 0.6 ( 0.9 ) | 1.4 ( 1.3 ) | 0.2 ( 0.4 ) | 0 ( 0 ) | 0 ( 0 ) | 0.2 ( 0.4 ) | 0.2 ( 0.4 ) | 0.4 ( 0.5 ) | 0 ( 0 ) | 0 ( 0 ) | 0 ( 0 ) | 0 ( 0 ) | 0 ( 0 ) | 0 ( 0 ) | 0 ( 0 ) |
| Celtis sp. | 0.2 ( 0.4 ) | 0 ( 0 ) | 0 ( 0 ) | 0 ( 0 ) | 0 ( 0 ) | 0 ( 0 ) | 0 ( 0 ) | 0 ( 0 ) | 0 ( 0 ) | 0 ( 0 ) | 0 ( 0 ) | 0 ( 0 ) | 0 ( 0 ) | 0 ( 0 ) | 0 ( 0 ) |
| Celtis tessmannii | 1.4 ( 0.5 ) | 1.4 ( 0.9 ) | 0.2 ( 0.4 ) | 0.6 ( 0.5 ) | 0 ( 0 ) | 0 ( 0 ) | 0 ( 0 ) | 0 ( 0 ) | 0 ( 0 ) | 0 ( 0 ) | 0 ( 0 ) | 0 ( 0 ) | 0 ( 0 ) | 0 ( 0 ) | 0 ( 0 ) |
| Chrysophyllum africanum | 1.8 ( 0.8 ) | 0.8 ( 1.3 ) | 0.4 ( 0.5 ) | 0.2 ( 0.4 ) | 0.2 ( 0.4 ) | 0 ( 0 ) | 0 ( 0 ) | 0 ( 0 ) | 0 ( 0 ) | 0 ( 0 ) | 0 ( 0 ) | 0 ( 0 ) | 0 ( 0 ) | 0 ( 0 ) | 0 ( 0 ) |
| Chrysophyllum beguei | 0.4 ( 0.9 ) | 0.4 ( 0.5 ) | 0 ( 0 ) | 0 ( 0 ) | 0 ( 0 ) | 0 ( 0 ) | 0 ( 0 ) | 0 ( 0 ) | 0 ( 0 ) | 0 ( 0 ) | 0 ( 0 ) | 0 ( 0 ) | 0 ( 0 ) | 0 ( 0 ) | 0 ( 0 ) |
| Chrysophyllum lacourtianum | 1 ( 1.4 ) | 0.6 ( 0.9 ) | 0.6 ( 0.9 ) | 0.8 ( 0.4 ) | 1 ( 1.2 ) | 0.6 ( 0.9 ) | 0.4 ( 0.9 ) | 0 ( 0 ) | 0 ( 0 ) | 0 ( 0 ) | 0 ( 0 ) | 0 ( 0 ) | 0 ( 0 ) | 0 ( 0 ) | 0 ( 0 ) |
| Chrysophyllum pruniforme | 0 ( 0 ) | 0 ( 0 ) | 0.2 ( 0.4 ) | 0 ( 0 ) | 0.2 ( 0.4 ) | 0 ( 0 ) | 0 ( 0 ) | 0 ( 0 ) | 0 ( 0 ) | 0 ( 0 ) | 0 ( 0 ) | 0 ( 0 ) | 0 ( 0 ) | 0 ( 0 ) | 0 ( 0 ) |
| Chrysophyllum pruniformis | 0.2 ( 0.4 ) | 0 ( 0 ) | 0 ( 0 ) | 0 ( 0 ) | 0 ( 0 ) | 0 ( 0 ) | 0 ( 0 ) | 0 ( 0 ) | 0 ( 0 ) | 0 ( 0 ) | 0 ( 0 ) | 0 ( 0 ) | 0 ( 0 ) | 0 ( 0 ) | 0 ( 0 ) |
| Chrysophyllum sp. | 0.2 ( 0.4 ) | 0 ( 0 ) | 0.2 ( 0.4 ) | 0 ( 0 ) | 0 ( 0 ) | 0.4 ( 0.9 ) | 0 ( 0 ) | 0 ( 0 ) | 0 ( 0 ) | 0 ( 0 ) | 0 ( 0 ) | 0 ( 0 ) | 0 ( 0 ) | 0 ( 0 ) | 0 ( 0 ) |
| Cleistanthus pynaertii | 0.2 ( 0.4 ) | 0 ( 0 ) | 0 ( 0 ) | 0 ( 0 ) | 0 ( 0 ) | 0 ( 0 ) | 0 ( 0 ) | 0 ( 0 ) | 0 ( 0 ) | 0 ( 0 ) | 0 ( 0 ) | 0 ( 0 ) | 0 ( 0 ) | 0 ( 0 ) | 0 ( 0 ) |
| Coelocaryon preussii | 7.4 ( 7.2 ) | 0.8 ( 1.8 ) | 0.2 ( 0.4 ) | 0 ( 0 ) | 0 ( 0 ) | 0 ( 0 ) | 0 ( 0 ) | 0 ( 0 ) | 0 ( 0 ) | 0 ( 0 ) | 0 ( 0 ) | 0 ( 0 ) | 0 ( 0 ) | 0 ( 0 ) | 0 ( 0 ) |
| Cola acuminata | 0 ( 0 ) | 0.2 ( 0.4 ) | 0 ( 0 ) | 0 ( 0 ) | 0 ( 0 ) | 0 ( 0 ) | 0 ( 0 ) | 0 ( 0 ) | 0 ( 0 ) | 0 ( 0 ) | 0 ( 0 ) | 0 ( 0 ) | 0 ( 0 ) | 0 ( 0 ) | 0 ( 0 ) |
| Cola griseiflora | 7.8 ( 6.9 ) | 1.4 ( 1.9 ) | 0.2 ( 0.4 ) | 0 ( 0 ) | 0 ( 0 ) | 0 ( 0 ) | 0 ( 0 ) | 0 ( 0 ) | 0 ( 0 ) | 0 ( 0 ) | 0 ( 0 ) | 0 ( 0 ) | 0 ( 0 ) | 0 ( 0 ) | 0 ( 0 ) |
| Cola lateritia | 0.4 ( 0.5 ) | 0.4 ( 0.5 ) | 0.4 ( 0.9 ) | 0 ( 0 ) | 0.2 ( 0.4 ) | 0.2 ( 0.4 ) | 0 ( 0 ) | 0 ( 0 ) | 0 ( 0 ) | 0 ( 0 ) | 0 ( 0 ) | 0 ( 0 ) | 0 ( 0 ) | 0 ( 0 ) | 0 ( 0 ) |
| Cola sp. | 0 ( 0 ) | 0.2 ( 0.4 ) | 0 ( 0 ) | 0 ( 0 ) | 0 ( 0 ) | 0 ( 0 ) | 0 ( 0 ) | 0 ( 0 ) | 0 ( 0 ) | 0 ( 0 ) | 0 ( 0 ) | 0 ( 0 ) | 0 ( 0 ) | 0 ( 0 ) | 0 ( 0 ) |
| Combretum lokele | 0.6 ( 0.9 ) | 0 ( 0 ) | 0.6 ( 0.9 ) | 0.2 ( 0.4 ) | 0.2 ( 0.4 ) | 0 ( 0 ) | 0 ( 0 ) | 0.2 ( 0.4 ) | 0 ( 0 ) | 0 ( 0 ) | 0 ( 0 ) | 0.2 ( 0.4 ) | 0 ( 0 ) | 0 ( 0 ) | 0 ( 0 ) |
| Cynometra hankei | 0.2 ( 0.4 ) | 0 ( 0 ) | 0 ( 0 ) | 0 ( 0 ) | 0.4 ( 0.5 ) | 0 ( 0 ) | 0 ( 0 ) | 0.2 ( 0.4 ) | 0.2 ( 0.4 ) | 0 ( 0 ) | 0 ( 0 ) | 0.2 ( 0.4 ) | 0 ( 0 ) | 0 ( 0 ) | 0 ( 0 ) |
| Dacryodes edulis | 0.4 ( 0.5 ) | 0.2 ( 0.4 ) | 0 ( 0 ) | 0 ( 0 ) | 0 ( 0 ) | 0 ( 0 ) | 0 ( 0 ) | 0 ( 0 ) | 0 ( 0 ) | 0 ( 0 ) | 0 ( 0 ) | 0 ( 0 ) | 0 ( 0 ) | 0 ( 0 ) | 0 ( 0 ) |
| Dacryodes osika | 0.2 ( 0.4 ) | 0 ( 0 ) | 0 ( 0 ) | 0 ( 0 ) | 0 ( 0 ) | 0 ( 0 ) | 0 ( 0 ) | 0 ( 0 ) | 0 ( 0 ) | 0 ( 0 ) | 0 ( 0 ) | 0 ( 0 ) | 0 ( 0 ) | 0 ( 0 ) | 0 ( 0 ) |
| Desplatsia dewevrei | 0.2 ( 0.4 ) | 0 ( 0 ) | 0.2 ( 0.4 ) | 0 ( 0 ) | 0 ( 0 ) | 0 ( 0 ) | 0 ( 0 ) | 0 ( 0 ) | 0 ( 0 ) | 0 ( 0 ) | 0 ( 0 ) | 0 ( 0 ) | 0 ( 0 ) | 0 ( 0 ) | 0 ( 0 ) |
| Dialium corbisieri | 0.4 ( 0.5 ) | 0 ( 0 ) | 0 ( 0 ) | 0 ( 0 ) | 0 ( 0 ) | 0 ( 0 ) | 0 ( 0 ) | 0 ( 0 ) | 0 ( 0 ) | 0 ( 0 ) | 0 ( 0 ) | 0 ( 0 ) | 0 ( 0 ) | 0 ( 0 ) | 0 ( 0 ) |
| Dialium excelsum | 0 ( 0 ) | 0.2 ( 0.4 ) | 0 ( 0 ) | 0.4 ( 0.9 ) | 0 ( 0 ) | 0 ( 0 ) | 0 ( 0 ) | 0 ( 0 ) | 0 ( 0 ) | 0 ( 0 ) | 0 ( 0 ) | 0 ( 0 ) | 0 ( 0 ) | 0 ( 0 ) | 0 ( 0 ) |
| Dialium pachyphyllum | 3 ( 1.2 ) | 0.4 ( 0.5 ) | 0 ( 0 ) | 0.2 ( 0.4 ) | 0 ( 0 ) | 0.4 ( 0.5 ) | 0 ( 0 ) | 0 ( 0 ) | 0 ( 0 ) | 0 ( 0 ) | 0 ( 0 ) | 0 ( 0 ) | 0 ( 0 ) | 0 ( 0 ) | 0 ( 0 ) |
| Dialium sp. | 0.8 ( 1.3 ) | 0.4 ( 0.9 ) | 0 ( 0 ) | 0.2 ( 0.4 ) | 0 ( 0 ) | 0.2 ( 0.4 ) | 0 ( 0 ) | 0 ( 0 ) | 0 ( 0 ) | 0 ( 0 ) | 0 ( 0 ) | 0 ( 0 ) | 0 ( 0 ) | 0 ( 0 ) | 0 ( 0 ) |
| Diospyros boala | 1.8 ( 1.5 ) | 0.4 ( 0.9 ) | 0 ( 0 ) | 0 ( 0 ) | 0 ( 0 ) | 0 ( 0 ) | 0 ( 0 ) | 0 ( 0 ) | 0 ( 0 ) | 0 ( 0 ) | 0 ( 0 ) | 0 ( 0 ) | 0 ( 0 ) | 0 ( 0 ) | 0 ( 0 ) |
| Diospyros crassiflora | 1 ( 0.7 ) | 0.8 ( 0.8 ) | 0.6 ( 0.5 ) | 0 ( 0 ) | 0.2 ( 0.4 ) | 0 ( 0 ) | 0 ( 0 ) | 0 ( 0 ) | 0 ( 0 ) | 0 ( 0 ) | 0 ( 0 ) | 0 ( 0 ) | 0 ( 0 ) | 0 ( 0 ) | 0 ( 0 ) |
| Diospyros sp. | 3 ( 2.4 ) | 0 ( 0 ) | 0.2 ( 0.4 ) | 0 ( 0 ) | 0 ( 0 ) | 0 ( 0 ) | 0 ( 0 ) | 0 ( 0 ) | 0 ( 0 ) | 0 ( 0 ) | 0 ( 0 ) | 0 ( 0 ) | 0 ( 0 ) | 0 ( 0 ) | 0 ( 0 ) |
| Drypetes angustifolia | 0.2 ( 0.4 ) | 0 ( 0 ) | 0 ( 0 ) | 0 ( 0 ) | 0 ( 0 ) | 0 ( 0 ) | 0 ( 0 ) | 0 ( 0 ) | 0 ( 0 ) | 0 ( 0 ) | 0 ( 0 ) | 0 ( 0 ) | 0 ( 0 ) | 0 ( 0 ) | 0 ( 0 ) |
| Drypetes anthiatif | 0.2 ( 0.4 ) | 0 ( 0 ) | 0 ( 0 ) | 0 ( 0 ) | 0 ( 0 ) | 0 ( 0 ) | 0 ( 0 ) | 0 ( 0 ) | 0 ( 0 ) | 0 ( 0 ) | 0 ( 0 ) | 0 ( 0 ) | 0 ( 0 ) | 0 ( 0 ) | 0 ( 0 ) |
| Drypetes gossweileri | 3.6 ( 3 ) | 2 ( 2 ) | 1 ( 1.2 ) | 0.6 ( 0.9 ) | 0.4 ( 0.5 ) | 0 ( 0 ) | 0 ( 0 ) | 0 ( 0 ) | 0 ( 0 ) | 0 ( 0 ) | 0 ( 0 ) | 0 ( 0 ) | 0 ( 0 ) | 0 ( 0 ) | 0 ( 0 ) |
| Drypetes leonensis | 1 ( 1.7 ) | 0 ( 0 ) | 0 ( 0 ) | 0 ( 0 ) | 0 ( 0 ) | 0 ( 0 ) | 0 ( 0 ) | 0 ( 0 ) | 0 ( 0 ) | 0 ( 0 ) | 0 ( 0 ) | 0 ( 0 ) | 0 ( 0 ) | 0 ( 0 ) | 0 ( 0 ) |
| Drypetes likwa | 0.2 ( 0.4 ) | 0 ( 0 ) | 0 ( 0 ) | 0 ( 0 ) | 0 ( 0 ) | 0 ( 0 ) | 0 ( 0 ) | 0 ( 0 ) | 0 ( 0 ) | 0 ( 0 ) | 0 ( 0 ) | 0 ( 0 ) | 0 ( 0 ) | 0 ( 0 ) | 0 ( 0 ) |
| Drypetes sp. | 4.2 ( 2.9 ) | 1.8 ( 1.6 ) | 0.2 ( 0.4 ) | 0.4 ( 0.5 ) | 0 ( 0 ) | 0 ( 0 ) | 0 ( 0 ) | 0 ( 0 ) | 0 ( 0 ) | 0 ( 0 ) | 0 ( 0 ) | 0 ( 0 ) | 0 ( 0 ) | 0 ( 0 ) | 0 ( 0 ) |
| Entandrophragma angolense | 0.2 ( 0.4 ) | 0 ( 0 ) | 0 ( 0 ) | 0 ( 0 ) | 0 ( 0 ) | 0 ( 0 ) | 0 ( 0 ) | 0 ( 0 ) | 0 ( 0 ) | 0 ( 0 ) | 0 ( 0 ) | 0 ( 0 ) | 0 ( 0 ) | 0 ( 0 ) | 0 ( 0 ) |
| Entandrophragma cylindricum | 0.4 ( 0.5 ) | 0 ( 0 ) | 0.2 ( 0.4 ) | 0 ( 0 ) | 0 ( 0 ) | 0 ( 0 ) | 0.2 ( 0.4 ) | 0 ( 0 ) | 0 ( 0 ) | 0 ( 0 ) | 0 ( 0 ) | 0 ( 0 ) | 0 ( 0 ) | 0 ( 0 ) | 0 ( 0 ) |
| Entandrophragma sp. | 0 ( 0 ) | 0 ( 0 ) | 0 ( 0 ) | 0.2 ( 0.4 ) | 0 ( 0 ) | 0 ( 0 ) | 0 ( 0 ) | 0 ( 0 ) | 0 ( 0 ) | 0 ( 0 ) | 0 ( 0 ) | 0 ( 0 ) | 0 ( 0 ) | 0 ( 0 ) | 0 ( 0 ) |
| Entandrophragma utile | 0.2 ( 0.4 ) | 0.2 ( 0.4 ) | 0 ( 0 ) | 0 ( 0 ) | 0 ( 0 ) | 0 ( 0 ) | 0 ( 0 ) | 0.2 ( 0.4 ) | 0 ( 0 ) | 0.2 ( 0.4 ) | 0 ( 0 ) | 0 ( 0 ) | 0 ( 0 ) | 0 ( 0 ) | 0 ( 0 ) |
| Erythrophleum suaveolens | 0 ( 0 ) | 0.4 ( 0.5 ) | 0.2 ( 0.4 ) | 0 ( 0 ) | 0 ( 0 ) | 0.4 ( 0.5 ) | 0 ( 0 ) | 0.4 ( 0.5 ) | 0 ( 0 ) | 0 ( 0 ) | 0.2 ( 0.4 ) | 0 ( 0 ) | 0 ( 0 ) | 0 ( 0 ) | 0 ( 0 ) |
| Funtumia africana | 0.2 ( 0.4 ) | 0 ( 0 ) | 0 ( 0 ) | 0 ( 0 ) | 0 ( 0 ) | 0 ( 0 ) | 0 ( 0 ) | 0 ( 0 ) | 0 ( 0 ) | 0 ( 0 ) | 0 ( 0 ) | 0 ( 0 ) | 0 ( 0 ) | 0 ( 0 ) | 0 ( 0 ) |
| Garcinia punctata | 5 ( 1.6 ) | 2 ( 2.3 ) | 0.6 ( 0.9 ) | 0 ( 0 ) | 0 ( 0 ) | 0 ( 0 ) | 0 ( 0 ) | 0 ( 0 ) | 0 ( 0 ) | 0 ( 0 ) | 0 ( 0 ) | 0 ( 0 ) | 0 ( 0 ) | 0 ( 0 ) | 0 ( 0 ) |
| Garcinia smeathmannii | 1.2 ( 1.8 ) | 0 ( 0 ) | 0 ( 0 ) | 0 ( 0 ) | 0 ( 0 ) | 0 ( 0 ) | 0 ( 0 ) | 0 ( 0 ) | 0 ( 0 ) | 0 ( 0 ) | 0 ( 0 ) | 0 ( 0 ) | 0 ( 0 ) | 0 ( 0 ) | 0 ( 0 ) |
| Garcinia sp. | 0 ( 0 ) | 0.2 ( 0.4 ) | 0 ( 0 ) | 0 ( 0 ) | 0 ( 0 ) | 0 ( 0 ) | 0 ( 0 ) | 0 ( 0 ) | 0 ( 0 ) | 0 ( 0 ) | 0 ( 0 ) | 0 ( 0 ) | 0 ( 0 ) | 0 ( 0 ) | 0 ( 0 ) |
| Glyphaea brevis | 0.4 ( 0.9 ) | 0 ( 0 ) | 0 ( 0 ) | 0 ( 0 ) | 0 ( 0 ) | 0 ( 0 ) | 0 ( 0 ) | 0 ( 0 ) | 0 ( 0 ) | 0 ( 0 ) | 0 ( 0 ) | 0 ( 0 ) | 0 ( 0 ) | 0 ( 0 ) | 0 ( 0 ) |
| Greenwayodendron suaveolens | 3.4 ( 3 ) | 1.8 ( 1.6 ) | 1.8 ( 2 ) | 0.2 ( 0.4 ) | 0 ( 0 ) | 0 ( 0 ) | 0 ( 0 ) | 0 ( 0 ) | 0 ( 0 ) | 0 ( 0 ) | 0 ( 0 ) | 0 ( 0 ) | 0 ( 0 ) | 0 ( 0 ) | 0 ( 0 ) |
| Grewia oligoneura | 0.2 ( 0.4 ) | 0 ( 0 ) | 0 ( 0 ) | 0 ( 0 ) | 0 ( 0 ) | 0 ( 0 ) | 0 ( 0 ) | 0 ( 0 ) | 0 ( 0 ) | 0 ( 0 ) | 0 ( 0 ) | 0 ( 0 ) | 0 ( 0 ) | 0 ( 0 ) | 0 ( 0 ) |
| Grewia sp. | 0.4 ( 0.5 ) | 0 ( 0 ) | 0 ( 0 ) | 0 ( 0 ) | 0 ( 0 ) | 0 ( 0 ) | 0 ( 0 ) | 0 ( 0 ) | 0 ( 0 ) | 0 ( 0 ) | 0 ( 0 ) | 0 ( 0 ) | 0 ( 0 ) | 0 ( 0 ) | 0 ( 0 ) |
| Grossera multinervis | 2 ( 3.9 ) | 1.2 ( 2.7 ) | 0 ( 0 ) | 0 ( 0 ) | 0 ( 0 ) | 0 ( 0 ) | 0 ( 0 ) | 0 ( 0 ) | 0 ( 0 ) | 0 ( 0 ) | 0 ( 0 ) | 0 ( 0 ) | 0 ( 0 ) | 0 ( 0 ) | 0 ( 0 ) |
| Guarea cedrata | 0.2 ( 0.4 ) | 0 ( 0 ) | 0 ( 0 ) | 0 ( 0 ) | 0 ( 0 ) | 0.4 ( 0.9 ) | 0.2 ( 0.4 ) | 0 ( 0 ) | 0 ( 0 ) | 0 ( 0 ) | 0 ( 0 ) | 0 ( 0 ) | 0 ( 0 ) | 0 ( 0 ) | 0 ( 0 ) |
| Guarea thompsonii | 4 ( 4.4 ) | 3 ( 1.2 ) | 2 ( 1.4 ) | 0.6 ( 0.9 ) | 0 ( 0 ) | 0 ( 0 ) | 0 ( 0 ) | 0 ( 0 ) | 0 ( 0 ) | 0 ( 0 ) | 0 ( 0 ) | 0 ( 0 ) | 0 ( 0 ) | 0 ( 0 ) | 0 ( 0 ) |
| Hallea stipulosa | 0.2 ( 0.4 ) | 0 ( 0 ) | 0 ( 0 ) | 0 ( 0 ) | 0 ( 0 ) | 0 ( 0 ) | 0 ( 0 ) | 0 ( 0 ) | 0 ( 0 ) | 0 ( 0 ) | 0 ( 0 ) | 0 ( 0 ) | 0 ( 0 ) | 0 ( 0 ) | 0 ( 0 ) |
| Hannoa klaineana | 0 ( 0 ) | 0.2 ( 0.4 ) | 0 ( 0 ) | 0 ( 0 ) | 0 ( 0 ) | 0 ( 0 ) | 0 ( 0 ) | 0 ( 0 ) | 0 ( 0 ) | 0 ( 0 ) | 0 ( 0 ) | 0 ( 0 ) | 0 ( 0 ) | 0 ( 0 ) | 0 ( 0 ) |
| Heisteria parvifolia | 0.2 ( 0.4 ) | 0 ( 0 ) | 0 ( 0 ) | 0 ( 0 ) | 0 ( 0 ) | 0 ( 0 ) | 0 ( 0 ) | 0 ( 0 ) | 0 ( 0 ) | 0 ( 0 ) | 0 ( 0 ) | 0 ( 0 ) | 0 ( 0 ) | 0 ( 0 ) | 0 ( 0 ) |
| Homalium africanum | 0.4 ( 0.9 ) | 0 ( 0 ) | 0 ( 0 ) | 0 ( 0 ) | 0 ( 0 ) | 0 ( 0 ) | 0 ( 0 ) | 0 ( 0 ) | 0 ( 0 ) | 0 ( 0 ) | 0 ( 0 ) | 0 ( 0 ) | 0 ( 0 ) | 0 ( 0 ) | 0 ( 0 ) |
| Homalium longistylum | 0 ( 0 ) | 0 ( 0 ) | 0.2 ( 0.4 ) | 0 ( 0 ) | 0 ( 0 ) | 0 ( 0 ) | 0 ( 0 ) | 0 ( 0 ) | 0 ( 0 ) | 0 ( 0 ) | 0 ( 0 ) | 0 ( 0 ) | 0 ( 0 ) | 0 ( 0 ) | 0 ( 0 ) |
| Hua gabonii | 0.2 ( 0.4 ) | 0 ( 0 ) | 0 ( 0 ) | 0 ( 0 ) | 0 ( 0 ) | 0 ( 0 ) | 0 ( 0 ) | 0 ( 0 ) | 0 ( 0 ) | 0 ( 0 ) | 0 ( 0 ) | 0 ( 0 ) | 0 ( 0 ) | 0 ( 0 ) | 0 ( 0 ) |
| Inconnu | 9 ( 5.6 ) | 3.2 ( 2.2 ) | 2 ( 2.3 ) | 1.4 ( 1.3 ) | 0.8 ( 0.8 ) | 0.2 ( 0.4 ) | 0 ( 0 ) | 0 ( 0 ) | 0.2 ( 0.4 ) | 0 ( 0 ) | 0 ( 0 ) | 0 ( 0 ) | 0 ( 0 ) | 0 ( 0 ) | 0 ( 0 ) |
| Irvingia gabonensis | 0 ( 0 ) | 0 ( 0 ) | 0 ( 0 ) | 0 ( 0 ) | 0 ( 0 ) | 0 ( 0 ) | 0.2 ( 0.4 ) | 0.2 ( 0.4 ) | 0.2 ( 0.4 ) | 0 ( 0 ) | 0.2 ( 0.4 ) | 0 ( 0 ) | 0 ( 0 ) | 0 ( 0 ) | 0 ( 0 ) |
| Irvingia grandifolia | 0.2 ( 0.4 ) | 0.2 ( 0.4 ) | 0.2 ( 0.4 ) | 0 ( 0 ) | 0.2 ( 0.4 ) | 0.2 ( 0.4 ) | 0 ( 0 ) | 0 ( 0 ) | 0 ( 0 ) | 0 ( 0 ) | 0.2 ( 0.4 ) | 0 ( 0 ) | 0 ( 0 ) | 0 ( 0 ) | 0 ( 0 ) |
| Isolona thonneri | 0.2 ( 0.4 ) | 0 ( 0 ) | 0 ( 0 ) | 0 ( 0 ) | 0 ( 0 ) | 0 ( 0 ) | 0 ( 0 ) | 0 ( 0 ) | 0 ( 0 ) | 0 ( 0 ) | 0 ( 0 ) | 0 ( 0 ) | 0 ( 0 ) | 0 ( 0 ) | 0 ( 0 ) |
| Klainedoxa gabonensis | 0.4 ( 0.5 ) | 0.2 ( 0.4 ) | 0.2 ( 0.4 ) | 0.2 ( 0.4 ) | 0 ( 0 ) | 0 ( 0 ) | 0.4 ( 0.5 ) | 0.2 ( 0.4 ) | 0 ( 0 ) | 0 ( 0 ) | 0 ( 0 ) | 0 ( 0 ) | 0 ( 0 ) | 0 ( 0 ) | 0 ( 0 ) |
| Lovoa trichilioides | 0.6 ( 0.9 ) | 0 ( 0 ) | 0 ( 0 ) | 0 ( 0 ) | 0.2 ( 0.4 ) | 0 ( 0 ) | 0 ( 0 ) | 0 ( 0 ) | 0 ( 0 ) | 0 ( 0 ) | 0 ( 0 ) | 0 ( 0 ) | 0 ( 0 ) | 0 ( 0 ) | 0 ( 0 ) |
| Macaranga monandra | 0.2 ( 0.4 ) | 0.2 ( 0.4 ) | 0 ( 0 ) | 0 ( 0 ) | 0 ( 0 ) | 0 ( 0 ) | 0 ( 0 ) | 0 ( 0 ) | 0 ( 0 ) | 0 ( 0 ) | 0 ( 0 ) | 0 ( 0 ) | 0 ( 0 ) | 0 ( 0 ) | 0 ( 0 ) |
| Massularia acuminata | 0.8 ( 0.8 ) | 0 ( 0 ) | 0 ( 0 ) | 0 ( 0 ) | 0 ( 0 ) | 0 ( 0 ) | 0 ( 0 ) | 0 ( 0 ) | 0 ( 0 ) | 0 ( 0 ) | 0 ( 0 ) | 0 ( 0 ) | 0 ( 0 ) | 0 ( 0 ) | 0 ( 0 ) |
| Microdesmis sp. | 4.2 ( 9.4 ) | 3.8 ( 8.5 ) | 0 ( 0 ) | 0 ( 0 ) | 0.2 ( 0.4 ) | 0 ( 0 ) | 0 ( 0 ) | 0 ( 0 ) | 0 ( 0 ) | 0 ( 0 ) | 0 ( 0 ) | 0 ( 0 ) | 0 ( 0 ) | 0 ( 0 ) | 0 ( 0 ) |
| Microdesmis yafungana | 11.4 ( 25.5 ) | 3.2 ( 7.2 ) | 0.4 ( 0.9 ) | 0 ( 0 ) | 0 ( 0 ) | 0 ( 0 ) | 0 ( 0 ) | 0 ( 0 ) | 0 ( 0 ) | 0 ( 0 ) | 0 ( 0 ) | 0 ( 0 ) | 0 ( 0 ) | 0 ( 0 ) | 0 ( 0 ) |
| Milicia excelsa | 0 ( 0 ) | 0.2 ( 0.4 ) | 0 ( 0 ) | 0 ( 0 ) | 0 ( 0 ) | 0 ( 0 ) | 0 ( 0 ) | 0 ( 0 ) | 0 ( 0 ) | 0 ( 0 ) | 0 ( 0 ) | 0 ( 0 ) | 0 ( 0 ) | 0 ( 0 ) | 0 ( 0 ) |
| Millettia drastica | 0.4 ( 0.5 ) | 0.2 ( 0.4 ) | 0.2 ( 0.4 ) | 0 ( 0 ) | 0 ( 0 ) | 0 ( 0 ) | 0 ( 0 ) | 0 ( 0 ) | 0 ( 0 ) | 0 ( 0 ) | 0 ( 0 ) | 0 ( 0 ) | 0 ( 0 ) | 0 ( 0 ) | 0 ( 0 ) |
| Millettia dubia | 0.2 ( 0.4 ) | 0 ( 0 ) | 0 ( 0 ) | 0 ( 0 ) | 0 ( 0 ) | 0 ( 0 ) | 0 ( 0 ) | 0 ( 0 ) | 0 ( 0 ) | 0 ( 0 ) | 0 ( 0 ) | 0 ( 0 ) | 0 ( 0 ) | 0 ( 0 ) | 0 ( 0 ) |
| Millettia hylobia | 0.2 ( 0.4 ) | 0 ( 0 ) | 0 ( 0 ) | 0 ( 0 ) | 0 ( 0 ) | 0 ( 0 ) | 0 ( 0 ) | 0 ( 0 ) | 0 ( 0 ) | 0 ( 0 ) | 0 ( 0 ) | 0 ( 0 ) | 0 ( 0 ) | 0 ( 0 ) | 0 ( 0 ) |
| Monodora angolensis | 0.4 ( 0.9 ) | 0.2 ( 0.4 ) | 0.2 ( 0.4 ) | 0 ( 0 ) | 0 ( 0 ) | 0 ( 0 ) | 0 ( 0 ) | 0 ( 0 ) | 0 ( 0 ) | 0 ( 0 ) | 0 ( 0 ) | 0 ( 0 ) | 0 ( 0 ) | 0 ( 0 ) | 0 ( 0 ) |
| Monodora myristica | 0.6 ( 0.9 ) | 0 ( 0 ) | 0 ( 0 ) | 0 ( 0 ) | 0 ( 0 ) | 0 ( 0 ) | 0 ( 0 ) | 0 ( 0 ) | 0 ( 0 ) | 0 ( 0 ) | 0 ( 0 ) | 0 ( 0 ) | 0 ( 0 ) | 0 ( 0 ) | 0 ( 0 ) |
| Musanga cecropioides | 0.4 ( 0.5 ) | 0.4 ( 0.5 ) | 1.6 ( 1.5 ) | 0.4 ( 0.5 ) | 0 ( 0 ) | 0 ( 0 ) | 0 ( 0 ) | 0 ( 0 ) | 0 ( 0 ) | 0 ( 0 ) | 0 ( 0 ) | 0 ( 0 ) | 0 ( 0 ) | 0 ( 0 ) | 0 ( 0 ) |
| Myrianthus arboreus | 0.6 ( 0.9 ) | 0 ( 0 ) | 0 ( 0 ) | 0 ( 0 ) | 0 ( 0 ) | 0 ( 0 ) | 0 ( 0 ) | 0 ( 0 ) | 0 ( 0 ) | 0 ( 0 ) | 0 ( 0 ) | 0 ( 0 ) | 0 ( 0 ) | 0 ( 0 ) | 0 ( 0 ) |
| Omphalocarpum lecomteanum | 0 ( 0 ) | 0 ( 0 ) | 0 ( 0 ) | 0 ( 0 ) | 0 ( 0 ) | 0 ( 0 ) | 0 ( 0 ) | 0 ( 0 ) | 0 ( 0 ) | 0.2 ( 0.4 ) | 0 ( 0 ) | 0 ( 0 ) | 0 ( 0 ) | 0 ( 0 ) | 0 ( 0 ) |
| Oncoba glauca | 0.2 ( 0.4 ) | 0 ( 0 ) | 0 ( 0 ) | 0 ( 0 ) | 0 ( 0 ) | 0 ( 0 ) | 0 ( 0 ) | 0 ( 0 ) | 0 ( 0 ) | 0 ( 0 ) | 0 ( 0 ) | 0 ( 0 ) | 0 ( 0 ) | 0 ( 0 ) | 0 ( 0 ) |
| Ongokea gore | 0 ( 0 ) | 0 ( 0 ) | 0.2 ( 0.4 ) | 0.2 ( 0.4 ) | 0.4 ( 0.9 ) | 0.2 ( 0.4 ) | 0 ( 0 ) | 0.2 ( 0.4 ) | 0 ( 0 ) | 0 ( 0 ) | 0 ( 0 ) | 0 ( 0 ) | 0 ( 0 ) | 0 ( 0 ) | 0 ( 0 ) |
| Oxyanthus speciosusÂ | 0.2 ( 0.4 ) | 0 ( 0 ) | 0 ( 0 ) | 0 ( 0 ) | 0 ( 0 ) | 0 ( 0 ) | 0 ( 0 ) | 0 ( 0 ) | 0 ( 0 ) | 0 ( 0 ) | 0 ( 0 ) | 0 ( 0 ) | 0 ( 0 ) | 0 ( 0 ) | 0 ( 0 ) |
| Pancovia harmsiana | 10 ( 11.3 ) | 3 ( 5.6 ) | 0 ( 0 ) | 0 ( 0 ) | 0 ( 0 ) | 0 ( 0 ) | 0 ( 0 ) | 0 ( 0 ) | 0 ( 0 ) | 0 ( 0 ) | 0 ( 0 ) | 0 ( 0 ) | 0 ( 0 ) | 0 ( 0 ) | 0 ( 0 ) |
| Pancovia laurentii | 2.8 ( 2.8 ) | 1.6 ( 1.3 ) | 0.4 ( 0.5 ) | 0 ( 0 ) | 0 ( 0 ) | 0 ( 0 ) | 0 ( 0 ) | 0 ( 0 ) | 0 ( 0 ) | 0 ( 0 ) | 0 ( 0 ) | 0 ( 0 ) | 0 ( 0 ) | 0 ( 0 ) | 0 ( 0 ) |
| Pancovia sp. | 0.2 ( 0.4 ) | 0 ( 0 ) | 0 ( 0 ) | 0 ( 0 ) | 0 ( 0 ) | 0 ( 0 ) | 0 ( 0 ) | 0 ( 0 ) | 0 ( 0 ) | 0 ( 0 ) | 0 ( 0 ) | 0 ( 0 ) | 0 ( 0 ) | 0 ( 0 ) | 0 ( 0 ) |
| Panda oleosa | 3.4 ( 2.5 ) | 4.6 ( 2.5 ) | 2.8 ( 0.8 ) | 3.2 ( 2.7 ) | 2 ( 2.8 ) | 0.6 ( 0.9 ) | 0 ( 0 ) | 0.2 ( 0.4 ) | 0 ( 0 ) | 0 ( 0 ) | 0 ( 0 ) | 0 ( 0 ) | 0 ( 0 ) | 0 ( 0 ) | 0 ( 0 ) |
| Parinari excelsa | 0.2 ( 0.4 ) | 0 ( 0 ) | 0 ( 0 ) | 0 ( 0 ) | 0 ( 0 ) | 0 ( 0 ) | 0 ( 0 ) | 0 ( 0 ) | 0 ( 0 ) | 0 ( 0 ) | 0 ( 0 ) | 0 ( 0 ) | 0 ( 0 ) | 0 ( 0 ) | 0 ( 0 ) |
| Pentaclethra macrophylla | 0.4 ( 0.5 ) | 0.4 ( 0.9 ) | 0.2 ( 0.4 ) | 0.2 ( 0.4 ) | 0 ( 0 ) | 0 ( 0 ) | 0 ( 0 ) | 0 ( 0 ) | 0 ( 0 ) | 0 ( 0 ) | 0 ( 0 ) | 0 ( 0 ) | 0 ( 0 ) | 0 ( 0 ) | 0 ( 0 ) |
| Pericopsis elata | 0 ( 0 ) | 0 ( 0 ) | 0 ( 0 ) | 0 ( 0 ) | 0 ( 0 ) | 0 ( 0 ) | 0 ( 0 ) | 0 ( 0 ) | 0.2 ( 0.4 ) | 0 ( 0 ) | 0 ( 0 ) | 0.2 ( 0.4 ) | 0 ( 0 ) | 0 ( 0 ) | 0 ( 0 ) |
| Petersianthus macrocarpus | 11.4 ( 9.6 ) | 4.8 ( 4.2 ) | 3.6 ( 2.7 ) | 1.8 ( 1.9 ) | 1.4 ( 1.1 ) | 0.6 ( 0.9 ) | 0.2 ( 0.4 ) | 0.2 ( 0.4 ) | 0.2 ( 0.4 ) | 0.2 ( 0.4 ) | 0 ( 0 ) | 0 ( 0 ) | 0 ( 0 ) | 0 ( 0 ) | 0 ( 0 ) |
| Piptadeniastrum africanum | 0.2 ( 0.4 ) | 0.2 ( 0.4 ) | 0 ( 0 ) | 0.2 ( 0.4 ) | 0 ( 0 ) | 0.2 ( 0.4 ) | 0 ( 0 ) | 0 ( 0 ) | 0 ( 0 ) | 0 ( 0 ) | 0 ( 0 ) | 0 ( 0 ) | 0 ( 0 ) | 0 ( 0 ) | 0 ( 0 ) |
| Pleiocarpa pycnantha | 1.2 ( 1.8 ) | 0 ( 0 ) | 0 ( 0 ) | 0 ( 0 ) | 0 ( 0 ) | 0 ( 0 ) | 0 ( 0 ) | 0 ( 0 ) | 0 ( 0 ) | 0 ( 0 ) | 0 ( 0 ) | 0 ( 0 ) | 0 ( 0 ) | 0 ( 0 ) | 0 ( 0 ) |
| Polyalthia suaveolens | 2.2 ( 3 ) | 1.8 ( 2.5 ) | 0.6 ( 0.9 ) | 0.6 ( 0.9 ) | 0 ( 0 ) | 0 ( 0 ) | 0 ( 0 ) | 0 ( 0 ) | 0 ( 0 ) | 0 ( 0 ) | 0 ( 0 ) | 0 ( 0 ) | 0 ( 0 ) | 0 ( 0 ) | 0 ( 0 ) |
| Prioria balsamifera | 3.2 ( 3.5 ) | 0.4 ( 0.9 ) | 0.2 ( 0.4 ) | 0 ( 0 ) | 0 ( 0 ) | 0 ( 0 ) | 0 ( 0 ) | 0 ( 0 ) | 0 ( 0 ) | 0 ( 0 ) | 0 ( 0 ) | 0 ( 0 ) | 0 ( 0 ) | 0 ( 0 ) | 0 ( 0 ) |
| Prioria oxyphylla | 0 ( 0 ) | 0.6 ( 0.9 ) | 0.2 ( 0.4 ) | 0 ( 0 ) | 0 ( 0 ) | 0 ( 0 ) | 0 ( 0 ) | 0 ( 0 ) | 0 ( 0 ) | 0 ( 0 ) | 0 ( 0 ) | 0 ( 0 ) | 0 ( 0 ) | 0 ( 0 ) | 0 ( 0 ) |
| Prioria sp. | 0 ( 0 ) | 0 ( 0 ) | 0 ( 0 ) | 0 ( 0 ) | 0 ( 0 ) | 0 ( 0 ) | 0 ( 0 ) | 0.2 ( 0.4 ) | 0 ( 0 ) | 0 ( 0 ) | 0 ( 0 ) | 0 ( 0 ) | 0 ( 0 ) | 0 ( 0 ) | 0 ( 0 ) |
| Pterocarpus soyauxii | 1 ( 1 ) | 0.2 ( 0.4 ) | 0 ( 0 ) | 0 ( 0 ) | 0 ( 0 ) | 0.2 ( 0.4 ) | 0 ( 0 ) | 0 ( 0 ) | 0 ( 0 ) | 0 ( 0 ) | 0 ( 0 ) | 0 ( 0 ) | 0 ( 0 ) | 0 ( 0 ) | 0 ( 0 ) |
| Pterygota bequaertii | 0 ( 0 ) | 0 ( 0 ) | 0 ( 0 ) | 0.2 ( 0.4 ) | 0 ( 0 ) | 0 ( 0 ) | 0 ( 0 ) | 0 ( 0 ) | 0 ( 0 ) | 0 ( 0 ) | 0 ( 0 ) | 0 ( 0 ) | 0 ( 0 ) | 0 ( 0 ) | 0 ( 0 ) |
| Pycnanthus angolensis | 2.6 ( 1.5 ) | 0.4 ( 0.5 ) | 0 ( 0 ) | 0 ( 0 ) | 0 ( 0 ) | 0 ( 0 ) | 0 ( 0 ) | 0 ( 0 ) | 0 ( 0 ) | 0 ( 0 ) | 0 ( 0 ) | 0 ( 0 ) | 0 ( 0 ) | 0 ( 0 ) | 0 ( 0 ) |
| Pycnanthus marshalianus | 0 ( 0 ) | 0 ( 0 ) | 0 ( 0 ) | 0 ( 0 ) | 0 ( 0 ) | 0 ( 0 ) | 0.2 ( 0.4 ) | 0 ( 0 ) | 0 ( 0 ) | 0 ( 0 ) | 0 ( 0 ) | 0 ( 0 ) | 0 ( 0 ) | 0 ( 0 ) | 0 ( 0 ) |
| Quassia undulata | 0.6 ( 0.9 ) | 0.6 ( 0.9 ) | 0 ( 0 ) | 0.2 ( 0.4 ) | 0 ( 0 ) | 0 ( 0 ) | 0 ( 0 ) | 0 ( 0 ) | 0 ( 0 ) | 0 ( 0 ) | 0 ( 0 ) | 0 ( 0 ) | 0 ( 0 ) | 0 ( 0 ) | 0 ( 0 ) |
| Randia africana | 0.2 ( 0.4 ) | 0 ( 0 ) | 0 ( 0 ) | 0 ( 0 ) | 0 ( 0 ) | 0 ( 0 ) | 0 ( 0 ) | 0 ( 0 ) | 0 ( 0 ) | 0 ( 0 ) | 0 ( 0 ) | 0 ( 0 ) | 0 ( 0 ) | 0 ( 0 ) | 0 ( 0 ) |
| Ricinodendron heudelotii | 0 ( 0 ) | 0 ( 0 ) | 0 ( 0 ) | 0.2 ( 0.4 ) | 0 ( 0 ) | 0 ( 0 ) | 0.2 ( 0.4 ) | 0 ( 0 ) | 0 ( 0 ) | 0 ( 0 ) | 0 ( 0 ) | 0 ( 0 ) | 0 ( 0 ) | 0 ( 0 ) | 0 ( 0 ) |
| Rinorea oblongifolia | 1.4 ( 1.5 ) | 0.8 ( 0.8 ) | 0 ( 0 ) | 0 ( 0 ) | 0 ( 0 ) | 0 ( 0 ) | 0 ( 0 ) | 0 ( 0 ) | 0 ( 0 ) | 0 ( 0 ) | 0 ( 0 ) | 0 ( 0 ) | 0 ( 0 ) | 0 ( 0 ) | 0 ( 0 ) |
| Rinorea sp. | 0.8 ( 1.3 ) | 0.2 ( 0.4 ) | 0 ( 0 ) | 0 ( 0 ) | 0 ( 0 ) | 0 ( 0 ) | 0 ( 0 ) | 0 ( 0 ) | 0 ( 0 ) | 0 ( 0 ) | 0 ( 0 ) | 0 ( 0 ) | 0 ( 0 ) | 0 ( 0 ) | 0 ( 0 ) |
| Rothmannia whitfieldii | 0.2 ( 0.4 ) | 0 ( 0 ) | 0 ( 0 ) | 0 ( 0 ) | 0 ( 0 ) | 0 ( 0 ) | 0 ( 0 ) | 0 ( 0 ) | 0 ( 0 ) | 0 ( 0 ) | 0 ( 0 ) | 0 ( 0 ) | 0 ( 0 ) | 0 ( 0 ) | 0 ( 0 ) |
| Scorodophloeus zenkeri | 7 ( 5.8 ) | 6.2 ( 4.8 ) | 9.4 ( 3.2 ) | 9.2 ( 6.8 ) | 5 ( 3.2 ) | 2.2 ( 2 ) | 0.8 ( 0.8 ) | 0.4 ( 0.5 ) | 0 ( 0 ) | 0 ( 0 ) | 0 ( 0 ) | 0 ( 0 ) | 0 ( 0 ) | 0 ( 0 ) | 0 ( 0 ) |
| Spathodea campanulata | 0.2 ( 0.4 ) | 0 ( 0 ) | 0 ( 0 ) | 0 ( 0 ) | 0 ( 0 ) | 0 ( 0 ) | 0 ( 0 ) | 0 ( 0 ) | 0 ( 0 ) | 0 ( 0 ) | 0 ( 0 ) | 0 ( 0 ) | 0 ( 0 ) | 0 ( 0 ) | 0 ( 0 ) |
| Staudtia kamerunensis | 18.8 ( 8.7 ) | 4.8 ( 3 ) | 1.6 ( 1.1 ) | 0.4 ( 0.5 ) | 0.2 ( 0.4 ) | 0.2 ( 0.4 ) | 0 ( 0 ) | 0.2 ( 0.4 ) | 0 ( 0 ) | 0 ( 0 ) | 0 ( 0 ) | 0 ( 0 ) | 0 ( 0 ) | 0 ( 0 ) | 0 ( 0 ) |
| Sterculia bequaertii | 0 ( 0 ) | 0 ( 0 ) | 0.2 ( 0.4 ) | 0 ( 0 ) | 0 ( 0 ) | 0 ( 0 ) | 0 ( 0 ) | 0 ( 0 ) | 0 ( 0 ) | 0 ( 0 ) | 0 ( 0 ) | 0 ( 0 ) | 0 ( 0 ) | 0 ( 0 ) | 0 ( 0 ) |
| Sterculia sp. | 0 ( 0 ) | 0 ( 0 ) | 0 ( 0 ) | 0.2 ( 0.4 ) | 0 ( 0 ) | 0 ( 0 ) | 0 ( 0 ) | 0 ( 0 ) | 0 ( 0 ) | 0 ( 0 ) | 0 ( 0 ) | 0 ( 0 ) | 0 ( 0 ) | 0 ( 0 ) | 0 ( 0 ) |
| Sterculia trachadantha | 0 ( 0 ) | 0 ( 0 ) | 0 ( 0 ) | 0 ( 0 ) | 0.2 ( 0.4 ) | 0 ( 0 ) | 0.2 ( 0.4 ) | 0 ( 0 ) | 0 ( 0 ) | 0 ( 0 ) | 0 ( 0 ) | 0 ( 0 ) | 0 ( 0 ) | 0 ( 0 ) | 0 ( 0 ) |
| Strombosia grandifolia | 4.6 ( 2.2 ) | 3.6 ( 1.8 ) | 1.6 ( 0.9 ) | 0.2 ( 0.4 ) | 0.2 ( 0.4 ) | 0 ( 0 ) | 0.2 ( 0.4 ) | 0 ( 0 ) | 0 ( 0 ) | 0 ( 0 ) | 0 ( 0 ) | 0 ( 0 ) | 0 ( 0 ) | 0 ( 0 ) | 0 ( 0 ) |
| Strombosia pustulata | 1.6 ( 2.5 ) | 0 ( 0 ) | 0.6 ( 1.3 ) | 0.2 ( 0.4 ) | 0.6 ( 0.9 ) | 0 ( 0 ) | 0 ( 0 ) | 0 ( 0 ) | 0 ( 0 ) | 0 ( 0 ) | 0 ( 0 ) | 0 ( 0 ) | 0 ( 0 ) | 0 ( 0 ) | 0 ( 0 ) |
| Strombosia sp. | 0.2 ( 0.4 ) | 0 ( 0 ) | 0 ( 0 ) | 0 ( 0 ) | 0 ( 0 ) | 0 ( 0 ) | 0 ( 0 ) | 0 ( 0 ) | 0 ( 0 ) | 0 ( 0 ) | 0 ( 0 ) | 0 ( 0 ) | 0 ( 0 ) | 0 ( 0 ) | 0 ( 0 ) |
| Strombosiopsis tetrandra | 0 ( 0 ) | 0 ( 0 ) | 0.4 ( 0.9 ) | 0.4 ( 0.5 ) | 1 ( 1.7 ) | 0.4 ( 0.9 ) | 0.2 ( 0.4 ) | 0.2 ( 0.4 ) | 0 ( 0 ) | 0 ( 0 ) | 0 ( 0 ) | 0 ( 0 ) | 0 ( 0 ) | 0 ( 0 ) | 0 ( 0 ) |
| Symphonia globulifera | 0 ( 0 ) | 0 ( 0 ) | 0.2 ( 0.4 ) | 0 ( 0 ) | 0 ( 0 ) | 0.2 ( 0.4 ) | 0 ( 0 ) | 0 ( 0 ) | 0 ( 0 ) | 0 ( 0 ) | 0 ( 0 ) | 0 ( 0 ) | 0 ( 0 ) | 0 ( 0 ) | 0 ( 0 ) |
| Synsepalum longikineyi | 0.2 ( 0.4 ) | 0 ( 0 ) | 0 ( 0 ) | 0 ( 0 ) | 0 ( 0 ) | 0 ( 0 ) | 0 ( 0 ) | 0 ( 0 ) | 0 ( 0 ) | 0 ( 0 ) | 0 ( 0 ) | 0 ( 0 ) | 0 ( 0 ) | 0 ( 0 ) | 0 ( 0 ) |
| Synsepalum subcordatum | 0.6 ( 0.9 ) | 1.6 ( 0.9 ) | 0.4 ( 0.5 ) | 0.4 ( 0.5 ) | 0 ( 0 ) | 0 ( 0 ) | 0 ( 0 ) | 0 ( 0 ) | 0 ( 0 ) | 0 ( 0 ) | 0 ( 0 ) | 0 ( 0 ) | 0 ( 0 ) | 0 ( 0 ) | 0 ( 0 ) |
| Tabernaemontana crassa | 1.4 ( 0.5 ) | 0 ( 0 ) | 0 ( 0 ) | 0 ( 0 ) | 0 ( 0 ) | 0 ( 0 ) | 0 ( 0 ) | 0 ( 0 ) | 0 ( 0 ) | 0 ( 0 ) | 0 ( 0 ) | 0 ( 0 ) | 0 ( 0 ) | 0 ( 0 ) | 0 ( 0 ) |
| Tessmannia africana | 0 ( 0 ) | 0 ( 0 ) | 0.2 ( 0.4 ) | 0 ( 0 ) | 0 ( 0 ) | 0 ( 0 ) | 0.2 ( 0.4 ) | 0 ( 0 ) | 0 ( 0 ) | 0 ( 0 ) | 0 ( 0 ) | 0 ( 0 ) | 0 ( 0 ) | 0 ( 0 ) | 0 ( 0 ) |
| Tetrapleura tetraptera | 0.2 ( 0.4 ) | 0 ( 0 ) | 0 ( 0 ) | 0.2 ( 0.4 ) | 0 ( 0 ) | 0 ( 0 ) | 0 ( 0 ) | 0 ( 0 ) | 0 ( 0 ) | 0 ( 0 ) | 0 ( 0 ) | 0 ( 0 ) | 0 ( 0 ) | 0 ( 0 ) | 0 ( 0 ) |
| Tetrorchidium didymostemon | 0.4 ( 0.9 ) | 0 ( 0 ) | 0 ( 0 ) | 0 ( 0 ) | 0 ( 0 ) | 0 ( 0 ) | 0 ( 0 ) | 0 ( 0 ) | 0 ( 0 ) | 0 ( 0 ) | 0 ( 0 ) | 0 ( 0 ) | 0 ( 0 ) | 0 ( 0 ) | 0 ( 0 ) |
| Thomandersia sp. | 0.2 ( 0.4 ) | 0 ( 0 ) | 0 ( 0 ) | 0 ( 0 ) | 0 ( 0 ) | 0 ( 0 ) | 0 ( 0 ) | 0 ( 0 ) | 0 ( 0 ) | 0 ( 0 ) | 0 ( 0 ) | 0 ( 0 ) | 0 ( 0 ) | 0 ( 0 ) | 0 ( 0 ) |
| Treculia africana | 0.2 ( 0.4 ) | 0.2 ( 0.4 ) | 0.2 ( 0.4 ) | 0 ( 0 ) | 0 ( 0 ) | 0 ( 0 ) | 0 ( 0 ) | 0 ( 0 ) | 0 ( 0 ) | 0 ( 0 ) | 0 ( 0 ) | 0 ( 0 ) | 0 ( 0 ) | 0 ( 0 ) | 0 ( 0 ) |
| Trichilia gilgiana | 1 ( 1.4 ) | 1.2 ( 2.2 ) | 1 ( 1.2 ) | 0.4 ( 0.5 ) | 0 ( 0 ) | 0 ( 0 ) | 0.2 ( 0.4 ) | 0 ( 0 ) | 0 ( 0 ) | 0 ( 0 ) | 0 ( 0 ) | 0 ( 0 ) | 0 ( 0 ) | 0 ( 0 ) | 0 ( 0 ) |
| Trichilia monadelpha | 0.6 ( 0.9 ) | 0.6 ( 0.9 ) | 0.4 ( 0.5 ) | 0 ( 0 ) | 0 ( 0 ) | 0 ( 0 ) | 0 ( 0 ) | 0 ( 0 ) | 0 ( 0 ) | 0 ( 0 ) | 0 ( 0 ) | 0 ( 0 ) | 0 ( 0 ) | 0 ( 0 ) | 0 ( 0 ) |
| Trichilia prieuriana | 1.6 ( 1.8 ) | 1.2 ( 0.8 ) | 0.4 ( 0.9 ) | 0 ( 0 ) | 0.2 ( 0.4 ) | 0 ( 0 ) | 0 ( 0 ) | 0 ( 0 ) | 0 ( 0 ) | 0 ( 0 ) | 0 ( 0 ) | 0 ( 0 ) | 0 ( 0 ) | 0 ( 0 ) | 0 ( 0 ) |
| Trichilia rubescens | 1.2 ( 1.3 ) | 0 ( 0 ) | 0 ( 0 ) | 0 ( 0 ) | 0 ( 0 ) | 0 ( 0 ) | 0 ( 0 ) | 0 ( 0 ) | 0 ( 0 ) | 0 ( 0 ) | 0 ( 0 ) | 0 ( 0 ) | 0 ( 0 ) | 0 ( 0 ) | 0 ( 0 ) |
| Trichilia sp. | 2.4 ( 1.9 ) | 0 ( 0 ) | 0.6 ( 0.9 ) | 0.4 ( 0.9 ) | 0 ( 0 ) | 0 ( 0 ) | 0 ( 0 ) | 0 ( 0 ) | 0 ( 0 ) | 0 ( 0 ) | 0 ( 0 ) | 0 ( 0 ) | 0 ( 0 ) | 0 ( 0 ) | 0 ( 0 ) |
| Trichilia tessmannii | 0.2 ( 0.4 ) | 0 ( 0 ) | 0 ( 0 ) | 0 ( 0 ) | 0 ( 0 ) | 0 ( 0 ) | 0 ( 0 ) | 0 ( 0 ) | 0 ( 0 ) | 0 ( 0 ) | 0 ( 0 ) | 0 ( 0 ) | 0 ( 0 ) | 0 ( 0 ) | 0 ( 0 ) |
| Trichilia welwitschii | 0.6 ( 0.9 ) | 0.2 ( 0.4 ) | 0.2 ( 0.4 ) | 0 ( 0 ) | 0 ( 0 ) | 0 ( 0 ) | 0 ( 0 ) | 0 ( 0 ) | 0 ( 0 ) | 0 ( 0 ) | 0 ( 0 ) | 0 ( 0 ) | 0 ( 0 ) | 0 ( 0 ) | 0 ( 0 ) |
| Tridesmostemon claessensii | 2.2 ( 3.9 ) | 2.4 ( 3.8 ) | 1.8 ( 2.4 ) | 1.4 ( 1.7 ) | 0.2 ( 0.4 ) | 0.2 ( 0.4 ) | 0 ( 0 ) | 0 ( 0 ) | 0 ( 0 ) | 0 ( 0 ) | 0 ( 0 ) | 0 ( 0 ) | 0 ( 0 ) | 0 ( 0 ) | 0 ( 0 ) |
| Tridesmostemon omphalocarpoides | 3.6 ( 4.5 ) | 1.6 ( 3 ) | 1.2 ( 1.8 ) | 0.6 ( 0.9 ) | 0.4 ( 0.9 ) | 0.4 ( 0.5 ) | 0 ( 0 ) | 0.2 ( 0.4 ) | 0.2 ( 0.4 ) | 0 ( 0 ) | 0 ( 0 ) | 0 ( 0 ) | 0 ( 0 ) | 0 ( 0 ) | 0 ( 0 ) |
| Tridesmostemon sp. | 0 ( 0 ) | 0 ( 0 ) | 0 ( 0 ) | 0.2 ( 0.4 ) | 0 ( 0 ) | 0 ( 0 ) | 0 ( 0 ) | 0 ( 0 ) | 0 ( 0 ) | 0 ( 0 ) | 0 ( 0 ) | 0 ( 0 ) | 0 ( 0 ) | 0 ( 0 ) | 0 ( 0 ) |
| Trilepisium madagascariense | 2 ( 1.9 ) | 0.8 ( 1.8 ) | 1 ( 1.7 ) | 0.4 ( 0.9 ) | 0.2 ( 0.4 ) | 0 ( 0 ) | 0 ( 0 ) | 0 ( 0 ) | 0.2 ( 0.4 ) | 0 ( 0 ) | 0 ( 0 ) | 0 ( 0 ) | 0 ( 0 ) | 0 ( 0 ) | 0 ( 0 ) |
| Turraeanthus africanus | 3.6 ( 4.2 ) | 1.6 ( 1.3 ) | 0.4 ( 0.5 ) | 0.6 ( 0.9 ) | 0.4 ( 0.9 ) | 0 ( 0 ) | 0 ( 0 ) | 0 ( 0 ) | 0 ( 0 ) | 0 ( 0 ) | 0 ( 0 ) | 0.2 ( 0.4 ) | 0 ( 0 ) | 0 ( 0 ) | 0 ( 0 ) |
| Uapaca guineensis | 0 ( 0 ) | 0.2 ( 0.4 ) | 0 ( 0 ) | 0 ( 0 ) | 0 ( 0 ) | 0.2 ( 0.4 ) | 0 ( 0 ) | 0 ( 0 ) | 0 ( 0 ) | 0 ( 0 ) | 0 ( 0 ) | 0 ( 0 ) | 0 ( 0 ) | 0 ( 0 ) | 0 ( 0 ) |
| Vepris sp. | 0 ( 0 ) | 0 ( 0 ) | 0.2 ( 0.4 ) | 0 ( 0 ) | 0 ( 0 ) | 0 ( 0 ) | 0 ( 0 ) | 0 ( 0 ) | 0 ( 0 ) | 0 ( 0 ) | 0 ( 0 ) | 0 ( 0 ) | 0 ( 0 ) | 0 ( 0 ) | 0 ( 0 ) |
| Vitex congolensis | 0 ( 0 ) | 0 ( 0 ) | 0 ( 0 ) | 0 ( 0 ) | 0 ( 0 ) | 0.2 ( 0.4 ) | 0 ( 0 ) | 0 ( 0 ) | 0 ( 0 ) | 0 ( 0 ) | 0 ( 0 ) | 0 ( 0 ) | 0 ( 0 ) | 0 ( 0 ) | 0 ( 0 ) |
| Vitex sp. | 0.2 ( 0.4 ) | 0.2 ( 0.4 ) | 0.2 ( 0.4 ) | 0 ( 0 ) | 0 ( 0 ) | 0 ( 0 ) | 0 ( 0 ) | 0 ( 0 ) | 0 ( 0 ) | 0 ( 0 ) | 0 ( 0 ) | 0 ( 0 ) | 0 ( 0 ) | 0 ( 0 ) | 0 ( 0 ) |
| Vitex welwitschii | 1 ( 1.7 ) | 0.8 ( 0.4 ) | 0.4 ( 0.5 ) | 0.2 ( 0.4 ) | 0 ( 0 ) | 0 ( 0 ) | 0 ( 0 ) | 0 ( 0 ) | 0 ( 0 ) | 0 ( 0 ) | 0 ( 0 ) | 0 ( 0 ) | 0 ( 0 ) | 0 ( 0 ) | 0 ( 0 ) |
| Xylopia africana | 0 ( 0 ) | 0 ( 0 ) | 0 ( 0 ) | 0.2 ( 0.4 ) | 0 ( 0 ) | 0 ( 0 ) | 0 ( 0 ) | 0 ( 0 ) | 0 ( 0 ) | 0 ( 0 ) | 0 ( 0 ) | 0 ( 0 ) | 0 ( 0 ) | 0 ( 0 ) | 0 ( 0 ) |
| Xylopia phloiodora | 0 ( 0 ) | 0.2 ( 0.4 ) | 0 ( 0 ) | 0 ( 0 ) | 0 ( 0 ) | 0 ( 0 ) | 0 ( 0 ) | 0 ( 0 ) | 0 ( 0 ) | 0 ( 0 ) | 0 ( 0 ) | 0 ( 0 ) | 0 ( 0 ) | 0 ( 0 ) | 0 ( 0 ) |

**Leaf area index and light availability**

In order to identify differences in light availability in the forests of Yangambi and Yoko, hemispherical images were collected to assess LAI. Hemispherical photographs were acquired in June 2010, at 1m above ground level using a Sigma 4.5mm f/2.8 EX DC HSM Circular Fisheye mounted on a Nikon D300 camera looking upward, level and oriented to magnetic north. Images were recorded with at a resolution of 12 MPixels, ‘fine’ image compression and an ISO 200 underexposing 3 stops. Understory present in the direct neighbourhood of the measurement location was cut down. Data was collected between 7:00 and 9:00 a.m. to avoid overexposure and to reduce sun reflections. At each site, images were acquired in a large grid of 9 hectare, using a gridded sampling scheme with measurement locations every 25 m. LAI was calculated using a histogram analysis based on entropy crossover method [68].

Hemispherical photography based LAI assessment in Yoko and Yangambi old-growth forests revealed no difference. In Yoko, a similar LAI of 4.1 ± 0.8 is found compared with 3.9 ± 0.7 in Yangambi. Additionally, the absolute range of LAI was similar at both sites ranging between 2.6 – 7.4 in Yoko and between 2.8 – 6.9 in Yangambi. The large variability does indicate a variable canopy structure at both sites. Light availability at these two sites is thus similar, although differences in vertical forest structure and occurrence of natural disturbance can induce strong local differences in light availability. Accordingly, a similar abundance of light-demanding and shade tolerant species is present at Yoko and Yangambi.

**Height-diameter model selection**

**S4 Table.** Tested height-diameter function forms where H is height, D is diameter and a, b and c are constant coefficients to be estimated.

| **Equation name** | **Function** | **Reference** |
| --- | --- | --- |
| Power | $H=aD^{b}$ | Huxley 1932 [69]; Enquist 2002 [70] |
| 2-parameter exponential | $H=a(1-e^{-bD})$ | Meyer 1940 [71] |
| 3-parameter exponential | $H=a-b.e^{-cD}$ | Pinheiro et al. 1994 [72] ; Fang & Bailey 1998 [73] |
| Gompertz | $H=ae^{-be^{-cD}}$ | Winsor 1932 [74]; Richards 1959 [75] |
| Logistic | $H=a/(1+be^{-cD})$ | Winsor 1932 [74]; Richards 1959 [75] |
| Weibull | $H=a(1-e^{-bD^{c}})$ | Yang et al. 1978 [76] |

**S5 Table.** Parameterization for the different models functions in Table S4 for Yangambi.

| **Models** | **a** | **b** | **c** | **RSE** | **AIC** |
| --- | --- | --- | --- | --- | --- |
| Power | 3.923 (0.368) | 0.475 (0.025) | na | 4.264 | 1089 |
| 2-parameter exponential | 32.300 (1.287) | 0.034 (0.003) | na | 4.279 | 1090 |
| 3-parameter exponential | 36.358 (2.930) | 31.659 (2.166) | 0.022 (0.005) | 4.221 | 1085 |
| Gompertz | 33.694 (1.886) | 1.561 (0.111) | 0.036 (0.005) | 4.226 | 1086 |
| Logistic | 32.198 (1.456) | 2.965 (0.321) | 0.052 (0.006) | 4.239 | 1087 |
| Weibull | 41.051 (7.880) | 0.054 (0.008) | 0.740 (0.108) | 4.223 | 1085 |

**S6 Table.** Parameterization for the different models functions in Table S4 for Yoko.

| **Models** | **a** | **b** | **c** | **RSE** |  | **AIC** |
| --- | --- | --- | --- | --- | --- | --- |
| Power | 3.176 (0.208) | 0.544 (0.018) | na | 5.099 |  | 2649 |
| 2-parameter exponential | 38.201 (1.379) | 0.028 (0.002) | na | 5.039 |  | 2639 |
| 3-parameter exponential | 42.502 (2.686) | 39.147 (2.158) | 0.020 (0.003) | 4.962 |  | 2627 |
| Gompertz | 38.431 (1.600) | 1.837 (0.075) | 0.036 (0.003) | 4.964 |  | 2627 |
| Logistic | 36.701 (1.274) | 3.990 (0.264) | 0.054 (0.004) | 4.965 |  | 2628 |
| Weibull | 44.628 (4.742) | 0.036 (0.065) | 0.842 (0.065) | 5.011 |  | 2635 |

**Supplementary References**

[61] Shannon CE, Weaver W. The Mathematical Theory of Communication. Urbana: University of Illinois Press; 1949.

[62] Simpson EH. Measurement of diversity. Nature. 1949; 163: 688.

[63] Pielou EC. An Introduction to Mathematical Ecology. New York: Wiley; 1969.

[64] Oksanen J. Multivariate analyses of ecological communities in R: vegan tutorial. 2013. http://cc.oulu.fi/~jarioksa/opetus/metodi/vegantutor.pdf. Accessed 12 March 2015.

[65] R Core Team. R: A language and environment for statistical computing. Vienna: R Foundation for Statistical Computing; 2014.

[66] Hill MO, Gauch HG. Detrended correspondence analysis: an improved ordination technique. Vegetation. 1980; 42: 47–58.

[67] Banin L, Feldpausch TR, Phillips OL, Baker TR, J Lloyd, Affum-Baffoe K, et al. What controls tropical forest architecture? Testing environmental, structural and floristic drivers. Global Ecol Biogeogr. 2012; 21: 1179–1190.

[68] Juárez RIN, Rocha HR, Figueira AMS, Goulden ML, Miller SD. An improved estimate of leaf area index based on the histogram analysis of hemispherical photographs. Agr Forest Meteorol. 2009; 149: 920 – 928.

[69] Huxley A. Problems of relative growth. New York: The Dial Press; 1932.

[70] Enquist BJ. Universal scaling in tree and vascular plant allometry: Towards a general quantitative theory linking plant form and function from cells to ecosystems. Tree Physiol. 2002; 22:1045-1064.

[71] Meyer HA. A mathematical expression for height curves. J For. 1940; 38: 415-420.

[72] Pinheiro JC, Bates DM. Lindstrom MJ. Model building in nonlinear mixed effects models. Department of Statistics, Technical Report 931. Madison: University of Wisconsin; 1994.

[73] Fang ZX, Bailey RL. Height-diameter models for tropical forests on Hainan Island in southern China. For Ecol Manage. 1998; 110: 315-327.

[74] Winsor CP. The Gompertz curve as a growth curve. Proc Natl Acad Sci USA. 1932; 18: 1-8.

[75] Richards FJ. A flexible growth function for empirical use. J Exp Bot. 1959; 10: 290-300.

[76] Yang RC, Kozak A, Smith JHG. The potential of Weibull-type functions as flexible growth curves. Can J For Res. 1978; 8: 424-431.
