# Supplementary material for: Aboveground vs. Belowground Carbon Stocks in African Tropical Lowland Rainforest: Drivers and Implications
Source: PLoS One. 2015 Nov 24;10(11):e0143209. doi: 10.1371/journal.pone.0143209 (PMC4657968; doi:10.1371/journal.pone.0143209)
Supplement: S1 Table — (PDF) [file pone.0143209.s004.pdf]

S1 Table. Stand characteristics (Aboveground carbon (AGC); Leaf area index (LAI)), species diversity and location of the two sites.

		Yangambi	YOKO
**Stand characteristics**			
	Wood density (g cm^{-3})	0.63 ± 0.02 (a)	0.64 ± 0.02 (a)
	Stem density (ha^{-1})	419 ± 89 (a)	469 ± 35 (a)
	Basal area (m^2 ha^{-1})	32 ± 3 (a)	34 ± 3 (a)
	AGC (Mg C ha^{-1})	163 ± 19 (a)	191 ± 28 (b)
	LAI	3.9 ± 0.7 (a)	4.1 ± 0.8 (a)
	H-D model ($H = a – b.e^{-cD}$)	a 36.358; b 31.659; c 0.022	a 42.502; b 39.147; c 0.020
**Species diversity**			
	Species richness	76 ± 6 (a)	78 ± 7 (a)
	Pielou's evenness	0.83 ± 0.03 (a)	0.85 ± 0.01 (a)
	Shannon index	3.6 ± 0.2 (a)	3.7 ± 0.1 (a)
	Simpson diversity	0.955 ± 0.007 (a)	0.962 ± 0.003 (b)
**Site location**			
	Latitude (d.dddd)	0.7995	0.2918
	Longitude (d.dddd)	24.5077	25.3113
	Altitude (m asl)	479 ± 13	471 ± 5
	Plot size (ha)	1	0.25 - 1

Regression model: ($H = a – b.e^{-cD}$). a, b and c are the optimized parameters for the individual equations per site, which represent, respectively, the maximum asymptotic height, the difference between minimum and maximum height, and shape of the curve (Banin et al. 2012). For each parameter, significance from t-test is provided between brackets comparing the two sites. Values within a row not sharing a common letter differ significantly ($p > 0.01$).
