# Supplementary material for: Aboveground vs. Belowground Carbon Stocks in African Tropical Lowland Rainforest: Drivers and Implications
Source: PLoS One. 2015 Nov 24;10(11):e0143209. doi: 10.1371/journal.pone.0143209 (PMC4657968; doi:10.1371/journal.pone.0143209)
Supplement: S2 Table — (PDF) [file pone.0143209.s005.pdf]

S2 Table. Average number of stems with standard deviation in brackets of tree species per hectare in Yoko within different diameter classes.

Species/diameter class	10-20	20-30	30-40	40-50	50-60	60-70	70-80	80-90	90-100	100-110	110-120	120-130	130-140
Afrostyrax lepidophyllus	0.7 (1.2)	0.2 (0.4)	0 (0)	0.2 (0.4)	0 (0)	0 (0)	0 (0)	0 (0)	0 (0)	0 (0)	0 (0)	0 (0)	0 (0)
Aidia micrantha	18.5 (6.2)	0.5 (0.8)	0 (0)	0 (0)	0 (0)	0 (0)	0 (0)	0 (0)	0 (0)	0 (0)	0 (0)	0 (0)	0 (0)
Albizia adianthifolia	1 (1.3)	0.8 (0.8)	0 (0)	0.2 (0.4)	0 (0)	0 (0)	0 (0)	0 (0)	0 (0)	0 (0)	0 (0)	0 (0)	0 (0)
Albizia ferruginea	0 (0)	0.2 (0.4)	0 (0)	0 (0)	0 (0)	0.2 (0.4)	0 (0)	0 (0)	0 (0)	0 (0)	0 (0)	0 (0)	0 (0)
Allanblackia floribunda	1.7 (1.6)	0.5 (1.2)	0.2 (0.4)	0 (0)	0 (0)	0 (0)	0 (0)	0 (0)	0 (0)	0 (0)	0 (0)	0 (0)	0 (0)
Alstonia boonei	0 (0)	0 (0)	0 (0)	0 (0)	0 (0)	0.2 (0.4)	0 (0)	0 (0)	0 (0)	0 (0)	0 (0)	0 (0)	0 (0)
Anonidium mannii	1.5 (1)	2.7 (2.1)	3.3 (0.5)	2.7 (3)	0.2 (0.4)	0.3 (0.5)	0 (0)	0 (0)	0 (0)	0 (0)	0 (0)	0 (0)	0 (0)
Anthonotha fragrans	2.2 (2.4)	0 (0)	0 (0)	0.2 (0.4)	0.2 (0.4)	0.3 (0.5)	0 (0)	0 (0)	0.2 (0.4)	0 (0)	0 (0)	0 (0)	0 (0)
Anthonotha macrophylla	0.5 (0.8)	0 (0)	0 (0)	0 (0)	0 (0)	0 (0)	0 (0)	0 (0)	0 (0)	0 (0)	0 (0)	0 (0)	0 (0)
Antrocaryon nannanii	0 (0)	0.2 (0.4)	0 (0)	0 (0)	0 (0)	0 (0)	0 (0)	0 (0)	0 (0)	0.2 (0.4)	0 (0)	0 (0)	0 (0)
Balanites wilsoniana	0.2 (0.4)	0 (0)	0 (0)	0 (0)	0 (0)	0 (0)	0 (0)	0 (0)	0 (0)	0 (0)	0 (0)	0 (0)	0 (0)
Baphia dewevrei	0.2 (0.4)	0.2 (0.4)	0 (0)	0 (0)	0 (0)	0 (0)	0 (0)	0 (0)	0 (0)	0 (0)	0 (0)	0 (0)	0 (0)
Barteria fistulosa	0.5 (0.8)	0 (0)	0 (0)	0 (0)	0 (0)	0 (0)	0 (0)	0 (0)	0 (0)	0 (0)	0 (0)	0 (0)	0 (0)
Berlinia congolensis	0 (0)	0 (0)	0.2 (0.4)	0 (0)	0 (0)	0 (0)	0 (0)	0 (0)	0 (0)	0 (0)	0 (0)	0 (0)	0 (0)
Blighia unijugata	0.8 (0.8)	0 (0)	0 (0)	0 (0)	0 (0)	0 (0)	0 (0)	0 (0)	0 (0)	0 (0)	0 (0)	0 (0)	0 (0)
Blighia welwitschii	0.2 (0.4)	0 (0)	0.2 (0.4)	0.2 (0.4)	0 (0)	0 (0)	0 (0)	0 (0)	0 (0)	0.2 (0.4)	0 (0)	0 (0)	0 (0)
Bosqueia angolensis	1 (1.3)	0.7 (1.2)	0.5 (0.8)	0 (0)	0 (0)	0 (0)	0 (0)	0 (0)	0 (0)	0 (0)	0 (0)	0 (0)	0 (0)
Breviea sericea	0.3 (0.5)	0 (0)	0 (0)	0 (0)	0 (0)	0 (0)	0 (0)	0 (0)	0 (0)	0 (0)	0 (0)	0 (0)	0 (0)
Breviea sp.	0 (0)	0.2 (0.4)	0 (0)	0 (0)	0 (0)	0 (0)	0 (0)	0 (0)	0 (0)	0 (0)	0 (0)	0 (0)	0 (0)
Canarium schweinfurthii	0 (0)	0 (0)	0.2 (0.4)	0 (0)	0 (0)	0 (0)	0 (0)	0 (0)	0.2 (0.4)	0 (0)	0 (0)	0 (0)	0 (0)
Carapa procera	6.7 (4.9)	0.5 (0.8)	0.2 (0.4)	0 (0)	0 (0)	0 (0)	0 (0)	0 (0)	0 (0)	0 (0)	0 (0)	0 (0)	0 (0)
Celtis mildbraedii	2.3 (2.1)	0.7 (1.2)	1 (0.9)	0.5 (0.5)	1 (0.9)	0.7 (0.8)	0.3 (0.5)	0.2 (0.4)	0 (0)	0 (0)	0.2 (0.4)	0 (0)	0 (0)
Celtis tessmannii	0.2 (0.4)	0 (0)	0 (0)	0.2 (0.4)	0 (0)	0.2 (0.4)	0 (0)	0 (0)	0 (0)	0 (0)	0 (0)	0 (0)	0 (0)
Chlamydocola chlamydantha	0.2 (0.4)	0 (0)	0 (0)	0 (0)	0 (0)	0 (0)	0 (0)	0 (0)	0 (0)	0 (0)	0 (0)	0 (0)	0 (0)
Chrysophyllum africanum	1.5 (1.6)	0 (0)	0.2 (0.4)	0.2 (0.4)	0 (0)	0 (0)	0 (0)	0 (0)	0 (0)	0 (0)	0 (0)	0 (0)	0 (0)
Chrysophyllum lacourtianum	2 (2.6)	0.2 (0.4)	0.2 (0.4)	0 (0)	0 (0)	0 (0)	0 (0)	0 (0)	0 (0)	0 (0)	0 (0)	0 (0)	0 (0)
Chrysophyllum pruniforme	0.5 (0.5)	0.2 (0.4)	0.2 (0.4)	0 (0)	0 (0)	0.2 (0.4)	0 (0)	0.2 (0.4)	0 (0)	0 (0)	0 (0)	0 (0)	0 (0)
Chytranthus carneus	1.7 (1)	0.2 (0.4)	0 (0)	0 (0)	0 (0)	0 (0)	0 (0)	0 (0)	0 (0)	0 (0)	0 (0)	0 (0)	0 (0)

Cleistanthus mildbraedii	3.3 (1.6)	0.7 (0.8)	0 (0)	0.5 (0.8)	0.2 (0.4)	0.2 (0.4)	0 (0)	0 (0)	0 (0)	0 (0)	0 (0)	0 (0)	0 (0)
Cleistanthus ripicola	0.5 (1.2)	0.5 (1.2)	0.2 (0.4)	0 (0)	0 (0)	0.2 (0.4)	0 (0)	0 (0)	0 (0)	0 (0)	0 (0)	0 (0)	0 (0)
Coelocaryon botryoides	2.3 (2)	1 (0.9)	0.3 (0.5)	0 (0)	0 (0)	0 (0)	0 (0)	0 (0)	0 (0)	0 (0)	0 (0)	0 (0)	0 (0)
Cola acuminata	0.3 (0.8)	0 (0)	0 (0)	0 (0)	0 (0)	0 (0)	0 (0)	0 (0)	0 (0)	0 (0)	0 (0)	0 (0)	0 (0)
Cola gigantea	0.8 (1)	0 (0)	0 (0)	0 (0)	0 (0)	0 (0)	0 (0)	0 (0)	0 (0)	0 (0)	0 (0)	0 (0)	0 (0)
Cola griseiflora	23.8 (10.6)	1 (0.9)	0.3 (0.5)	0 (0)	0 (0)	0 (0)	0 (0)	0 (0)	0 (0)	0 (0)	0 (0)	0 (0)	0 (0)
Combretum lokele	0 (0)	0 (0)	0 (0)	0 (0)	0 (0)	0 (0)	0 (0)	0 (0)	0.2 (0.4)	0 (0)	0 (0)	0 (0)	0 (0)
Copaifera mildbraedii	0.5 (0.5)	0 (0)	0 (0)	0 (0)	0 (0)	0 (0)	0 (0)	0 (0)	0 (0)	0 (0)	0 (0)	0 (0)	0 (0)
Cynometra hankei	1.8 (1.2)	1.7 (1.6)	1.3 (0.8)	1.3 (1.5)	0.7 (1.2)	1.2 (1.2)	1 (1.7)	0.8 (1)	0.2 (0.4)	0 (0)	0 (0)	0.2 (0.4)	0 (0)
Cynometra sessiliflora	0 (0)	0.2 (0.4)	0 (0)	0 (0)	0 (0)	0 (0)	0 (0)	0 (0)	0 (0)	0 (0)	0 (0)	0 (0)	0 (0)
Dacryodes edulis	0.5 (0.5)	0 (0)	0.2 (0.4)	0 (0)	0 (0)	0 (0)	0 (0)	0 (0)	0 (0)	0 (0)	0 (0)	0 (0)	0 (0)
Dacryodes osika	0 (0)	0.3 (0.8)	0 (0)	0 (0)	0 (0)	0 (0)	0 (0)	0 (0)	0 (0)	0 (0)	0 (0)	0 (0)	0 (0)
Desplatsia dewevrei	0.2 (0.4)	0 (0)	0 (0)	0 (0)	0 (0)	0 (0)	0 (0)	0 (0)	0 (0)	0 (0)	0 (0)	0 (0)	0 (0)
Dialium corbisieri	0.5 (0.5)	0.3 (0.5)	0 (0)	0 (0)	0.3 (0.5)	0.2 (0.4)	0 (0)	0 (0)	0 (0)	0 (0)	0 (0)	0 (0)	0 (0)
Dialium excelsum	0 (0)	0 (0)	0 (0)	0 (0)	0 (0)	0 (0)	0.2 (0.4)	0 (0)	0 (0)	0 (0)	0 (0)	0 (0)	0 (0)
Dialium pachyphyllum	2.5 (2.7)	0.2 (0.4)	0 (0)	0 (0)	0 (0)	0 (0)	0 (0)	0.2 (0.4)	0 (0)	0 (0)	0 (0)	0 (0)	0 (0)
Dialium soyauxii	0 (0)	0 (0)	0 (0)	0.2 (0.4)	0 (0)	0 (0)	0 (0)	0 (0)	0 (0)	0 (0)	0 (0)	0 (0)	0 (0)
Diogoa zenkeri	4.3 (3.3)	2.5 (2.4)	1 (0.9)	0.2 (0.4)	0 (0)	0 (0)	0 (0)	0 (0)	0 (0)	0 (0)	0 (0)	0 (0)	0 (0)
Diospyros boala	0.3 (0.8)	0 (0)	0 (0)	0 (0)	0 (0)	0 (0)	0 (0)	0 (0)	0 (0)	0 (0)	0 (0)	0 (0)	0 (0)
Diospyros crassiflora	0.2 (0.4)	0 (0)	0 (0)	0 (0)	0 (0)	0 (0)	0 (0)	0 (0)	0 (0)	0 (0)	0 (0)	0 (0)	0 (0)
Diospyros sp.	1.5 (1.9)	0.5 (0.5)	0 (0)	0 (0)	0 (0)	0 (0)	0 (0)	0 (0)	0 (0)	0 (0)	0 (0)	0 (0)	0 (0)
Drypetes gossweileri	1.3 (2)	0.7 (0.8)	0.2 (0.4)	0 (0)	0 (0)	0 (0)	0 (0)	0 (0)	0 (0)	0 (0)	0 (0)	0 (0)	0 (0)
Drypetes likwa	2 (4.9)	0.8 (2)	0.7 (1.6)	0.2 (0.4)	0.2 (0.4)	0 (0)	0 (0)	0 (0)	0 (0)	0 (0)	0 (0)	0 (0)	0 (0)
Drypetes sp.	8.3 (6.4)	3.3 (3)	0.8 (1.3)	0.2 (0.4)	0 (0)	0 (0)	0 (0)	0 (0)	0 (0)	0 (0)	0 (0)	0 (0)	0 (0)
Drypetes spinosodentata	0.5 (1.2)	0.2 (0.4)	0 (0)	0 (0)	0 (0)	0 (0)	0 (0)	0 (0)	0 (0)	0 (0)	0 (0)	0 (0)	0 (0)
Entandrophragma angolense	0 (0)	0.2 (0.4)	0 (0)	0 (0)	0 (0)	0 (0)	0 (0)	0 (0)	0 (0)	0 (0)	0 (0)	0 (0)	0 (0)
Entandrophragma candollei	0.3 (0.5)	0.2 (0.4)	0 (0)	0 (0)	0.2 (0.4)	0 (0)	0 (0)	0 (0)	0 (0)	0 (0)	0 (0)	0 (0)	0.2 (0.4)
Entandrophragma cylindricum	0.5 (0.8)	0 (0)	0 (0)	0 (0)	0 (0)	0 (0)	0 (0)	0 (0)	0 (0)	0 (0)	0 (0)	0 (0)	0 (0)
Entandrophragma utile	0.2 (0.4)	0 (0)	0 (0)	0 (0)	0.2 (0.4)	0 (0)	0 (0)	0 (0)	0 (0)	0 (0)	0 (0)	0 (0)	0 (0)
Eriocoelum microspermum	0.2 (0.4)	0 (0)	0 (0)	0 (0)	0 (0)	0 (0)	0 (0)	0 (0)	0 (0)	0 (0)	0 (0)	0 (0)	0 (0)
Ficus elastica	0 (0)	0 (0)	0 (0)	0 (0)	0 (0)	0.2 (0.4)	0 (0)	0 (0)	0 (0)	0 (0)	0 (0)	0 (0)	0 (0)

Funtumia africana	1.8 (1.9)	0.5 (0.8)	0 (0)	0.2 (0.4)	0 (0)	0 (0)	0 (0)	0 (0)	0 (0)	0 (0)	0 (0)	0 (0)	0 (0)
Garcinia epunctata	0.5 (0.8)	0 (0)	0 (0)	0 (0)	0 (0)	0 (0)	0 (0)	0 (0)	0 (0)	0 (0)	0 (0)	0 (0)	0 (0)
Garcinia punctata	0.3 (0.5)	0.2 (0.4)	0 (0)	0 (0)	0 (0)	0 (0)	0 (0)	0 (0)	0 (0)	0 (0)	0 (0)	0 (0)	0 (0)
Garcinia smeathmannii	0.5 (0.8)	0 (0)	0 (0)	0 (0)	0 (0)	0 (0)	0 (0)	0 (0)	0 (0)	0 (0)	0 (0)	0 (0)	0 (0)
Garcinia sp.	0.2 (0.4)	0 (0)	0 (0)	0 (0)	0 (0)	0 (0)	0 (0)	0 (0)	0 (0)	0 (0)	0 (0)	0 (0)	0 (0)
Grewia oligoneura	0.3 (0.5)	0.2 (0.4)	0.3 (0.5)	0 (0)	0 (0)	0 (0)	0 (0)	0 (0)	0 (0)	0 (0)	0 (0)	0 (0)	0 (0)
Grewia trinervia	1.5 (0.8)	0.5 (0.5)	0.3 (0.5)	0.3 (0.5)	0 (0)	0 (0)	0 (0)	0 (0)	0 (0)	0 (0)	0 (0)	0 (0)	0 (0)
Grossera multinervis	2 (4.9)	0.8 (2)	0.5 (1.2)	0 (0)	0 (0)	0 (0)	0 (0)	0 (0)	0 (0)	0 (0)	0 (0)	0 (0)	0 (0)
Guarea cedrata	0.7 (0.8)	0.2 (0.4)	0 (0)	0 (0)	0 (0)	0.2 (0.4)	0.2 (0.4)	0 (0)	0.2 (0.4)	0 (0)	0 (0)	0 (0)	0 (0)
Guarea laurentii	1.7 (2)	1.5 (2.3)	0.2 (0.4)	0.2 (0.4)	0 (0)	0 (0)	0 (0)	0 (0)	0 (0)	0 (0)	0 (0)	0 (0)	0 (0)
Guarea thompsonii	10.2 (5.2)	2.8 (1.7)	0.3 (0.5)	0.2 (0.4)	0.2 (0.4)	0.3 (0.5)	0 (0)	0 (0)	0 (0)	0.2 (0.4)	0 (0)	0 (0)	0 (0)
Hannoa klaineana	0.5 (0.5)	0.2 (0.4)	0 (0)	0 (0)	0 (0)	0 (0)	0 (0)	0 (0)	0 (0)	0 (0)	0 (0)	0 (0)	0 (0)
Heisteria parvifolia	1.3 (0.8)	1.3 (1)	0.2 (0.4)	0 (0)	0 (0)	0 (0)	0 (0)	0 (0)	0 (0)	0 (0)	0 (0)	0 (0)	0 (0)
Hexalobus crispiflorus	0.2 (0.4)	0 (0)	0 (0)	0 (0)	0.2 (0.4)	0 (0)	0 (0)	0 (0)	0 (0)	0 (0)	0 (0)	0 (0)	0 (0)
Homalium longistylum	0.2 (0.4)	0 (0)	0 (0)	0 (0)	0 (0)	0 (0)	0 (0)	0 (0)	0 (0)	0 (0)	0 (0)	0 (0)	0 (0)
Homalium sp.	0.2 (0.4)	0.2 (0.4)	0 (0)	0 (0)	0 (0)	0 (0)	0 (0)	0 (0)	0 (0)	0 (0)	0 (0)	0 (0)	0 (0)
Hymenostegia pellegrinii	0 (0)	0 (0)	0 (0)	0 (0)	0.2 (0.4)	0 (0)	0 (0)	0 (0)	0 (0)	0 (0)	0 (0)	0 (0)	0 (0)
Irvingia gabonensis	0.8 (1.2)	0 (0)	0 (0)	0 (0)	0 (0)	0 (0)	0 (0)	0 (0)	0 (0)	0 (0)	0 (0)	0 (0)	0 (0)
Irvingia grandifolia	0.2 (0.4)	0 (0)	0 (0)	0 (0)	0 (0)	0 (0)	0 (0)	0 (0)	0 (0)	0 (0)	0 (0)	0 (0)	0 (0)
Julbernardia seretii	3.5 (2.3)	3 (1.3)	3.2 (1.6)	2.2 (1.8)	1 (1.5)	0.3 (0.5)	0.2 (0.4)	0 (0)	0 (0)	0 (0)	0.2 (0.4)	0 (0)	0 (0)
Macaranga monandra	0.8 (1.3)	0.7 (1.2)	0.3 (0.5)	0.3 (0.8)	0.2 (0.4)	0 (0)	0 (0)	0 (0)	0 (0)	0 (0)	0 (0)	0 (0)	0 (0)
Mammea africana	0.2 (0.4)	0 (0)	0 (0)	0 (0)	0 (0)	0 (0)	0 (0)	0 (0)	0 (0)	0 (0)	0 (0)	0 (0)	0 (0)
Manilkara malcolens	0 (0)	0.2 (0.4)	0 (0)	0 (0)	0 (0)	0 (0)	0 (0)	0 (0)	0 (0)	0 (0)	0 (0)	0 (0)	0 (0)
Manilkara sp.	0.5 (0.8)	0.2 (0.4)	0 (0)	0 (0)	0 (0)	0 (0)	0 (0)	0 (0)	0 (0)	0 (0)	0 (0)	0 (0)	0 (0)
Margaritaria discoidea	0.2 (0.4)	0 (0)	0 (0)	0 (0)	0 (0)	0 (0)	0 (0)	0 (0)	0 (0)	0 (0)	0 (0)	0 (0)	0 (0)
Massularia acuminata	0.7 (0.8)	0 (0)	0 (0)	0 (0)	0 (0)	0 (0)	0 (0)	0 (0)	0 (0)	0 (0)	0 (0)	0 (0)	0 (0)
Massularia africana	0.2 (0.4)	0 (0)	0 (0)	0 (0)	0 (0)	0 (0)	0 (0)	0 (0)	0 (0)	0 (0)	0 (0)	0 (0)	0 (0)
Microdesmis yafungana	19.5 (9.4)	5.7 (3.7)	1.2 (1.6)	0.5 (0.5)	0 (0)	0 (0)	0 (0)	0 (0)	0 (0)	0 (0)	0 (0)	0 (0)	0 (0)
Millettia drastica	0.3 (0.5)	0 (0)	0 (0)	0 (0)	0 (0)	0 (0)	0 (0)	0 (0)	0 (0)	0 (0)	0 (0)	0 (0)	0 (0)
Monodora angolensis	0.8 (0.8)	0 (0)	0 (0)	0 (0)	0 (0)	0 (0)	0 (0)	0 (0)	0 (0)	0 (0)	0 (0)	0 (0)	0 (0)
Morinda lucida	0.2 (0.4)	0 (0)	0 (0)	0 (0)	0 (0)	0 (0)	0 (0)	0 (0)	0 (0)	0 (0)	0 (0)	0 (0)	0 (0)

Musanga cecropioides	0.5 (0.5)	0.5 (0.5)	0.3 (0.5)	0.3 (0.5)	0.2 (0.4)	0 (0)	0 (0)	0 (0)	0 (0)	0 (0)	0 (0)	0 (0)	0 (0)
Nesogordonia leplaei	0.5 (1.2)	0 (0)	0 (0)	0 (0)	0 (0)	0 (0)	0 (0)	0 (0)	0 (0)	0 (0)	0 (0)	0 (0)	0 (0)
Nesogordonia sp.	0.5 (0.8)	0.5 (0.5)	0.2 (0.4)	0.3 (0.5)	0.2 (0.4)	0 (0)	0 (0)	0 (0)	0 (0)	0 (0)	0 (0)	0 (0)	0 (0)
Omphalocarpum sp.	0.3 (0.8)	0.2 (0.4)	0 (0)	0 (0)	0 (0)	0 (0)	0 (0)	0 (0)	0 (0)	0 (0)	0 (0)	0 (0)	0 (0)
Oncoba welwitschii	0.5 (0.8)	0.2 (0.4)	0 (0)	0 (0)	0 (0)	0 (0)	0 (0)	0 (0)	0 (0)	0 (0)	0 (0)	0 (0)	0 (0)
Ongokea gore	0 (0)	0 (0)	0 (0)	0 (0)	0 (0)	0.2 (0.4)	0 (0)	0 (0)	0 (0)	0 (0)	0 (0)	0 (0)	0 (0)
Pancovia harmsiana	8.5 (3.5)	0.5 (0.8)	0 (0)	0 (0)	0 (0)	0 (0)	0 (0)	0 (0)	0 (0)	0 (0)	0 (0)	0 (0)	0 (0)
Pancovia laurentii	0.8 (1.2)	0.8 (0.8)	0.5 (0.8)	0 (0)	0 (0)	0 (0)	0 (0)	0 (0)	0 (0)	0 (0)	0 (0)	0 (0)	0 (0)
Panda oleosa	6.5 (3.1)	4.2 (2.1)	2 (0.9)	1 (1.3)	0.7 (0.5)	0 (0)	0 (0)	0 (0)	0 (0)	0 (0)	0 (0)	0 (0)	0 (0)
Paramacrolobium coeruleum	0.3 (0.8)	0.2 (0.4)	0.2 (0.4)	0 (0)	0 (0)	0 (0)	0 (0)	0 (0)	0 (0)	0 (0)	0 (0)	0 (0)	0 (0)
Parkia bicolor	0 (0)	0.2 (0.4)	0 (0)	0 (0)	0 (0)	0 (0)	0 (0)	0 (0)	0 (0)	0 (0)	0 (0)	0 (0)	0 (0)
Pentaclethra macrophylla	0.8 (1)	0.2 (0.4)	0.2 (0.4)	0 (0)	0 (0)	0 (0)	0 (0)	0 (0)	0 (0)	0 (0)	0 (0)	0 (0)	0 (0)
Pericopsis elata	0.3 (0.5)	0 (0)	0 (0)	0 (0)	0 (0)	0.2 (0.4)	0 (0)	0 (0)	0 (0)	0 (0)	0 (0)	0 (0)	0 (0)
Petersianthus macrocarpus	7.5 (6)	3 (2.6)	1.7 (2.2)	0.7 (0.8)	0.3 (0.5)	0.2 (0.4)	0 (0)	0.2 (0.4)	0.2 (0.4)	0 (0)	0 (0)	0 (0)	0 (0)
Phyllocosmus africanus	0 (0)	0 (0)	0.2 (0.4)	0 (0)	0 (0)	0.2 (0.4)	0 (0)	0 (0)	0 (0)	0 (0)	0 (0)	0.2 (0.4)	0 (0)
Piptadeniastrum africanum	0.2 (0.4)	0.2 (0.4)	0 (0)	0 (0)	0 (0)	0 (0)	0 (0)	0 (0)	0 (0)	0 (0)	0 (0)	0.2 (0.4)	0 (0)
Polyalthia suaveolens	7 (4.7)	9.3 (3.8)	6.7 (2.3)	2.3 (1.8)	1.2 (1.3)	0 (0)	0 (0)	0 (0)	0 (0)	0 (0)	0 (0)	0 (0)	0 (0)
Prioria balsamifera	1.7 (1.8)	0.3 (0.8)	0.7 (0.8)	0.2 (0.4)	0 (0)	0.3 (0.5)	0.3 (0.5)	0 (0)	0.2 (0.4)	0 (0)	0 (0)	0 (0)	0 (0)
Prioria joveri	0.2 (0.4)	0.2 (0.4)	0 (0)	0 (0)	0 (0)	0 (0)	0 (0)	0 (0)	0 (0)	0 (0)	0 (0)	0 (0)	0 (0)
Prioria oxyphylla	2.7 (0.5)	0.7 (0.5)	0.5 (0.5)	0.7 (0.5)	1 (0.6)	0.3 (0.5)	0 (0)	0.5 (0.8)	0.3 (0.5)	0.2 (0.4)	0 (0)	0.2 (0.4)	0 (0)
Pterocarpus soyauxii	0.7 (0.8)	0.2 (0.4)	0.2 (0.4)	0 (0)	0 (0)	0.2 (0.4)	0.2 (0.4)	0 (0)	0.2 (0.4)	0 (0)	0 (0)	0.2 (0.4)	0 (0)
Pterygota bequaertii	0.2 (0.4)	0 (0)	0 (0)	0 (0)	0 (0)	0 (0)	0 (0)	0 (0)	0 (0)	0 (0)	0 (0)	0 (0)	0 (0)
Pycnanthus angolensis	8 (4.9)	1.5 (2)	0 (0)	0 (0)	0 (0)	0 (0)	0 (0)	0 (0)	0 (0)	0 (0)	0 (0)	0 (0)	0 (0)
Quassia undulata	0.2 (0.4)	0 (0)	0 (0)	0 (0)	0 (0)	0 (0)	0 (0)	0 (0)	0 (0)	0 (0)	0 (0)	0 (0)	0 (0)
Ricinodendron heudelotii	0.5 (0.8)	0.3 (0.5)	0 (0)	0 (0)	0 (0)	0 (0)	0 (0)	0 (0)	0 (0)	0 (0)	0 (0)	0 (0)	0 (0)
Rinorea oblongifolia	0.5 (0.8)	0.2 (0.4)	0 (0)	0 (0)	0 (0)	0 (0)	0 (0)	0 (0)	0 (0)	0 (0)	0 (0)	0 (0)	0 (0)
Rinorea sp.	3.2 (2)	0.2 (0.4)	0 (0)	0 (0)	0 (0)	0 (0)	0 (0)	0 (0)	0 (0)	0 (0)	0 (0)	0 (0)	0 (0)
Rothmania libisa	0.3 (0.5)	0 (0)	0 (0)	0 (0)	0 (0)	0 (0)	0 (0)	0 (0)	0 (0)	0 (0)	0 (0)	0 (0)	0 (0)
Rothmannia lujae	0.5 (1.2)	0.2 (0.4)	0 (0)	0 (0)	0 (0)	0 (0)	0 (0)	0 (0)	0 (0)	0 (0)	0 (0)	0 (0)	0 (0)
Rothmannia sp.	0.7 (0.8)	0 (0)	0 (0)	0 (0)	0 (0)	0 (0)	0 (0)	0 (0)	0 (0)	0 (0)	0 (0)	0 (0)	0 (0)
Scorodophloeus zenkeri	7.3 (2.9)	6 (3.7)	7.2 (2.1)	4.7 (2.2)	3.8 (2.5)	5.7 (2.5)	2.3 (1.4)	2 (1.4)	0.5 (0.5)	0.2 (0.4)	0 (0)	0 (0)	0 (0)

Scottellia kamerunensis	0.2 (0.4)	0 (0)	0 (0)	0 (0)	0 (0)	0 (0)	0 (0)	0 (0)	0 (0)	0 (0)	0 (0)	0 (0)	0 (0)
Staudtia kamerunensis	13 (4.6)	0.7 (0.8)	0.2 (0.4)	0 (0)	0.2 (0.4)	0 (0)	0 (0)	0 (0)	0 (0)	0 (0)	0 (0)	0 (0)	0 (0)
Sterculia bequaertii	0 (0)	0.2 (0.4)	0 (0)	0 (0)	0 (0)	0 (0)	0 (0)	0 (0)	0 (0)	0 (0)	0 (0)	0 (0)	0 (0)
Sterculia tragacantha	0.7 (1.2)	0 (0)	0 (0)	0.3 (0.5)	0.5 (0.8)	0 (0)	0 (0)	0 (0)	0 (0)	0 (0)	0 (0)	0 (0)	0 (0)
Strombosia grandifolia	1.2 (1)	0.3 (0.5)	0 (0)	0 (0)	0 (0)	0 (0)	0 (0)	0 (0)	0 (0)	0 (0)	0 (0)	0 (0)	0 (0)
Strombosia nigropunctata	5.2 (3.2)	1.7 (0.8)	0.2 (0.4)	0.3 (0.5)	0 (0)	0 (0)	0 (0)	0 (0)	0 (0)	0 (0)	0 (0)	0 (0)	0 (0)
Strombosia pustulata	1.8 (1.7)	0.7 (0.8)	0.7 (0.5)	0 (0)	0 (0)	0 (0)	0 (0)	0 (0)	0 (0)	0 (0)	0 (0)	0 (0)	0 (0)
Strombosiopsis tetrandra	0.3 (0.5)	0.2 (0.4)	0.5 (0.5)	0 (0)	0 (0)	0 (0)	0 (0)	0 (0)	0 (0)	0 (0)	0 (0)	0 (0)	0 (0)
Symphonia globulifera	0.2 (0.4)	0.2 (0.4)	0 (0)	0 (0)	0.2 (0.4)	0 (0)	0 (0)	0 (0)	0 (0)	0 (0)	0 (0)	0 (0)	0 (0)
Synsepalum subcordatum	0.5 (0.5)	0 (0)	0 (0)	0.2 (0.4)	0 (0)	0 (0)	0 (0)	0 (0)	0 (0)	0 (0)	0 (0)	0 (0)	0.2 (0.4)
Syzygium congolense	0.2 (0.4)	0 (0)	0 (0)	0 (0)	0 (0)	0 (0)	0 (0)	0 (0)	0 (0)	0 (0)	0 (0)	0 (0)	0 (0)
Tessmannia africana	1.2 (1.5)	0 (0)	0 (0)	0 (0)	0.2 (0.4)	0.2 (0.4)	0 (0)	0 (0)	0 (0)	0 (0)	0 (0)	0 (0)	0 (0)
Tessmannia anomala	0.3 (0.5)	0 (0)	0 (0)	0 (0)	0 (0)	0 (0)	0 (0)	0.2 (0.4)	0 (0)	0 (0)	0 (0)	0 (0)	0 (0)
Tetrapleura tetraptera	0 (0)	0.2 (0.4)	0 (0)	0 (0)	0 (0)	0 (0)	0 (0)	0 (0)	0 (0)	0 (0)	0 (0)	0 (0)	0 (0)
Tetrorchidium didymostemon	0.3 (0.5)	0.2 (0.4)	0 (0)	0 (0)	0 (0)	0 (0)	0 (0)	0 (0)	0 (0)	0 (0)	0 (0)	0 (0)	0 (0)
Treculia africana	0 (0)	0 (0)	0 (0)	0 (0)	0.2 (0.4)	0 (0)	0 (0)	0 (0)	0 (0)	0 (0)	0 (0)	0 (0)	0 (0)
Trichilia gilgiana	0.5 (1.2)	0.2 (0.4)	0.2 (0.4)	0 (0)	0 (0)	0 (0)	0 (0)	0 (0)	0 (0)	0 (0)	0 (0)	0 (0)	0 (0)
Trichilia prieuriana	5.7 (4.1)	2.7 (2.3)	0.5 (0.8)	0.3 (0.8)	0.2 (0.4)	0 (0)	0 (0)	0 (0)	0 (0)	0 (0)	0 (0)	0 (0)	0 (0)
Trichilia sp.	1.7 (2)	0.8 (1.6)	0.8 (1)	0 (0)	0 (0)	0 (0)	0 (0)	0 (0)	0 (0)	0 (0)	0 (0)	0 (0)	0 (0)
Trichilia welwitschii	2.3 (2.7)	0 (0)	0 (0)	0 (0)	0 (0)	0 (0)	0 (0)	0 (0)	0 (0)	0 (0)	0 (0)	0 (0)	0 (0)
Trichoscypha oddonii	0.2 (0.4)	0 (0)	0 (0)	0 (0)	0 (0)	0 (0)	0 (0)	0 (0)	0 (0)	0 (0)	0 (0)	0 (0)	0 (0)
Tridesmostemon omphalocarpoides	0.7 (0.8)	0.7 (1)	0.2 (0.4)	0 (0)	0.2 (0.4)	0 (0)	0 (0)	0 (0)	0 (0)	0 (0)	0 (0)	0 (0)	0 (0)
Turraeanthus africanus	5.7 (8.7)	0.7 (1.2)	0.2 (0.4)	0 (0)	0.2 (0.4)	0.2 (0.4)	0 (0)	0 (0)	0 (0)	0 (0)	0 (0)	0 (0)	0 (0)
Unknown	17.8 (7.7)	4.5 (3.1)	1.3 (2.3)	2.2 (1.9)	2.2 (1.7)	1.7 (1.2)	1.3 (1.2)	0.7 (0.8)	0 (0)	0 (0)	0 (0)	0 (0)	0 (0)
Vitex welwitschii	0.5 (0.8)	0.2 (0.4)	0 (0)	0.2 (0.4)	0 (0)	0 (0)	0 (0)	0 (0)	0 (0)	0 (0)	0 (0)	0 (0)	0 (0)
Xylia ghesquierei	0 (0)	0 (0)	0 (0)	0.2 (0.4)	0.2 (0.4)	0 (0)	0 (0)	0 (0)	0 (0)	0 (0)	0 (0)	0 (0)	0 (0)
Xylopia hypolampra	0.2 (0.4)	0.2 (0.4)	0 (0)	0.2 (0.4)	0 (0)	0 (0)	0 (0)	0 (0)	0 (0)	0 (0)	0 (0)	0 (0)	0 (0)
Zanthoxylum inaequalis	0 (0)	0 (0)	0 (0)	0.2 (0.4)	0 (0)	0 (0)	0 (0)	0 (0)	0 (0)	0 (0)	0 (0)	0 (0)	0 (0)
