# Supplementary material for: Aboveground vs. Belowground Carbon Stocks in African Tropical Lowland Rainforest: Drivers and Implications
Source: PLoS One. 2015 Nov 24;10(11):e0143209. doi: 10.1371/journal.pone.0143209 (PMC4657968; doi:10.1371/journal.pone.0143209)
Supplement: S3 Table — (PDF) [file pone.0143209.s006.pdf]

S3 Table. Average number of stems with standard deviation in brackets of tree species per hectare in Yangambi within different diameter classes.

Species	10-20	20-30	30-40	40-50	50-60	60-70	70-80	80-90	90-100	100-110	110-120	120-130	130-140	140-150	150-160
Afrostyrax lepidophyllus	0.6 (1.3)	0 (0)	0 (0)	0.2 (0.4)	0 (0)	0 (0)	0 (0)	0 (0)	0 (0)	0 (0)	0 (0)	0 (0)	0 (0)	0 (0)	0 (0)
Afzelia bipindensis	0 (0)	0.2 (0.4)	0 (0)	0 (0)	0 (0)	0 (0)	0 (0)	0 (0)	0 (0)	0 (0)	0 (0)	0 (0)	0 (0)	0 (0)	0 (0)
Aidia micrantha	0.4 (0.9)	0 (0)	0 (0)	0 (0)	0 (0)	0 (0)	0 (0)	0 (0)	0 (0)	0 (0)	0 (0)	0 (0)	0 (0)	0 (0)	0 (0)
Albizia sp.	0.4 (0.9)	0 (0)	0 (0)	0.2 (0.4)	0.2 (0.4)	0 (0)	0 (0)	0 (0)	0 (0)	0 (0)	0 (0)	0 (0)	0 (0)	0 (0)	0 (0)
Allanblackia floribunda	0.4 (0.9)	0 (0)	0 (0)	0 (0)	0 (0)	0 (0)	0 (0)	0 (0)	0 (0)	0 (0)	0 (0)	0 (0)	0 (0)	0 (0)	0 (0)
Alstonia boonei	0 (0)	0 (0)	0.2 (0.4)	0 (0)	0.2 (0.4)	0 (0)	0 (0)	0 (0)	0 (0)	0.2 (0.4)	0 (0)	0 (0)	0 (0)	0 (0)	0 (0)
Anonidium mannii	2.6 (2.7)	4 (3.9)	4.8 (5.2)	3.2 (2.6)	0.6 (1.3)	0 (0)	0 (0)	0 (0)	0 (0)	0.2 (0.4)	0 (0)	0 (0)	0 (0)	0 (0)	0 (0)
Anthonotha macrophylla	1 (1.2)	0 (0)	0 (0)	0 (0)	0 (0)	0 (0)	0 (0)	0 (0)	0 (0)	0 (0)	0 (0)	0 (0)	0 (0)	0 (0)	0 (0)
Antrocaryon nannanii	0 (0)	0 (0)	0 (0)	0.2 (0.4)	0 (0)	0 (0)	0 (0)	0 (0)	0 (0)	0 (0)	0 (0)	0 (0)	0 (0)	0 (0)	0 (0)
Autranella congolensis	0 (0)	0 (0)	0 (0)	0 (0)	0 (0)	0 (0)	0 (0)	0 (0)	0 (0)	0 (0)	0 (0)	0 (0)	0 (0)	0 (0)	0.2 (0.4)
Baphia capparidifolia	0.2 (0.4)	0 (0)	0 (0)	0 (0)	0 (0)	0 (0)	0 (0)	0 (0)	0 (0)	0 (0)	0 (0)	0 (0)	0 (0)	0 (0)	0 (0)
Barteria fistulosa	0.4 (0.9)	0 (0)	0 (0)	0 (0)	0 (0)	0 (0)	0 (0)	0 (0)	0 (0)	0 (0)	0 (0)	0 (0)	0 (0)	0 (0)	0 (0)
Barteria nigritiana	0.4 (0.5)	0 (0)	0 (0)	0 (0)	0 (0)	0 (0)	0 (0)	0 (0)	0 (0)	0 (0)	0 (0)	0 (0)	0 (0)	0 (0)	0 (0)
Beilschmiedia gilbertii	0.2 (0.4)	0 (0)	0 (0)	0 (0)	0 (0)	0 (0)	0 (0)	0 (0)	0 (0)	0 (0)	0 (0)	0 (0)	0 (0)	0 (0)	0 (0)
Blighia welwitschii	0.6 (0.5)	0.4 (0.5)	0.2 (0.4)	0 (0)	0.2 (0.4)	0 (0)	0 (0)	0 (0)	0 (0)	0 (0)	0 (0)	0 (0)	0 (0)	0 (0)	0 (0)
Canarium schweinfurthii	0.6 (1.3)	0 (0)	0 (0)	0 (0)	0 (0)	0 (0)	0 (0)	0 (0)	0 (0)	0 (0)	0 (0)	0 (0)	0 (0)	0 (0)	0 (0)
Carapa procera	12 (1.7)	0.4 (0.5)	0 (0)	0 (0)	0 (0)	0 (0)	0 (0)	0 (0)	0 (0)	0 (0)	0 (0)	0 (0)	0 (0)	0 (0)	0 (0)
Celtis mildbraedii	0.6 (0.9)	1.4 (1.3)	0.2 (0.4)	0 (0)	0 (0)	0.2 (0.4)	0.2 (0.4)	0.4 (0.5)	0 (0)	0 (0)	0 (0)	0 (0)	0 (0)	0 (0)	0 (0)
Celtis sp.	0.2 (0.4)	0 (0)	0 (0)	0 (0)	0 (0)	0 (0)	0 (0)	0 (0)	0 (0)	0 (0)	0 (0)	0 (0)	0 (0)	0 (0)	0 (0)
Celtis tessmannii	1.4 (0.5)	1.4 (0.9)	0.2 (0.4)	0.6 (0.5)	0 (0)	0 (0)	0 (0)	0 (0)	0 (0)	0 (0)	0 (0)	0 (0)	0 (0)	0 (0)	0 (0)
Chrysophyllum africanum	1.8 (0.8)	0.8 (1.3)	0.4 (0.5)	0.2 (0.4)	0.2 (0.4)	0 (0)	0 (0)	0 (0)	0 (0)	0 (0)	0 (0)	0 (0)	0 (0)	0 (0)	0 (0)
Chrysophyllum beguei	0.4 (0.9)	0.4 (0.5)	0 (0)	0 (0)	0 (0)	0 (0)	0 (0)	0 (0)	0 (0)	0 (0)	0 (0)	0 (0)	0 (0)	0 (0)	0 (0)
Chrysophyllum lacourtianum	1 (1.4)	0.6 (0.9)	0.6 (0.9)	0.8 (0.4)	1 (1.2)	0.6 (0.9)	0.4 (0.9)	0 (0)	0 (0)	0 (0)	0 (0)	0 (0)	0 (0)	0 (0)	0 (0)
Chrysophyllum pruniforme	0 (0)	0 (0)	0.2 (0.4)	0 (0)	0.2 (0.4)	0 (0)	0 (0)	0 (0)	0 (0)	0 (0)	0 (0)	0 (0)	0 (0)	0 (0)	0 (0)
Chrysophyllum pruniformis	0.2 (0.4)	0 (0)	0 (0)	0 (0)	0 (0)	0 (0)	0 (0)	0 (0)	0 (0)	0 (0)	0 (0)	0 (0)	0 (0)	0 (0)	0 (0)
Chrysophyllum sp.	0.2 (0.4)	0 (0)	0.2 (0.4)	0 (0)	0 (0)	0.4 (0.9)	0 (0)	0 (0)	0 (0)	0 (0)	0 (0)	0 (0)	0 (0)	0 (0)	0 (0)
Cleistanthus pynaertii	0.2 (0.4)	0 (0)	0 (0)	0 (0)	0 (0)	0 (0)	0 (0)	0 (0)	0 (0)	0 (0)	0 (0)	0 (0)	0 (0)	0 (0)	0 (0)

Coelocaryon preussii	7.4 (7.2)	0.8 (1.8)	0.2 (0.4)	0 (0)	0 (0)	0 (0)	0 (0)	0 (0)	0 (0)	0 (0)	0 (0)	0 (0)	0 (0)	0 (0)	0 (0)
Cola acuminata	0 (0)	0.2 (0.4)	0 (0)	0 (0)	0 (0)	0 (0)	0 (0)	0 (0)	0 (0)	0 (0)	0 (0)	0 (0)	0 (0)	0 (0)	0 (0)
Cola griseiflora	7.8 (6.9)	1.4 (1.9)	0.2 (0.4)	0 (0)	0 (0)	0 (0)	0 (0)	0 (0)	0 (0)	0 (0)	0 (0)	0 (0)	0 (0)	0 (0)	0 (0)
Cola lateritia	0.4 (0.5)	0.4 (0.5)	0.4 (0.9)	0 (0)	0.2 (0.4)	0.2 (0.4)	0 (0)	0 (0)	0 (0)	0 (0)	0 (0)	0 (0)	0 (0)	0 (0)	0 (0)
Cola sp.	0 (0)	0.2 (0.4)	0 (0)	0 (0)	0 (0)	0 (0)	0 (0)	0 (0)	0 (0)	0 (0)	0 (0)	0 (0)	0 (0)	0 (0)	0 (0)
Combretum lokele	0.6 (0.9)	0 (0)	0.6 (0.9)	0.2 (0.4)	0.2 (0.4)	0 (0)	0 (0)	0.2 (0.4)	0 (0)	0 (0)	0 (0)	0.2 (0.4)	0 (0)	0 (0)	0 (0)
Cynometra hankei	0.2 (0.4)	0 (0)	0 (0)	0 (0)	0.4 (0.5)	0 (0)	0 (0)	0.2 (0.4)	0.2 (0.4)	0 (0)	0 (0)	0.2 (0.4)	0 (0)	0 (0)	0 (0)
Dacryodes edulis	0.4 (0.5)	0.2 (0.4)	0 (0)	0 (0)	0 (0)	0 (0)	0 (0)	0 (0)	0 (0)	0 (0)	0 (0)	0 (0)	0 (0)	0 (0)	0 (0)
Dacryodes osika	0.2 (0.4)	0 (0)	0 (0)	0 (0)	0 (0)	0 (0)	0 (0)	0 (0)	0 (0)	0 (0)	0 (0)	0 (0)	0 (0)	0 (0)	0 (0)
Desplatsia dewevrei	0.2 (0.4)	0 (0)	0.2 (0.4)	0 (0)	0 (0)	0 (0)	0 (0)	0 (0)	0 (0)	0 (0)	0 (0)	0 (0)	0 (0)	0 (0)	0 (0)
Dialium corbisieri	0.4 (0.5)	0 (0)	0 (0)	0 (0)	0 (0)	0 (0)	0 (0)	0 (0)	0 (0)	0 (0)	0 (0)	0 (0)	0 (0)	0 (0)	0 (0)
Dialium excelsum	0 (0)	0.2 (0.4)	0 (0)	0.4 (0.9)	0 (0)	0 (0)	0 (0)	0 (0)	0 (0)	0 (0)	0 (0)	0 (0)	0 (0)	0 (0)	0 (0)
Dialium pachyphyllum	3 (1.2)	0.4 (0.5)	0 (0)	0.2 (0.4)	0 (0)	0.4 (0.5)	0 (0)	0 (0)	0 (0)	0 (0)	0 (0)	0 (0)	0 (0)	0 (0)	0 (0)
Dialium sp.	0.8 (1.3)	0.4 (0.9)	0 (0)	0.2 (0.4)	0 (0)	0.2 (0.4)	0 (0)	0 (0)	0 (0)	0 (0)	0 (0)	0 (0)	0 (0)	0 (0)	0 (0)
Diospyros boala	1.8 (1.5)	0.4 (0.9)	0 (0)	0 (0)	0 (0)	0 (0)	0 (0)	0 (0)	0 (0)	0 (0)	0 (0)	0 (0)	0 (0)	0 (0)	0 (0)
Diospyros crassiflora	1 (0.7)	0.8 (0.8)	0.6 (0.5)	0 (0)	0.2 (0.4)	0 (0)	0 (0)	0 (0)	0 (0)	0 (0)	0 (0)	0 (0)	0 (0)	0 (0)	0 (0)
Diospyros sp.	3 (2.4)	0 (0)	0.2 (0.4)	0 (0)	0 (0)	0 (0)	0 (0)	0 (0)	0 (0)	0 (0)	0 (0)	0 (0)	0 (0)	0 (0)	0 (0)
Drypetes angustifolia	0.2 (0.4)	0 (0)	0 (0)	0 (0)	0 (0)	0 (0)	0 (0)	0 (0)	0 (0)	0 (0)	0 (0)	0 (0)	0 (0)	0 (0)	0 (0)
Drypetes anthiatif	0.2 (0.4)	0 (0)	0 (0)	0 (0)	0 (0)	0 (0)	0 (0)	0 (0)	0 (0)	0 (0)	0 (0)	0 (0)	0 (0)	0 (0)	0 (0)
Drypetes gossweileri	3.6 (3)	2 (2)	1 (1.2)	0.6 (0.9)	0.4 (0.5)	0 (0)	0 (0)	0 (0)	0 (0)	0 (0)	0 (0)	0 (0)	0 (0)	0 (0)	0 (0)
Drypetes leonensis	1 (1.7)	0 (0)	0 (0)	0 (0)	0 (0)	0 (0)	0 (0)	0 (0)	0 (0)	0 (0)	0 (0)	0 (0)	0 (0)	0 (0)	0 (0)
Drypetes likwa	0.2 (0.4)	0 (0)	0 (0)	0 (0)	0 (0)	0 (0)	0 (0)	0 (0)	0 (0)	0 (0)	0 (0)	0 (0)	0 (0)	0 (0)	0 (0)
Drypetes sp.	4.2 (2.9)	1.8 (1.6)	0.2 (0.4)	0.4 (0.5)	0 (0)	0 (0)	0 (0)	0 (0)	0 (0)	0 (0)	0 (0)	0 (0)	0 (0)	0 (0)	0 (0)
Entandrophragma angolense	0.2 (0.4)	0 (0)	0 (0)	0 (0)	0 (0)	0 (0)	0 (0)	0 (0)	0 (0)	0 (0)	0 (0)	0 (0)	0 (0)	0 (0)	0 (0)
Entandrophragma cylindricum	0.4 (0.5)	0 (0)	0.2 (0.4)	0 (0)	0 (0)	0 (0)	0.2 (0.4)	0 (0)	0 (0)	0 (0)	0 (0)	0 (0)	0 (0)	0 (0)	0 (0)
Entandrophragma sp.	0 (0)	0 (0)	0 (0)	0.2 (0.4)	0 (0)	0 (0)	0 (0)	0 (0)	0 (0)	0 (0)	0 (0)	0 (0)	0 (0)	0 (0)	0 (0)
Entandrophragma utile	0.2 (0.4)	0.2 (0.4)	0 (0)	0 (0)	0 (0)	0 (0)	0 (0)	0.2 (0.4)	0 (0)	0.2 (0.4)	0 (0)	0 (0)	0 (0)	0 (0)	0 (0)
Erythrophleum suaveolens	0 (0)	0.4 (0.5)	0.2 (0.4)	0 (0)	0 (0)	0.4 (0.5)	0 (0)	0.4 (0.5)	0 (0)	0 (0)	0.2 (0.4)	0 (0)	0 (0)	0 (0)	0 (0)
Funtumia africana	0.2 (0.4)	0 (0)	0 (0)	0 (0)	0 (0)	0 (0)	0 (0)	0 (0)	0 (0)	0 (0)	0 (0)	0 (0)	0 (0)	0 (0)	0 (0)
Garcinia punctata	5 (1.6)	2 (2.3)	0.6 (0.9)	0 (0)	0 (0)	0 (0)	0 (0)	0 (0)	0 (0)	0 (0)	0 (0)	0 (0)	0 (0)	0 (0)	0 (0)

Garcinia smeathmannii	1.2 (1.8)	0 (0)	0 (0)	0 (0)	0 (0)	0 (0)	0 (0)	0 (0)	0 (0)	0 (0)	0 (0)	0 (0)	0 (0)	0 (0)	0 (0)
Garcinia sp.	0 (0)	0.2 (0.4)	0 (0)	0 (0)	0 (0)	0 (0)	0 (0)	0 (0)	0 (0)	0 (0)	0 (0)	0 (0)	0 (0)	0 (0)	0 (0)
Glyphaea brevis	0.4 (0.9)	0 (0)	0 (0)	0 (0)	0 (0)	0 (0)	0 (0)	0 (0)	0 (0)	0 (0)	0 (0)	0 (0)	0 (0)	0 (0)	0 (0)
Greenwayodendron suaveolens	3.4 (3)	1.8 (1.6)	1.8 (2)	0.2 (0.4)	0 (0)	0 (0)	0 (0)	0 (0)	0 (0)	0 (0)	0 (0)	0 (0)	0 (0)	0 (0)	0 (0)
Grewia oligoneura	0.2 (0.4)	0 (0)	0 (0)	0 (0)	0 (0)	0 (0)	0 (0)	0 (0)	0 (0)	0 (0)	0 (0)	0 (0)	0 (0)	0 (0)	0 (0)
Grewia sp.	0.4 (0.5)	0 (0)	0 (0)	0 (0)	0 (0)	0 (0)	0 (0)	0 (0)	0 (0)	0 (0)	0 (0)	0 (0)	0 (0)	0 (0)	0 (0)
Grossera multinervis	2 (3.9)	1.2 (2.7)	0 (0)	0 (0)	0 (0)	0 (0)	0 (0)	0 (0)	0 (0)	0 (0)	0 (0)	0 (0)	0 (0)	0 (0)	0 (0)
Guarea cedrata	0.2 (0.4)	0 (0)	0 (0)	0 (0)	0 (0)	0.4 (0.9)	0.2 (0.4)	0 (0)	0 (0)	0 (0)	0 (0)	0 (0)	0 (0)	0 (0)	0 (0)
Guarea thompsonii	4 (4.4)	3 (1.2)	2 (1.4)	0.6 (0.9)	0 (0)	0 (0)	0 (0)	0 (0)	0 (0)	0 (0)	0 (0)	0 (0)	0 (0)	0 (0)	0 (0)
Hallea stipulosa	0.2 (0.4)	0 (0)	0 (0)	0 (0)	0 (0)	0 (0)	0 (0)	0 (0)	0 (0)	0 (0)	0 (0)	0 (0)	0 (0)	0 (0)	0 (0)
Hannoa klaineana	0 (0)	0.2 (0.4)	0 (0)	0 (0)	0 (0)	0 (0)	0 (0)	0 (0)	0 (0)	0 (0)	0 (0)	0 (0)	0 (0)	0 (0)	0 (0)
Heisteria parvifolia	0.2 (0.4)	0 (0)	0 (0)	0 (0)	0 (0)	0 (0)	0 (0)	0 (0)	0 (0)	0 (0)	0 (0)	0 (0)	0 (0)	0 (0)	0 (0)
Homalium africanum	0.4 (0.9)	0 (0)	0 (0)	0 (0)	0 (0)	0 (0)	0 (0)	0 (0)	0 (0)	0 (0)	0 (0)	0 (0)	0 (0)	0 (0)	0 (0)
Homalium longistylum	0 (0)	0 (0)	0.2 (0.4)	0 (0)	0 (0)	0 (0)	0 (0)	0 (0)	0 (0)	0 (0)	0 (0)	0 (0)	0 (0)	0 (0)	0 (0)
Hua gabonii	0.2 (0.4)	0 (0)	0 (0)	0 (0)	0 (0)	0 (0)	0 (0)	0 (0)	0 (0)	0 (0)	0 (0)	0 (0)	0 (0)	0 (0)	0 (0)
Inconnu	9 (5.6)	3.2 (2.2)	2 (2.3)	1.4 (1.3)	0.8 (0.8)	0.2 (0.4)	0 (0)	0 (0)	0.2 (0.4)	0 (0)	0 (0)	0 (0)	0 (0)	0 (0)	0 (0)
Irvingia gabonensis	0 (0)	0 (0)	0 (0)	0 (0)	0 (0)	0 (0)	0.2 (0.4)	0.2 (0.4)	0.2 (0.4)	0 (0)	0.2 (0.4)	0 (0)	0 (0)	0 (0)	0 (0)
Irvingia grandifolia	0.2 (0.4)	0.2 (0.4)	0.2 (0.4)	0 (0)	0.2 (0.4)	0.2 (0.4)	0 (0)	0 (0)	0 (0)	0 (0)	0.2 (0.4)	0 (0)	0 (0)	0 (0)	0 (0)
Isolona thonneri	0.2 (0.4)	0 (0)	0 (0)	0 (0)	0 (0)	0 (0)	0 (0)	0 (0)	0 (0)	0 (0)	0 (0)	0 (0)	0 (0)	0 (0)	0 (0)
Klainedoxa gabonensis	0.4 (0.5)	0.2 (0.4)	0.2 (0.4)	0.2 (0.4)	0 (0)	0 (0)	0.4 (0.5)	0.2 (0.4)	0 (0)	0 (0)	0 (0)	0 (0)	0 (0)	0 (0)	0 (0)
Lovoa trichilioides	0.6 (0.9)	0 (0)	0 (0)	0 (0)	0.2 (0.4)	0 (0)	0 (0)	0 (0)	0 (0)	0 (0)	0 (0)	0 (0)	0 (0)	0 (0)	0 (0)
Macaranga monandra	0.2 (0.4)	0.2 (0.4)	0 (0)	0 (0)	0 (0)	0 (0)	0 (0)	0 (0)	0 (0)	0 (0)	0 (0)	0 (0)	0 (0)	0 (0)	0 (0)
Massularia acuminata	0.8 (0.8)	0 (0)	0 (0)	0 (0)	0 (0)	0 (0)	0 (0)	0 (0)	0 (0)	0 (0)	0 (0)	0 (0)	0 (0)	0 (0)	0 (0)
Microdesmis sp.	4.2 (9.4)	3.8 (8.5)	0 (0)	0 (0)	0.2 (0.4)	0 (0)	0 (0)	0 (0)	0 (0)	0 (0)	0 (0)	0 (0)	0 (0)	0 (0)	0 (0)
Microdesmis yafungana	11.4 (25.5)	3.2 (7.2)	0.4 (0.9)	0 (0)	0 (0)	0 (0)	0 (0)	0 (0)	0 (0)	0 (0)	0 (0)	0 (0)	0 (0)	0 (0)	0 (0)
Milicia excelsa	0 (0)	0.2 (0.4)	0 (0)	0 (0)	0 (0)	0 (0)	0 (0)	0 (0)	0 (0)	0 (0)	0 (0)	0 (0)	0 (0)	0 (0)	0 (0)
Millettia drastica	0.4 (0.5)	0.2 (0.4)	0.2 (0.4)	0 (0)	0 (0)	0 (0)	0 (0)	0 (0)	0 (0)	0 (0)	0 (0)	0 (0)	0 (0)	0 (0)	0 (0)
Millettia dubia	0.2 (0.4)	0 (0)	0 (0)	0 (0)	0 (0)	0 (0)	0 (0)	0 (0)	0 (0)	0 (0)	0 (0)	0 (0)	0 (0)	0 (0)	0 (0)
Millettia hylobia	0.2 (0.4)	0 (0)	0 (0)	0 (0)	0 (0)	0 (0)	0 (0)	0 (0)	0 (0)	0 (0)	0 (0)	0 (0)	0 (0)	0 (0)	0 (0)
Monodora angolensis	0.4 (0.9)	0.2 (0.4)	0.2 (0.4)	0 (0)	0 (0)	0 (0)	0 (0)	0 (0)	0 (0)	0 (0)	0 (0)	0 (0)	0 (0)	0 (0)	0 (0)

Monodora myristica	0.6 (0.9)	0 (0)	0 (0)	0 (0)	0 (0)	0 (0)	0 (0)	0 (0)	0 (0)	0 (0)	0 (0)	0 (0)	0 (0)	0 (0)	0 (0)
Musanga cecropioides	0.4 (0.5)	0.4 (0.5)	1.6 (1.5)	0.4 (0.5)	0 (0)	0 (0)	0 (0)	0 (0)	0 (0)	0 (0)	0 (0)	0 (0)	0 (0)	0 (0)	0 (0)
Myrianthus arboreus	0.6 (0.9)	0 (0)	0 (0)	0 (0)	0 (0)	0 (0)	0 (0)	0 (0)	0 (0)	0 (0)	0 (0)	0 (0)	0 (0)	0 (0)	0 (0)
Omphalocarpum lecomteanum	0 (0)	0 (0)	0 (0)	0 (0)	0 (0)	0 (0)	0 (0)	0 (0)	0 (0)	0.2 (0.4)	0 (0)	0 (0)	0 (0)	0 (0)	0 (0)
Oncoba glauca	0.2 (0.4)	0 (0)	0 (0)	0 (0)	0 (0)	0 (0)	0 (0)	0 (0)	0 (0)	0 (0)	0 (0)	0 (0)	0 (0)	0 (0)	0 (0)
Ongokea gore	0 (0)	0 (0)	0.2 (0.4)	0.2 (0.4)	0.4 (0.9)	0.2 (0.4)	0 (0)	0.2 (0.4)	0 (0)	0 (0)	0 (0)	0 (0)	0 (0)	0 (0)	0 (0)
Oxyanthus speciosusÂ	0.2 (0.4)	0 (0)	0 (0)	0 (0)	0 (0)	0 (0)	0 (0)	0 (0)	0 (0)	0 (0)	0 (0)	0 (0)	0 (0)	0 (0)	0 (0)
Pancovia harmsiana	10 (11.3)	3 (5.6)	0 (0)	0 (0)	0 (0)	0 (0)	0 (0)	0 (0)	0 (0)	0 (0)	0 (0)	0 (0)	0 (0)	0 (0)	0 (0)
Pancovia laurentii	2.8 (2.8)	1.6 (1.3)	0.4 (0.5)	0 (0)	0 (0)	0 (0)	0 (0)	0 (0)	0 (0)	0 (0)	0 (0)	0 (0)	0 (0)	0 (0)	0 (0)
Pancovia sp.	0.2 (0.4)	0 (0)	0 (0)	0 (0)	0 (0)	0 (0)	0 (0)	0 (0)	0 (0)	0 (0)	0 (0)	0 (0)	0 (0)	0 (0)	0 (0)
Panda oleosa	3.4 (2.5)	4.6 (2.5)	2.8 (0.8)	3.2 (2.7)	2 (2.8)	0.6 (0.9)	0 (0)	0.2 (0.4)	0 (0)	0 (0)	0 (0)	0 (0)	0 (0)	0 (0)	0 (0)
Parinari excelsa	0.2 (0.4)	0 (0)	0 (0)	0 (0)	0 (0)	0 (0)	0 (0)	0 (0)	0 (0)	0 (0)	0 (0)	0 (0)	0 (0)	0 (0)	0 (0)
Pentaclethra macrophylla	0.4 (0.5)	0.4 (0.9)	0.2 (0.4)	0.2 (0.4)	0 (0)	0 (0)	0 (0)	0 (0)	0 (0)	0 (0)	0 (0)	0 (0)	0 (0)	0 (0)	0 (0)
Pericopsis elata	0 (0)	0 (0)	0 (0)	0 (0)	0 (0)	0 (0)	0 (0)	0 (0)	0.2 (0.4)	0 (0)	0 (0)	0.2 (0.4)	0 (0)	0 (0)	0 (0)
Petersianthus macrocarpus	11.4 (9.6)	4.8 (4.2)	3.6 (2.7)	1.8 (1.9)	1.4 (1.1)	0.6 (0.9)	0.2 (0.4)	0.2 (0.4)	0.2 (0.4)	0.2 (0.4)	0 (0)	0 (0)	0 (0)	0 (0)	0 (0)
Piptadeniastrum africanum	0.2 (0.4)	0.2 (0.4)	0 (0)	0.2 (0.4)	0 (0)	0.2 (0.4)	0 (0)	0 (0)	0 (0)	0 (0)	0 (0)	0 (0)	0 (0)	0 (0)	0 (0)
Pleiocarpa pycnantha	1.2 (1.8)	0 (0)	0 (0)	0 (0)	0 (0)	0 (0)	0 (0)	0 (0)	0 (0)	0 (0)	0 (0)	0 (0)	0 (0)	0 (0)	0 (0)
Polyalthia suaveolens	2.2 (3)	1.8 (2.5)	0.6 (0.9)	0.6 (0.9)	0 (0)	0 (0)	0 (0)	0 (0)	0 (0)	0 (0)	0 (0)	0 (0)	0 (0)	0 (0)	0 (0)
Prioria balsamifera	3.2 (3.5)	0.4 (0.9)	0.2 (0.4)	0 (0)	0 (0)	0 (0)	0 (0)	0 (0)	0 (0)	0 (0)	0 (0)	0 (0)	0 (0)	0 (0)	0 (0)
Prioria oxyphylla	0 (0)	0.6 (0.9)	0.2 (0.4)	0 (0)	0 (0)	0 (0)	0 (0)	0 (0)	0 (0)	0 (0)	0 (0)	0 (0)	0 (0)	0 (0)	0 (0)
Prioria sp.	0 (0)	0 (0)	0 (0)	0 (0)	0 (0)	0 (0)	0 (0)	0.2 (0.4)	0 (0)	0 (0)	0 (0)	0 (0)	0 (0)	0 (0)	0 (0)
Pterocarpus soyauxii	1 (1)	0.2 (0.4)	0 (0)	0 (0)	0 (0)	0.2 (0.4)	0 (0)	0 (0)	0 (0)	0 (0)	0 (0)	0 (0)	0 (0)	0 (0)	0 (0)
Pterygota bequaertii	0 (0)	0 (0)	0 (0)	0.2 (0.4)	0 (0)	0 (0)	0 (0)	0 (0)	0 (0)	0 (0)	0 (0)	0 (0)	0 (0)	0 (0)	0 (0)
Pycnanthus angolensis	2.6 (1.5)	0.4 (0.5)	0 (0)	0 (0)	0 (0)	0 (0)	0 (0)	0 (0)	0 (0)	0 (0)	0 (0)	0 (0)	0 (0)	0 (0)	0 (0)
Pycnanthus marshalianus	0 (0)	0 (0)	0 (0)	0 (0)	0 (0)	0 (0)	0.2 (0.4)	0 (0)	0 (0)	0 (0)	0 (0)	0 (0)	0 (0)	0 (0)	0 (0)
Quassia undulata	0.6 (0.9)	0.6 (0.9)	0 (0)	0.2 (0.4)	0 (0)	0 (0)	0 (0)	0 (0)	0 (0)	0 (0)	0 (0)	0 (0)	0 (0)	0 (0)	0 (0)
Randia africana	0.2 (0.4)	0 (0)	0 (0)	0 (0)	0 (0)	0 (0)	0 (0)	0 (0)	0 (0)	0 (0)	0 (0)	0 (0)	0 (0)	0 (0)	0 (0)
Ricinodendron heudelotii	0 (0)	0 (0)	0 (0)	0.2 (0.4)	0 (0)	0 (0)	0.2 (0.4)	0 (0)	0 (0)	0 (0)	0 (0)	0 (0)	0 (0)	0 (0)	0 (0)
Rinorea oblongifolia	1.4 (1.5)	0.8 (0.8)	0 (0)	0 (0)	0 (0)	0 (0)	0 (0)	0 (0)	0 (0)	0 (0)	0 (0)	0 (0)	0 (0)	0 (0)	0 (0)
Rinorea sp.	0.8 (1.3)	0.2 (0.4)	0 (0)	0 (0)	0 (0)	0 (0)	0 (0)	0 (0)	0 (0)	0 (0)	0 (0)	0 (0)	0 (0)	0 (0)	0 (0)

Rothmannia whitfieldii	0.2 (0.4)	0 (0)	0 (0)	0 (0)	0 (0)	0 (0)	0 (0)	0 (0)	0 (0)	0 (0)	0 (0)	0 (0)	0 (0)	0 (0)	0 (0)
Scorodophloeus zenkeri	7 (5.8)	6.2 (4.8)	9.4 (3.2)	9.2 (6.8)	5 (3.2)	2.2 (2)	0.8 (0.8)	0.4 (0.5)	0 (0)	0 (0)	0 (0)	0 (0)	0 (0)	0 (0)	0 (0)
Spathodea campanulata	0.2 (0.4)	0 (0)	0 (0)	0 (0)	0 (0)	0 (0)	0 (0)	0 (0)	0 (0)	0 (0)	0 (0)	0 (0)	0 (0)	0 (0)	0 (0)
Staudtia kamerunensis	18.8 (8.7)	4.8 (3)	1.6 (1.1)	0.4 (0.5)	0.2 (0.4)	0.2 (0.4)	0 (0)	0.2 (0.4)	0 (0)	0 (0)	0 (0)	0 (0)	0 (0)	0 (0)	0 (0)
Sterculia bequaertii	0 (0)	0 (0)	0.2 (0.4)	0 (0)	0 (0)	0 (0)	0 (0)	0 (0)	0 (0)	0 (0)	0 (0)	0 (0)	0 (0)	0 (0)	0 (0)
Sterculia sp.	0 (0)	0 (0)	0 (0)	0.2 (0.4)	0 (0)	0 (0)	0 (0)	0 (0)	0 (0)	0 (0)	0 (0)	0 (0)	0 (0)	0 (0)	0 (0)
Sterculia trachadantha	0 (0)	0 (0)	0 (0)	0 (0)	0.2 (0.4)	0 (0)	0.2 (0.4)	0 (0)	0 (0)	0 (0)	0 (0)	0 (0)	0 (0)	0 (0)	0 (0)
Strombosia grandifolia	4.6 (2.2)	3.6 (1.8)	1.6 (0.9)	0.2 (0.4)	0.2 (0.4)	0 (0)	0.2 (0.4)	0 (0)	0 (0)	0 (0)	0 (0)	0 (0)	0 (0)	0 (0)	0 (0)
Strombosia pustulata	1.6 (2.5)	0 (0)	0.6 (1.3)	0.2 (0.4)	0.6 (0.9)	0 (0)	0 (0)	0 (0)	0 (0)	0 (0)	0 (0)	0 (0)	0 (0)	0 (0)	0 (0)
Strombosia sp.	0.2 (0.4)	0 (0)	0 (0)	0 (0)	0 (0)	0 (0)	0 (0)	0 (0)	0 (0)	0 (0)	0 (0)	0 (0)	0 (0)	0 (0)	0 (0)
Strombosiopsis tetrandra	0 (0)	0 (0)	0.4 (0.9)	0.4 (0.5)	1 (1.7)	0.4 (0.9)	0.2 (0.4)	0.2 (0.4)	0 (0)	0 (0)	0 (0)	0 (0)	0 (0)	0 (0)	0 (0)
Symphonia globulifera	0 (0)	0 (0)	0.2 (0.4)	0 (0)	0 (0)	0.2 (0.4)	0 (0)	0 (0)	0 (0)	0 (0)	0 (0)	0 (0)	0 (0)	0 (0)	0 (0)
Synsepalum longikineyi	0.2 (0.4)	0 (0)	0 (0)	0 (0)	0 (0)	0 (0)	0 (0)	0 (0)	0 (0)	0 (0)	0 (0)	0 (0)	0 (0)	0 (0)	0 (0)
Synsepalum subcordatum	0.6 (0.9)	1.6 (0.9)	0.4 (0.5)	0.4 (0.5)	0 (0)	0 (0)	0 (0)	0 (0)	0 (0)	0 (0)	0 (0)	0 (0)	0 (0)	0 (0)	0 (0)
Tabernaemontana crassa	1.4 (0.5)	0 (0)	0 (0)	0 (0)	0 (0)	0 (0)	0 (0)	0 (0)	0 (0)	0 (0)	0 (0)	0 (0)	0 (0)	0 (0)	0 (0)
Tessmannia africana	0 (0)	0 (0)	0.2 (0.4)	0 (0)	0 (0)	0 (0)	0.2 (0.4)	0 (0)	0 (0)	0 (0)	0 (0)	0 (0)	0 (0)	0 (0)	0 (0)
Tetrapleura tetraptera	0.2 (0.4)	0 (0)	0 (0)	0.2 (0.4)	0 (0)	0 (0)	0 (0)	0 (0)	0 (0)	0 (0)	0 (0)	0 (0)	0 (0)	0 (0)	0 (0)
Tetrorchidium didymostemon	0.4 (0.9)	0 (0)	0 (0)	0 (0)	0 (0)	0 (0)	0 (0)	0 (0)	0 (0)	0 (0)	0 (0)	0 (0)	0 (0)	0 (0)	0 (0)
Thomandersia sp.	0.2 (0.4)	0 (0)	0 (0)	0 (0)	0 (0)	0 (0)	0 (0)	0 (0)	0 (0)	0 (0)	0 (0)	0 (0)	0 (0)	0 (0)	0 (0)
Treculia africana	0.2 (0.4)	0.2 (0.4)	0.2 (0.4)	0 (0)	0 (0)	0 (0)	0 (0)	0 (0)	0 (0)	0 (0)	0 (0)	0 (0)	0 (0)	0 (0)	0 (0)
Trichilia gilgiana	1 (1.4)	1.2 (2.2)	1 (1.2)	0.4 (0.5)	0 (0)	0 (0)	0.2 (0.4)	0 (0)	0 (0)	0 (0)	0 (0)	0 (0)	0 (0)	0 (0)	0 (0)
Trichilia monadelpha	0.6 (0.9)	0.6 (0.9)	0.4 (0.5)	0 (0)	0 (0)	0 (0)	0 (0)	0 (0)	0 (0)	0 (0)	0 (0)	0 (0)	0 (0)	0 (0)	0 (0)
Trichilia prieuriana	1.6 (1.8)	1.2 (0.8)	0.4 (0.9)	0 (0)	0.2 (0.4)	0 (0)	0 (0)	0 (0)	0 (0)	0 (0)	0 (0)	0 (0)	0 (0)	0 (0)	0 (0)
Trichilia rubescens	1.2 (1.3)	0 (0)	0 (0)	0 (0)	0 (0)	0 (0)	0 (0)	0 (0)	0 (0)	0 (0)	0 (0)	0 (0)	0 (0)	0 (0)	0 (0)
Trichilia sp.	2.4 (1.9)	0 (0)	0.6 (0.9)	0.4 (0.9)	0 (0)	0 (0)	0 (0)	0 (0)	0 (0)	0 (0)	0 (0)	0 (0)	0 (0)	0 (0)	0 (0)
Trichilia tessmannii	0.2 (0.4)	0 (0)	0 (0)	0 (0)	0 (0)	0 (0)	0 (0)	0 (0)	0 (0)	0 (0)	0 (0)	0 (0)	0 (0)	0 (0)	0 (0)
Trichilia welwitschii	0.6 (0.9)	0.2 (0.4)	0.2 (0.4)	0 (0)	0 (0)	0 (0)	0 (0)	0 (0)	0 (0)	0 (0)	0 (0)	0 (0)	0 (0)	0 (0)	0 (0)
Tridesmostemon claessensii	2.2 (3.9)	2.4 (3.8)	1.8 (2.4)	1.4 (1.7)	0.2 (0.4)	0.2 (0.4)	0 (0)	0 (0)	0 (0)	0 (0)	0 (0)	0 (0)	0 (0)	0 (0)	0 (0)
Tridesmostemon omphalocarpoides	3.6 (4.5)	1.6 (3)	1.2 (1.8)	0.6 (0.9)	0.4 (0.9)	0.4 (0.5)	0 (0)	0.2 (0.4)	0.2 (0.4)	0 (0)	0 (0)	0 (0)	0 (0)	0 (0)	0 (0)

Tridesmostemon sp.	0 (0)	0 (0)	0 (0)	0.2 (0.4)	0 (0)	0 (0)	0 (0)	0 (0)	0 (0)	0 (0)	0 (0)	0 (0)	0 (0)	0 (0)	0 (0)
Trilepisium madagascariense	2 (1.9)	0.8 (1.8)	1 (1.7)	0.4 (0.9)	0.2 (0.4)	0 (0)	0 (0)	0 (0)	0.2 (0.4)	0 (0)	0 (0)	0 (0)	0 (0)	0 (0)	0 (0)
Turraeanthus africanus	3.6 (4.2)	1.6 (1.3)	0.4 (0.5)	0.6 (0.9)	0.4 (0.9)	0 (0)	0 (0)	0 (0)	0 (0)	0 (0)	0 (0)	0.2 (0.4)	0 (0)	0 (0)	0 (0)
Uapaca guineensis	0 (0)	0.2 (0.4)	0 (0)	0 (0)	0 (0)	0.2 (0.4)	0 (0)	0 (0)	0 (0)	0 (0)	0 (0)	0 (0)	0 (0)	0 (0)	0 (0)
Vepris sp.	0 (0)	0 (0)	0.2 (0.4)	0 (0)	0 (0)	0 (0)	0 (0)	0 (0)	0 (0)	0 (0)	0 (0)	0 (0)	0 (0)	0 (0)	0 (0)
Vitex congolensis	0 (0)	0 (0)	0 (0)	0 (0)	0 (0)	0.2 (0.4)	0 (0)	0 (0)	0 (0)	0 (0)	0 (0)	0 (0)	0 (0)	0 (0)	0 (0)
Vitex sp.	0.2 (0.4)	0.2 (0.4)	0.2 (0.4)	0 (0)	0 (0)	0 (0)	0 (0)	0 (0)	0 (0)	0 (0)	0 (0)	0 (0)	0 (0)	0 (0)	0 (0)
Vitex welwitschii	1 (1.7)	0.8 (0.4)	0.4 (0.5)	0.2 (0.4)	0 (0)	0 (0)	0 (0)	0 (0)	0 (0)	0 (0)	0 (0)	0 (0)	0 (0)	0 (0)	0 (0)
Xylopia africana	0 (0)	0 (0)	0 (0)	0.2 (0.4)	0 (0)	0 (0)	0 (0)	0 (0)	0 (0)	0 (0)	0 (0)	0 (0)	0 (0)	0 (0)	0 (0)
Xylopia phloiodora	0 (0)	0.2 (0.4)	0 (0)	0 (0)	0 (0)	0 (0)	0 (0)	0 (0)	0 (0)	0 (0)	0 (0)	0 (0)	0 (0)	0 (0)	0 (0)
