# Supplementary material for: Aboveground vs. Belowground Carbon Stocks in African Tropical Lowland Rainforest: Drivers and Implications
Source: PLoS One. 2015 Nov 24;10(11):e0143209. doi: 10.1371/journal.pone.0143209 (PMC4657968; doi:10.1371/journal.pone.0143209)
Supplement: S4 Table — (PDF) [file pone.0143209.s007.pdf]

S4 Table. Tested height-diameter function forms where H is height, D is diameter and a, b and c are constant coefficients to be estimated.

**Equation name**	**Function**	**Reference**
Power	$H = aD^{b}$	Huxley 1932; Enquist 2002
2-parameter exponential	$H = a(1-e^{-bD})$	Meyer 1940
3-parameter exponential	$H = a - b.e^{-cD}$	Pinheiro et al. 1994; Fang & Bailey 1998
Gompertz	$H = ae^{-be^{-cD}}$	Winsor 1932; Richards 1959
Logistic	$H = a/(1+be^{-cD})$	Winsor 1932; Richards 1959
Weibull	$H = a(1-e^{-bD^{c}})$	Yang et al. 1978
