# Supplementary material for: Aboveground vs. Belowground Carbon Stocks in African Tropical Lowland Rainforest: Drivers and Implications
Source: PLoS One. 2015 Nov 24;10(11):e0143209. doi: 10.1371/journal.pone.0143209 (PMC4657968; doi:10.1371/journal.pone.0143209)
Supplement: S5 Table — (PDF) [file pone.0143209.s008.pdf]

S5 Table. Parameterization for the different models functions in Table S4 for Yangambi.

Models	a	b	c	RSE	AIC
Power	3.923 (0.368)	0.475 (0.025)	na	4.264	1089
2-parameter exponential	32.300 (1.287)	0.034 (0.003)	na	4.279	1090
3-parameter exponential	36.358 (2.930)	31.659 (2.166)	0.022 (0.005)	4.221	1085
Gompertz	33.694 (1.886)	1.561 (0.111)	0.036 (0.005)	4.226	1086
Logistic	32.198 (1.456)	2.965 (0.321)	0.052 (0.006)	4.239	1087
Weibull	41.051 (7.880)	0.054 (0.008)	0.740 (0.108)	4.223	1085
