# Supplementary material for: Aboveground vs. Belowground Carbon Stocks in African Tropical Lowland Rainforest: Drivers and Implications
Source: PLoS One. 2015 Nov 24;10(11):e0143209. doi: 10.1371/journal.pone.0143209 (PMC4657968; doi:10.1371/journal.pone.0143209)
Supplement: S6 Table — (PDF) [file pone.0143209.s009.pdf]

S6 Table. Parameterization for the different models functions in Table S4 for Yoko.

Models	a	b	c	RSE	AIC
Power	3.176 (0.208)	0.544 (0.018)	na	5.099	2649
2-parameter exponential	38.201 (1.379)	0.028 (0.002)	na	5.039	2639
3-parameter exponential	42.502 (2.686)	39.147 (2.158)	0.020 (0.003)	4.962	2627
Gompertz	38.431 (1.600)	1.837 (0.075)	0.036 (0.003)	4.964	2627
Logistic	36.701 (1.274)	3.990 (0.264)	0.054 (0.004)	4.965	2628
Weibull	44.628 (4.742)	0.036 (0.065)	0.842 (0.065)	5.011	2635
